# Mitogen-activated protein kinase cascades in *Vitis vinifera*

**DOI:** 10.3389/fpls.2015.00556

**Published:** 2015-07-22

**Authors:** Birsen Çakır, Ozan Kılıçkaya

**Affiliations:** ^1^Department of Horticulture, Faculty of Agriculture, Ege UniversityIzmir, Turkey; ^2^Department of Pharmacetical Biotechnology, Faculty of Pharmacy, Cumhuriyet UniversitySivas, Turkey

**Keywords:** MAP kinase, *Vitis vinifera*, signal transduction, protein phosphorylation

## Abstract

Protein phosphorylation is one of the most important mechanisms to control cellular functions in response to external and endogenous signals. Mitogen-activated protein kinases (MAPK) are universal signaling molecules in eukaryotes that mediate the intracellular transmission of extracellular signals resulting in the induction of appropriate cellular responses. MAPK cascades are composed of four protein kinase modules: MAPKKK kinases (MAPKKKKs), MAPKK kinases (MAPKKKs), MAPK kinases (MAPKKs), and MAPKs. In plants, MAPKs are activated in response to abiotic stresses, wounding, and hormones, and during plant pathogen interactions and cell division. In this report, we performed a complete inventory of MAPK cascades genes in *Vitis vinifera*, the whole genome of which has been sequenced. By comparison with MAPK, MAPK kinases, MAPK kinase kinases and MAPK kinase kinase kinase kinase members of *Arabidopsis thaliana*, we revealed the existence of 14 MAPKs, 5 MAPKKs, 62 MAPKKKs, and 7 MAPKKKKs in *Vitis vinifera*. We identified orthologs of *V. vinifera* putative MAPKs in different species, and ESTs corresponding to members of MAPK cascades in various tissues. This work represents the first complete inventory of MAPK cascades in *V. vinifera* and could help elucidate the biological and physiological functions of these proteins in *V. vinifera*.

## Introduction

Mitogen-activated protein kinase (MAPK) cascades are higly conserved modules of signal transduction in eucaryotes including yeast, animals, and plants. MAPK cascades play an important role in protein phosphorylation of signal transduction events (Rodriguez et al., 2010). MAPK cascades typically consist of three protein kinases, MAPK, MAPK kinase (MAPKK), and MAPK kinase kinase (MAPKKK), but sometimes include MAP3K kinase (MAP4K) that phosphorylate the corresponding downstream substrates (Jonak et al., 2002; Champion et al., 2004).

MAPK is activated via phophorylation of conserved threonine (T) and tyrosine (Y) residues in the catalytic subdomain by its specific MAPKK, which is in turn activated by phophorylation of two serine/threonine residues in a conserved S/T-X33-5-S/T motif by an upstream MAPKKK (Stulemeijer et al., 2007; Zaïdi et al., 2010; Huang et al., 2011). Upon activation, the MAPK could be translocated into the nucleus or cytoplasm to trigger the cellular responses through phosphorylation of downstream transcription factors or components of transcription machinery while some MAP kinases, like ERK3, are constitutively present in the nucleus and may function in the nucleus (Lee et al., 2004; Pedley and Martin, 2005; Fiil et al., 2009; Nadarajah and Sidek, 2010). MAPKKK is usually activated by a G protein, but sometimes activation is mediated via an upstream MAP4K (Champion et al., 2004).

MAPK proteins contain 11 evolutionary conserved kinase domains that may be involved in substrate specifity or protein-protein interaction (Nadarajah and Sidek, 2010). MAPK cascade proteins have TEY or TDY phophorylation motifs in the region between kinase domains VII and VIII (Group et al., 2002), which provides a protein-binding domain for the activation of MAPKs (Rohila and Yang, 2007).

In plants, MAPKs are involved in cellular responses to hormones, plant growth and development, regulation of the cell cycle, and responses to biotic and abiotic stresses (Jonak et al., 1993; Wilson et al., 1997; Zhang and Klessig, 1997; Bögre et al., 1999; Nishihama et al., 2001; Bergmann et al., 2004; Lukowitz et al., 2004; Katou et al., 2005; Meng et al., 2012).

A variety of genes encoding MAPKs have been cloned from *Arabidopsis*, rice, tobacco and barley, and oat (Huttly and Phillips, 1995; Knetsch et al., 1996; Mizoguchi et al., 1998; Nadarajah and Sidek, 2010; Zaïdi et al., 2010; Sun et al., 2014). The *Arabidopsis* genome contains 20 MAPK genes (Group et al., 2002; Jonak et al., 2002). MAPK genes such as AtMPK4 and AtMPK6, have been identified in *Arabidopsis* (Ichimura et al., 1998, 2000; Nadarajah and Sidek, 2010). It has been reported that MAPK genes are involved in biotic and abiotic stress responses (Mizoguchi et al., 1996; Ichimura et al., 2000; Asai et al., 2002; Nadarajah and Sidek, 2010). For example, OsMAPK3, OsMAPK6, and the MAPK kinase OsMKK4 are induced by a chitin elicitor in rice and the activated form of OsMKK4 induces cell death (Kishi-Kaboshi et al., 2010). Similarly, NtWIPK, OsMPK5, and AtMPK3 were activated by pathogens and abiotic stresses (Zhang and Klessig, 2001; Hamel et al., 2006; Rohila and Yang, 2007). AtMPK4 and AtMPK6 are activated by osmotic stress, low humidity, low temperature, and wounding (Ichimura et al., 2000; Teige et al., 2004). AtMPK3 and AtMPK6 are also regulated by biotic elicitors via AtMKK4/5 and AtMPK4 is a negative regulator of defense response (Asai et al., 2002). In addition, AtMPK3 and AtMPK6 are involved in the embryo, anther and inflorescence development and stomatal distribution on the leaf surface (Bergmann et al., 2004; Gray and Hetherington, 2004; Bush and Krysan, 2007).

MKKs are activated by the phosphorylation on conserved serine and threonine residues in the S/T-X3-5-S/T motif and characterized by a putative MAPK-docking domain K/R-K/R-K/R-X1-6-L-X-L/V/S, and a kinase domain (Group et al., 2002). To date, many MAPKKs have been identified from several plant species. All the identified MAPKK genes from *Arabidopsis*, rice and poplar contain 11 catalytic subdomains (Ichimura et al., 2002; Rao et al., 2010; Wang et al., 2014c). In *Arabidopsis*, MKK1 was activated by wounding and abiotic stress (Matsuoka et al., 2002). Alfalfa SIMKK mediates both salt and elicitor-induced signals (Kiegerl et al., 2000; Cardinale et al., 2002). NtMEK2 activates SIPK and WIPK resulting in cell death (Yang et al., 2001).

MAPKKKs form the largest class of MAPK cascade enzymes with 80 members classified into three subfamilies, MEKK, Raf, and ZIK containing 21, 11, and 48 genes, respectively in *Arabidopsis* (Jonak et al., 2002). Plant MAPKKKs are characterized by different primary structures of their kinase domains, but are conserved within a single group (Champion et al., 2004). The MEKK subfamily comprises a conserved kinase domain of G(T/S)Px(W/Y/F)MAPEV (Jonak et al., 2002). The ZIK subfamily contains GTPEFMAPE(L/V)Y while the Raf subfamily has GTxx(W/Y)MAPE (Jonak et al., 2002). All the MAPKKK proteins have a kinase domain, and most of them have a serine/threonine protein kinase active site (Wang et al., 2015). In the RAF subfamily, most of the proteins have a long N-terminal regulatory domain and C-terminal kinase domain. By contrast, majority of the members in the ZIK subfamily have an N-terminal kinase domain (Wang et al., 2015). However, the MEKK subfamily has a less conserved protein structure with a kinase domain located either at the C- or N-terminal or in the central part of the protein (Wang et al., 2015). Homologs of MAPKKKs have been identified in plant species such as alfalfa, *Arabidopsis*, tobacco (Kovtun et al., 2000; Nishihama et al., 2001; Lukowitz et al., 2004; Nakagami et al., 2004). The MEKK subfamily contains NPK1, NbMAPKKKα, NbMAPKKKγ, NbMAPKKKε in tobacco (Jin et al., 2002; del Pozo et al., 2004; Liu et al., 2004; Melech-Bonfil and Sessa, 2010), MEKK1 in *Arabidopsis* (Asai et al., 2002), and SIMAPKKKα and SIMAPKKKε in tomato (Oh et al., 2010; Sun et al., 2014). The second subfamily, Raf, includes *Arabidopsis* CTR1/raf1 (Kieber et al., 1993), EDR/Raf2 (Frye et al., 2001), and DSM1 in rice (Ning et al., 2010). In *Arabidopsis*, MEKK1 regulates defense responses against different pathogens including bacteria and fungi (Asai et al., 2002; Qiu et al., 2008; Galletti et al., 2011). In addition, AtEDR1, a Raf-like MAPKKK, regulates SA-inducible defense responses (Frye et al., 2001). The ZIK subfamily which contains 10 and 9 members in *Arabidopsis* and rice, respectively, are able to regulate flowering time and circadian rhythms (Wang et al., 2008; Kumar et al., 2011).

A putative phosphorylation domain T/Sx_5_T/S is found between domains VII and VIII in MAP4Ks, which is identical to the phosphorylation motif of MAPKKs from plants (Jouannic et al., 1999; Ichimura et al., 2002). Both domains participate in peptide-substrate recognition (Champion et al., 2004). MAP4Ks can be linked to the plasma membrane through association with a small GTPase or lipid (Qi and Elion, 2005). They are directly activated by stimulated interaction with adaptor proteins (Qi and Elion, 2005). The MAP4Ks are divided into eight classes including PAK-related, Gck, Mst, Tao, Ste/PAK, Sok (Champion et al., 2004). The majority of MAP4Ks are from the large class of Ste20 protein kinases, which exhibit a highly diverse noncatalytic domain (Dan et al., 2001). The PAKs, which have a C-terminal catalytic domain, are separated from the GC Kinase-related polypeptides, which contain an N-terminal catalytic domain (Dan et al., 2001). Most of the MAP4Ks contain an N-terminal catalytic domain, but members of the STE20/PAK group have a C-terminal kinase domain and some plant MAP4Ks have their kinase domain in the middle of the sequences (Leprince et al., 1999). The *Arabidopsis* genome contains 10 putative MAP4Ks (Champion et al., 2004). A maize gene encoding MIK is a GCK-like kinase being a subfamily of MAP4K (Llompart et al., 2003), which relates membrane-located receptors to MAP kinases (Dan et al., 2001). Some MAP4K are able to phosphorylate MEKK or Raf members whereas other MAP4Ks either phosphorylate MAPKKs or function as adaptors (Champion et al., 2004).

However, the functions of most MAPK genes in plants are still unknown. Although MAPK cascades are involved in signaling multiple defense responses, the role of *Vitis* MAPK cascades in response to biotic and abiotic stresses are not elucidated. In previous studies in grapevine, a few components of the MAPK gene family were isolated (Wang et al., 2014a). In addition, the gene family of MAPKKKs were identified and their expression profiles were analyzed in different organs in response to different stresses (Wang et al., 2014b). Interestingly, the expression of *VvMAP* kinase gene was induced by salinity and drought (Daldoul et al., 2012). However, the MAPKK and the MAPKKKK subfamilies have not yet been characterized. To explore the role of MAPK cascade proteins in biotic and abiotic stress responses in grapevine, the publicly available grapevine genome (Jaillon et al., 2007) was analyzed to identify all members of MAPK cascade proteins. Using these databases, we characterized all members of MAPK cascades of *V. vinifera* and performed a phylogenetic analysis in comparison with members of *Arabidopsis* MAPK cascade proteins.

## Materials and methods

### Genome-wide identification of MAPK cascade genes in grapevine

The MAPK cascade protein sequences of *Arabidopsis thaliana* were used to search against the *V. vinifera* proteome 12× database (http://www.genoscope.cns.fr/externe/GenomeBrowser/Vitis/) using a BLASTP analysis (http://www.ncbi.nlm.nih.gov/blast) (Altschul et al., 1990) with scores higher than 400 and an “E” value > e-120 (Çakır and Kılıçkaya, 2013). The sequences of Arabidopsis MAPK cascade proteins were obtained from the TAİR (http://www.arabidopsis.org/). MAPK domain (PS01351), ATP-binding domain (PS00107), protein kinase domain (PS50011), serine/threonine protein kinase active site (PS00108) were identified in the sequences of polypeptides corresponding to *V. vinifera* MAPK cascade proteins by the Conserved Domain Database (CDD) at NCBI (http://www.ncbi.nlm.nih.gov/Structure/cdd/wrpsb.cgi) and PROSITE (http://prosite.expasy.org/) (Marchler-Bauer et al., 2009). In addition, the NCBI non-redundant protein database was screened with each sequence in order to independently validate the automatic annotation.

### Multiple-sequence alignment and phylogenetic tree construction

Multiple-sequence alignments of the putative MAPK cascade proteins were aligned using CLUSTAL W and subjected to phylogenetic analysis by both the maximum parsimony and distance with neighbor-joining methods with 1000 bootstrap replicates (Saitou and Nei, 1987; Thompson et al., 1994). The phylogenetic tree was illustrated using MEGA5. Because similar results were obtained with both methods, only the single tree retrieved from the distance analysis is discussed in detail.

For MAPK cascade subfamilies from both *V. vinifera* and *A. thaliana*, multiple sequence alignment was performed using the multiple sequence comparison by log-expectation (MUSCLE) alignment tool (http://www.ebi.ac.uk/Tools/msa/muscle/) (Edgar, 2004). The phylogenetic analysis was performed using a neighbor-joining method with 1000 bootstrap replicates andvisualized with MEGA5 software (Tamura et al., 2011). The protein theoretical molecular weight and isoelectric point were predicted using compute pI/MW (http://au.expasy.org/tools).

### Orthology analysis and database search

Orthology analysis was performed using the PHOG web server (http://phylofacts.berkeley.edu/orthologs/) (Datta et al., 2009). The sequences of conserved domains with similarity over 70% and an “E” value of 0.0 were selected as queries. The selected sequences of conserved domains from different species were then used in a BLASTP search against the *V. vinifera* protein sequence database. The best hits were annotated as putative orthologous sequences (Moreno-Hagelsieb and Latimer, 2008).

Expressed sequence tags (ESTs) were identified by BLASTn of the *V. vinifera* expressed sequence tag (EST) database (http://www.ncbi.nlm.nih.gov/dbEST). Using the sequences of all of the MAPK cascade proteins as queries. The positives sequences were then confirmed by alignment with the query ORF.

## Results and discussion

### Genome-wide identification of MAPK cascade genes in *Vitis vinifera*

*Vitis vinifera* MAPK cascade sequences were mined from the grapevine genome proteome 12x database (Jaillon et al., 2007). We identified 88 ORFs encoding putative MAPK cascade proteins containing at least MAPK domain by BLAST searches of the grapevine genome proteome 12× database with the amino acid sequences of the MAPK cascade proteins from *A. thaliana* as queries (Table 1). The completed *Vitis* genome contains 14 MAPKs, 5 MAPKKs, 62 MAPKKKs, and 7 MAPKKKKs (Table 1).

**Table 1:** **Detailed inventory of the *Vitis* MAPK cascade proteins**.

**Subfamily name**	**12X *Vitis vinifera* ID**	**NCBI GenBank ID**	**Chr**	**Str**	**Genomic location**	**Gene length in bp**	**CDS length in bp**	**Length of protein in AA**	**Number of Exon**	**Number of Intron**	**pI**	**mW (kDa)**
**VvMPKs**												
VvMPK1	GSVIVT01000784001	CBI31754.3	12	+	124452–133238	8787	1518	505	10	9	9.34	57.43
VvMPK2	GSVIVT01005924001	CBI35594.3	7	+	886169–898284	12116	1341	446	16	15	5.40	51.22
VvMPK3	GSVIVT01008408001	CBI15552.3	17	+	2368190–2377747	9558	1806	601	11	10	6.89	67.98
VvMPK4	GSVIVT01009766001	CBI19748.3	18	+	11125765–11129338	3574	588	195	4	3	5.44	22.50
VvMPK5	GSVIVT01011749001	CBI26902.3	1	−	4565334–4574753	9420	1842	613	11	10	8.68	70.46
VvMPK6	GSVIVT01014081001	CBI20098.3	19	+	224299–234190	9892	1797	599	10	9	9.21	67.79
VvMPK7	GSVIVT01017873001	CBI26170.3	5	−	4205509–4215917	10409	1692	563	10	9	8.59	64.03
VvMPK8	GSVIVT01018883001	CBI17457.3	4	+	18974001–19005635	31635	2310	769	10	9	5.51	87.48
VvMPK9	GSVIVT01019406001	CBI34380.3	2	−	380310–386888	6579	1128	375	6	5	5.86	42.80
VvMPK10	GSVIVT01022771001	CBI37450.3	2	+	16326975–16335400	8426	1359	452	16	15	9.62	51.58
VvMPK11	GSVIVT01025091001	CBI16237.3	6	+	4580755–4584961	4207	1116	371	6	5	4.94	42.53
VvMPK12	GSVIVT01025105001	CBI16244.3	6	−	4432854–4436338	3485	990	329	6	5	5.52	38.17
VvMPK13	GSVIVT01026984001	CBI40425.3	15	−	18821560–18826926	5367	1128	375	6	5	6.43	43.27
VvMPK14	GSVIVT01038192001	CBI24707.3	5	+	24220238–24241107	20870	993	330	6	5	5.64	38.37
**VvMAPKKs**												
VvMKK1	GSVIVT01008476001	CBI15608.3	17	+	1537423–1538551	1129	675	224	3	2	6.38	24.66
VvMKK2	GSVIVT01015155001	CBI27870.3	11	+	1417439–1424337	6899	1065	355	8	7	6.00	39.28
VvMKK3	GSVIVT01015283001	CBI27984.3	11	+	2377698–2381398	3701	1065	355	8	7	6.02	39.98
VvMKK4	GSVIVT01016115001	CBI25274.3	9	+	19257788–19265261	7474	1188	396	5	4	10.15	43.78
VvMKK5	GSVIVT01032414001	CBI34873.3	14	−	27139003–27145873	6871	1557	519	9	8	5.56	57.61
**VvMAPKKKs**												
VviMAPKKK1	GSVIVT01000047001	CBI36768.3	14	+	3063647–3072319	8673	1992	664	17	16	5.36	72.89
VviMAPKKK2	GSVIVT01000256001	CBI27711.3	7	+	20596048–20597073	1026	921	307	2	1	8.97	33.93
VviMAPKKK3	GSVIVT01001193001	CBI28728.3	7	+	944892–950225	5334	1215	405	6	5	7.03	44.92
VviMAPKKK4	GSVIVT01001690001	CBI35506.3	18	−	14296312–14329573	33262	1653	551	16	15	5.07	61.80
VviMAPKKK5	GSVIVT01002332001	CBI35719.3	Un	+	34161697–34167622	5926	696	232	7	6	9.43	26.14
VviMAPKKK6	GSVIVT01004254001	CBI18826.3	Un	+	37734319–37739476	5158	1158	386	10	9	9.51	43.07
VviMAPKKK7	GSVIVT01007446001	CBI25853.3	Un	+	31988209–31995727	7519	2124	708	11	10	9.73	77.57
VviMAPKKK8	GSVIVT01007637001	CBI14941.3	17	−	10966272–10980533	14262	1464	488	9	8	5.47	54.86
VviMAPKKK9	GSVIVT01007646001	CBI14949.3	17	+	10874999–10877438	2440	1059	353	6	5	8.13	40.08
VviMAPKKK10	GSVIVT01007762001	CBI15038.3	17	+	9308908–9314007	5100	909	303	3	2	7.97	34.37
VviMAPKKK11	GSVIVT01007775001	CBI15048.3	17	−	9166428–9172256	5829	1050	350	6	5	7.02	38.56
VviMAPKKK12	GSVIVT01008413001	CBI15555.3	17	−	2321687–2342403	20717	2697	899	16	15	5.24	99.09
VviMAPKKK13	GSVIVT01008728001	CBI18907.3	18	−	1477098–1491666	14569	1569	523	16	15	6.67	59.46
VviMAPKKK14	GSVIVT01008938001	CBI19081.3	18	−	3594893–3606331	11439	822	274	8	7	6.45	30.15
VviMAPKKK15	GSVIVT01009192001	CBI19282.3	18	−	5939861–5949524	9664	2718	906	15	14	8.41	101.48
VviMAPKKK16	GSVIVT01009575001	CBI19581.3	18	+	9549009–9561275	12267	1101	367	10	9	8.39	40.91
VviMAPKKK17	GSVIVT01012031001	CBI27127.3	1	−	2006896–2039042	32147	4191	1397	25	24	5.73	154.60
VviMAPKKK18	GSVIVT01012116001	CBI27196.3	1	+	1303636–1315494	11859	3717	1239	11	10	5.32	136.73
VviMAPKKK19	GSVIVT01012632001	CBI23172.3	10	+	222774–228777	6004	1287	429	4	3	5.48	48.37
VviMAPKKK20	GSVIVT01012686001	CBI23211.3	10	+	641937–648890	6954	3369	1123	8	7	6.09	123.96
VviMAPKKK21	GSVIVT01012895001	CBI25598.3	11	+	6576324–6581862	5539	1470	490	9	8	5.40	54.29
VviMAPKKK22	GSVIVT01015494001	CBI28162.3	11	−	4194704–4202353	7650	3066	1022	7	6	8.36	114.09
VviMAPKKK23	GSVIVT01017915001	CBI26208.3	5	+	4619229–4633132	13904	2061	687	17	16	6.76	75.81
VviMAPKKK24	GSVIVT01017968001	CBI26245.3	5	+	5145293–5148759	3467	1632	544	11	10	5.43	63.24
VviMAPKKK25	GSVIVT01018020001	CBI26291.3	5	−	5521761–5528578	6818	1371	457	7	6	8.32	51.27
VviMAPKKK26	GSVIVT01018052001	CBI26318.3	5	+	5824109–5839208	15100	3354	1118	11	10	5.59	124.59
VviMAPKKK27	GSVIVT01019010001	CBI17559.3	4	+	17821355–17829046	7692	906	302	5	4	6.66	33.93
VviMAPKKK28	GSVIVT01019630001	CBI34567.3	2	+	2093246–2099598	6353	1791	597	16	15	6.59	68.4
VviMAPKKK29	GSVIVT01019739001	CBI34657.3	2	+	2874833–2882840	8008	2706	902	11	10	9.12	97.91
VviMAPKKK30	GSVIVT01019821001	CBI34722.3	2	+	3631707–3639546	7840	2088	696	15	14	6.37	78.14
VviMAPKKK31	GSVIVT01020712001	CBI21988.3	12	+	2837402–2887099	49698	2454	818	11	10	5.99	91.70
VviMAPKKK32	GSVIVT01021854001	CBI34208.3	14	−	6462432–6466040	3609	1821	607	10	9	5.22	69.40
VviMAPKKK33	GSVIVT01021884001	CBI34231.3	14	−	6026347–6048725	22379	2631	877	16	15	5.50	97.14
VviMAPKKK34	GSVIVT01022098001	CBI21399.3	7	+	16573422–16578723	5302	1287	429	8	7	5.31	47.06
VviMAPKKK35	GSVIVT01022115001	CBI21414.3	7	−	16707463–16710545	3083	1233	411	8	7	4.66	44.78
VviMAPKKK36	GSVIVT01022116001	CBI21415.3	7	+	16711209–16721450	10242	2469	823	7	6	8.72	89.41
VviMAPKKK37	GSVIVT01023037001	CBI23895.3	12	+	16524280–16552240	27961	1296	432	11	10	7.05	48.78
VviMAPKKK38	GSVIVT01023048001	CBI23901.3	12	+	16381443–16399280	178238	525	175	5	4	8.42	20.19
VviMAPKKK39	GSVIVT01023216001	CBI29680.3	12	+	21019776–21020867	1092	1092	364	1	0	8.88	39.99
VviMAPKKK40	GSVIVT01023958001	CBI37812.3	3	−	2141138–2162161	21024	1743	581	16	15	5.56	65.53
VviMAPKKK41	GSVIVT01024578001	CBI15829.3	6	−	8664971–8669192	4222	1896	632	7	6	6.83	71.74
VviMAPKKK42	GSVIVT01026487001	CBI37539.3	4	+	22814276–22820209	5934	1803	601	12	11	8.99	65.01
VviMAPKKK43	GSVIVT01026546001	CBI37576.3	4	+	21993962–21997860	3899	1701	567	10	9	5.52	64.16
VviMAPKKK44	GSVIVT01027189001	CBI40585.3	15	−	17151471–17154829	3359	1776	592	10	9	5.82	67.45
VviMAPKKK45	GSVIVT01028897001	CBI22687.3	16	−	17707492–17719297	11806	2679	893	11	10	9.43	95.93
VviMAPKKK46	GSVIVT01029055001	CBI33351.3	5	+	11545076–11551320	6345	2859	953	13	12	5.42	105.79
VviMAPKKK47	GSVIVT01029147001	CBI17788.3	11	+	19186659–19204657	17999	2937	979	11	10	5.40	108.81
VviMAPKKK48	GSVIVT01029426001	CBI35320.3	17	+	17077436–17089089	11654	555	185	4	3	5.62	21.36
VviMAPKKK49	GSVIVT01030044001	CBI28411.3	12	−	9089290–9097431	8142	2496	832	8	7	8.80	93.29
VviMAPKKK50	GSVIVT01030194001	CBI18047.3	8	+	10650328–10661181	10854	1014	338	6	5	8.85	37.96
VviMAPKKK51	GSVIVT01030202001	CBI18051.3	8	−	10519384–10524077	4694	1881	627	7	6	5.29	71.71
VviMAPKKK52	GSVIVT01031721001	CBI32391.3	3	−	3812818–3816504	3687	1194	398	8	7	8.32	43.57
VviMAPKKK53	GSVIVT01032232001	CBI24046.3	11	+	13477774–13485357	7584	447	149	3	2	5.90	16.90
VviMAPKKK54	GSVIVT01032389001	CBI34850.3	14	−	26886951–26893203	6253	1059	353	6	5	6.90	39.53
VviMAPKKK55	GSVIVT01032487001	CBI34936.3	14	+	27812667–27819608	6942	1083	361	6	5	6.34	40.52
VviMAPKKK56	GSVIVT01033779001	CBI30245.3	8	−	17884683–17902051	17369	2238	746	17	16	6.14	82.96
VviMAPKKK57	GSVIVT01034710001	CBI40217.3	13	−	8150739–8202754	52016	2277	759	15	14	7.89	85.25
VviMAPKKK58	GSVIVT01034988001	CBI22876.3	5	−	694778–701765	6988	2199	733	14	13	6.42	81.58
VviMAPKKK59	GSVIVT01035409001	CBI20668.3	4	+	1079723–1090460	10738	2631	877	15	14	5.26	97.28
VviMAPKKK60	GSVIVT01036758001	CBI24172.3	19	−	22924599–22935504	10906	3138	1046	10	9	5.23	115.67
VviMAPKKK61	GSVIVT01037773001	CBI26734.3	19	−	7730105–7732118	2014	894	298	2	1	6.17	34.22
VviMAPKKK62	GSVIVT01038760001	CBI32969.3	12	+	610174–641062	30889	1476	492	8	7	6.05	55.76
**VvMAPKKKKs**												
VvMAP4K1	GSVIVT01012233001	CBI27303.3	5	+	7076729–7078360	1632	1362	454	4	3	5.20	88.93
VvMAP4K2	GSVIVT01013739001	CBI28527.3	9	−	18395010–18410644	15635	1704	568	15	14	9.31	41.78
VvMAP4K3	GSVIVT01014297001	CBI20268.3	1	−	8012586–8021182	8597	1107	369	9	8	6.34	78.12
VvMAP4K4	GSVIVT01016074001	CBI25246.3	14	−	27646939–27657107	10169	1803	601	16	15	5.58	62.83
VvMAP4K5	GSVIVT01019643001	CBI34578.3	2	−	2150787–2160118	9332	2190	730	22	21	6.68	81.13
VvMAP4K6	GSVIVT01027718001	CBI23577.3	1	−	342583–373752	31170	2430	810	19	18	5.80	50.65
VvMAP4K7	GSVIVT01032461001	CBI34913.3	19	−	2416077–2441445	25369	2121	707	20	19	5.81	67.08

*The identified open reading frames (ORFs) are classified into four subfamilies, MAPK, MAPKK, MAPKKK, and MAPKKKK. Columns 1–13 contain the protein acronym (Name), coding sequence (CDS), Vitis proteome 12× ID, GenBank ID, chromosome location (Chr), strand (str), gene length, number of introns and exons, protein length, estimates of molecular weight, and isoelectric point of the protein (pI) for each gene are given*.

### Phylogenetic analysis

**Figure 1:**
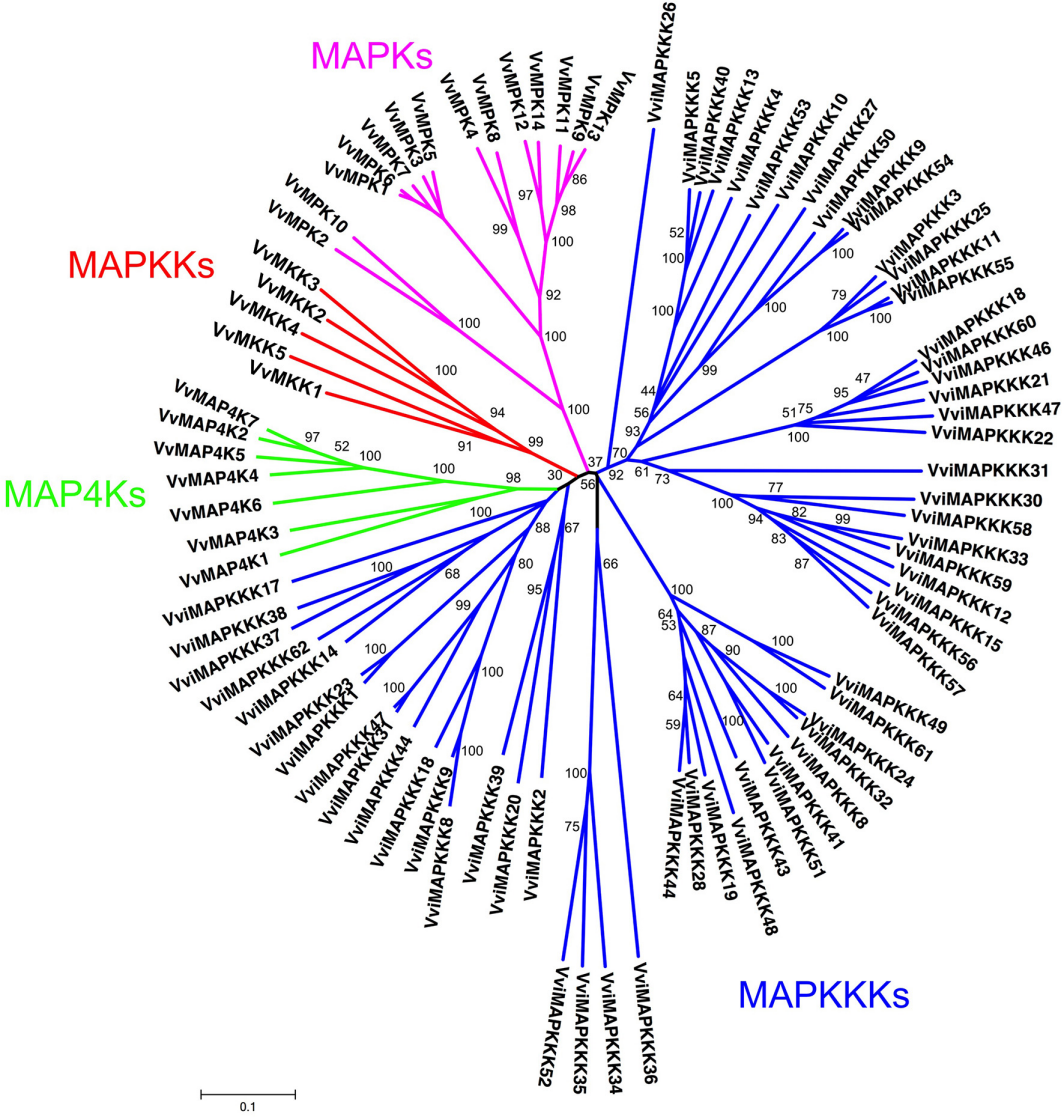
**Construction of phylogenetic tree of *Vitis* MAPK cascade proteins**. The amino sequences of all *Vitis* ABC proteins were aligned using the ClustalW program and were subjected to phylogenetic analysis by the distance with neighbor-joining method. The reliabilities of each branch point, as assessed by the analysis of 1000 computer-generated trees (bootstrap replicates), were in excess of 90%, except for those discussed in the text. The abbreviations of MAPK cascade proteins are as follows: MAPK, Mitogen-activated Protein Kinase; MAPKK, MAPK Kinase; MAPKKK, MAPKK Kinase; MAPKKKK, MAPKKK Kinase as described in the text.

All predicted MAPK cascade family sequences were aligned using ClustalW (Thompson et al., 1994). A rooted phylogenetic tree was constructed by alignment of full length amino acid sequences using the MEGA5 program and maximum parsimony and distance with neighbor-joining methods (Saitou and Nei, 1987) (Figure 1). One thousand bootstrap replicates were produced for each analysis.

*Vitis* MAPK cascade sequences can be divided into four subfamilies on the basis of the presence of conserved threonine and tyrosine residues in the motif TxY located in the activation loop (T-loop) between kinase subdomains VII and VIII. In addition, we identified MAPKKKK subfamily with 7 members in *Vitis* genome, which has the conserved amino acid motifs TFVGTPxWMAPEV as described (Jonak et al., 2002). The members of four subfamilies clustered more tightly with each other than with members of other subfamilies (Figure 1).

### MAPKs

The phylogenetic analysis showed that the VvMAPKs were devided into five distinct groups, which is higher than previous reports (Kumar and Kirti, 2010; Nadarajah and Sidek, 2010). Group V MAPKs are found only in the grapevine genome among other plant species. All of identified ORFs encoding MAPK were named VvMPK1 through 14. Hyun et al. (2010) reported 12 MAPKs based on 8x sequence coverage in grapevine genome whereas we identified a total of 14 ORFs in *Vitis* 12x genome coverage (Hyun et al., 2010), which may be due to the errors corrected in 12x genome sequence coverage. The grapevine genome contains less MAPKs than *Arabidopsis* (20 MAPKs) (Ichimura et al., 2002) and rice (17 MAPKs) (Liu and Xue, 2007). Members of the *Vitis* MAPK subfamily show 20–86% identity to each other. Full length MAPK proteins ranged in size from 195 to 769 amino acids (Table 1). Variation in length of the entire MAPK gene is usually due to differences in the length of MAPK domain and/or, due to the number of introns. The difference in length among *MAPK* genes may indicate the presence or absence of motifs which could affect functional specifity.

**Figure 2:**
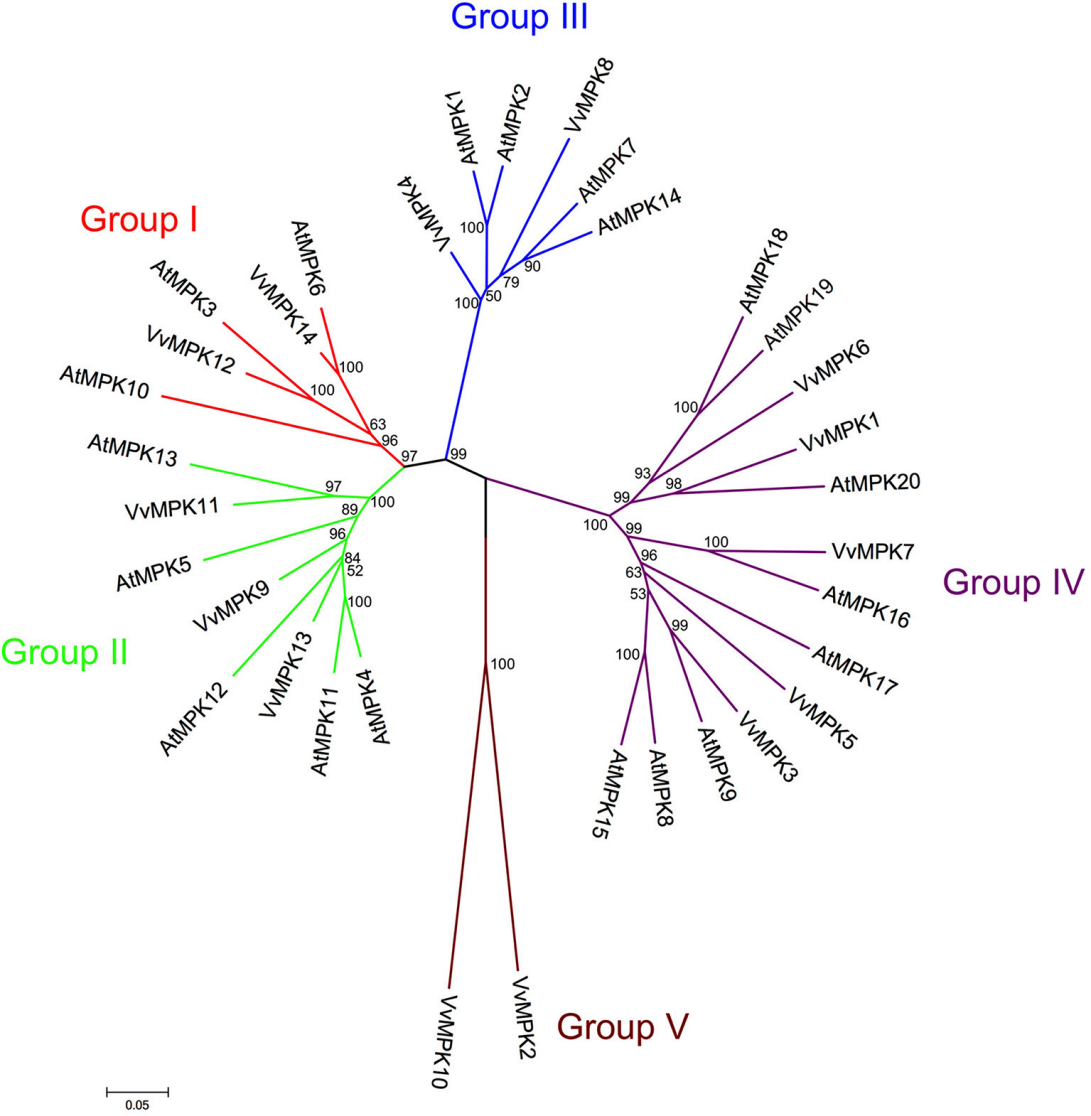
**Phylogenetic relationship of *Arabidopsis* and *Vitis* MAPK proteins**. The amino acid sequences of all *Arabidopsis* MAPK proteins and those of *Vitis vinifera* were aligned using the MUSCLE program and subjected to phylogenetic analysis by the distance with neighborjoining method using MEGA5 programme. Accession numbers for *Arabidopsis* sequences are AtMPK1 (At1g10210), AtMPK2 (At1g59580), AtMPK3 (At3g45640), AtMPK4 (At4g01370), AtMPK5 (At4g11330), AtMPK6 (At2g43790), AtMPK7 (At2g18170), AtMPK8 (At1g18150), AtMPK9 (At3g18040), AtMPK10 (At3g59790), AtMPK11 (At1g01560), AtMPK12 (At2g46070), AtMPK13 (At1g07880), AtMPK14 (At4g36450), AtMPK15 (At1g73670), AtMPK16 (At5g19010), AtMPK17 (At2g01450), AtMPK18 (At1g53510), AtMPK19 (At3g14720), AtMPK20 (At2g42880).

VvMPK12, VvMPK14 belong to the group I., which contains well-characterized *MAPK* genes including *AtMPK3, AtMPK6* (Figure 2). It has been demonstrated that *AtMPK3, OsMPK5* were activated in response to pathogens and abiotic stresses (Zhang and Klessig, 2001; Hamel et al., 2006; Rohila and Yang, 2007). *OsMPK5* plays an important role for the resistance to blast disease (Song and Goodman, 2002; Huang et al., 2011). *AtMPK6* can be activated by various abiotic and biotic stresses (Ichimura et al., 2000; Yuasa et al., 2001; Feilner et al., 2005; Huang et al., 2011). Similarly, *PtrMAPK* is involved in resistance to both dehydration and cold (Huang et al., 2011).

Group II MAPKs are involved in both abiotic stresses and cell division in *Arabidopsis*. VvMPK13, VvMPK11, and VvMPK9 are clustered with Group II., which includes AtMPK4, AtMPK5, AtMPK12, and AtMPK11. AtMPK4 and its upstream MAPKK AtMKK2 can be activated by biotic and abiotic stresses (Ichimura et al., 2000; Teige et al., 2004).

VvMPK4 and VvMPK8 belong to group III. AtMPK1 in the group III is regulated by salt stress treatment (Mizoguchi et al., 1996). In addition, AtMPK1 and AtMPK2 are activated by ABA (Ortiz-Masia et al., 2007). The group III genes, such as rice BWMK1 and alfalfa TDY1, are activated by wounding and pathogens (Nowak et al., 1997; Lynch et al., 2001).

Group IV, which includes VvMPK1, VvMPK3, VvMPK5, VvMPK6, and VvMPK7 of the *Vitis* MAPKs, have the TDY motif in their T-loop and the absence of the C-terminal CD domain, which is consistently found in members of the other MAPK groups. VvMPK2 and VvMPK10 belonging to group V were separated from other groups.

The orthology analysis program identified one hundred-fourteen orthologs from various plant species for this subfamily (Table 2). The VvMPK3 amino acid sequence shows 83% similarity with AtMPK9, and VvMPK12 shows 84% similarity with AtMPK3 from *A. thaliana*. The members of VvMAPK subfamily share between 75.8 and 91.8% similarity to the MAPK members from *Ricius communis, Oryza sativa*, and *A. thaliana*. The phylogenetic analysis of *A. thaliana* and *V. vinifera* MAPK subfamilies confirmed the orthologs of VvMPK14/AtMPK6, VvMPK12/AtMPK3, VvMAPK11/AtMAPK13, VvMPK13/AtMPK12, VvMPK7/AtMPK16, and VvMPK3/AtMPK9 (Figure 2).

**Table 2:** **Orthologs of *Vitis* MAPK cascade proteins identified in diverse plant species**.

**Subfamily name**	***Vitis* proteome 12× ID**	**Species**	**%ID**	**UniprotKB ID**
*VvMPK1*	GSVIVT01000784001	*Ricinus communis*	82.7	B9H811_POPTR
*VvMPK2*	GSVIVT01005924001	*Ricinus communis*	75.8	B9SYK7_RICCO
*VvMPK3*	GSVIVT01008408001	*Populus trichocarpa*	84.0	B9I2G2_POPTR
		*Brassica napus*	82.0	Q5XU40_BRANA
		*Arabidopsis thaliana*	81.8	MPK9_ARATH
		*Arabidopsis lyrata* subsp. *Lyrata*	81.2	D7L7Z0_ARALL
		*Oryza sativa subsp. Indica*	77.6	B3GCL0_ORYSI
		*Ricinus communis*	76.6	B9T7E7_RICCO
		*Zea mays*	75.7	B4F907_MAIZE
		*Oryza sativa* subsp*. Japonica*	75.6	MPK16_ORYSJ
*VvMPK6*	GSVIVT01014081001	*Ricinus communis*	80.1	B9SR58_RICCO
		*Populus trichocarpa*	78.7	B9GGZ4_POPTR
*VvMPK7*	GSVIVT01017873001	*Ricinus communis*	90.9	B9RSS7_RICCO
		*Populus trichocarpa*	90.2	B9HY78_POPTR
		*Arabidopsis lyrata* subsp*. Lyrata*	84.7	D7LYJ6_ARALL
		*Arabidopsis thaliana*	84.5	MPK16_ARATH
		*Oryza sativa* subsp*. Japonica*	83.6	MPK15_ORYSJ
		*Oryza sativa* subsp*. Indica*	83.2	B3GCK9_ORYSI
		*Zea mays*	79.9	Q6TAR9_MAIZE
		*Triticum aestivum*	77.9	A9RAB1_WHEAT
*VvMPK9*	GSVIVT01019406001	*Populus trichocarpa*	91.4	B9GWV0_POPTR
		*Papaver rhoeas*	89.3	Q683Y4_9MAGN
		*Ricinus communis*	87.4	B9RCG7_RICCO
		*Solanum tuberosum*	84.8	Q8LT16_SOLTU
		*Sorghum bicolor*	82.7	C5YH06_SORBI
		*Medicago truncatula*	81.9	B7FK53_MEDTR
*VvMPK10*	GSVIVT01022771001	*Populus trichocarpa*	75.7	B9GZV6_POPTR
*VvMPK11*	GSVIVT01025091001	*Nicotiana attenuate*	86.6	A5H7H6_NICAT
		*Ricinus communis*	86.0	B9SW68_RICCO
		*Malus domestica*	85.3	D1MFM2_MALDO
		*Solanum lycopersicum*	83.5	E2GLN8_SOLLC
		*Nicotiana benthamiana*	83.5	B2NIC1_NICBE
		*Medicago sativa*	82.7	Q9ZP91_MEDSA
		*Nicotiana tabacum*	82.7	NTF6_TOBAC
		*Solanum tuberosum*	82.7	Q8LT15_SOLTU
		*Arabidopsis lyrata* subsp*. Lyrata*	80.4	D7KHW6_ARALL
		*Arabidopsis thaliana*	79.8	MPK13_ARATH
*VvMPK12*	GSVIVT01025105001	*Citrus sinensis*	90.3	A2IB54_CITSI
		*Populus trichocarpa*	90.0	B9HNK3_POPTR
		*Catharanthus roseus*	89.7	B8LFE0_CATRO
		*Cucumis sativus*	89.3	Q0R4I2_CUCSA
		*Solanum lycopersicum*	86.9	Q84MI4_SOLLC
		*Medicago truncatula*	86.8	B7FJD9_MEDTR
		*Solanum peruvianum*	86.6	A8VJL7_SOLPE
		*Solanum tuberosum*	86.6	Q3V6C4_SOLTU
		*Nicotiana attenuate*	86.6	A5H2L1_NICAT
		*Ricinus communis*	86.4	B9T1Z7_RICCO
		*Capsicum annuum*	86.3	Q9LKZ2_CAPAN
		*Nicotiana benthamiana*	86.3	Q8H0B4_NICBE
		*Nicotiana tabacum*	86.0	Q8W406_TOBAC
		*Pisum sativum*	86.0	Q9M6S1_PEA
		*Brassica napus*	86.0	Q5IV18_BRANA
		*Medicago sativa*	85.7	O24077_MEDSA
		*Petroselinum crispum*	85.7	O04694_PETCR
		*Glycine max*	85.4	Q5K6Q4_SOYBN
		*Arabidopsis thaliana*	84.2	MPK3_ARATH
		*Saccharum officinarum*	78.2	Q4QWQ7_SACOF
		*Oryza sativa* subsp.*iÝndica*	77.6	MPK5_ORYSI
		*Avena sativa*	77.3	Q43379_AVESA
*VvMPK13*	GSVIVT01026984001	*Nicotiana attenuate*	90.7	A5H7H4_NICAT
		*Ricinus communis*	90.5	B9RDW5_RICCO
		*Glycine max*	89.9	C6TEP0_SOYBN
		*Populus trichocarpa*	89.2	B9GQC1_POPTR
		*Malus hupehensis*	89.2	B1N8Y5_9ROSA
		*Petroselinum crispum*	89.0	Q84XZ6_PETCR
		*Nicotiana tabacum*	88.5	Q3C254_TOBAC
		*Solanum lycopersicum*	88.2	D7R517_SOLLC
		*Thellungiella halophile*	87.4	E4MW58_THEHA
		*Brassica napus*	87.3	E3US78_BRANA
		*Arabidopsis thaliana*	87.2	MPK4_ARATH
		*Arabidopsis lyrata* subsp*. Lyrata*	86.9	D7M4W5_ARALL
		*Malus micromalus*	86.4	Q8GZR5_MALMI
		*Medicago sativa*	86.3	MMK2_MEDSA
		*Oryza sativa* subsp*. Ýndica*	83.6	A2Z9P1_ORYSI
		*Oryza sativa* subsp*. Japonica*	83.6	MPK6_ORYSJ
		*Zea mays*	83.2	B4FH09_MAIZE
		*Sorghum bicolor*	82.4	C5WUG0_SORBI
		*Physcomitrella patens* subsp*. patens*	80.9	A9S9Q8_PHYPA
		*Pinus tadea*	78.0	C7ENI4_PINTA
*VvMPK14*	GSVIVT01038192001	*Populus trichocarpa*	95.7	B9HGK0_POPTR
		*Malus domestica*	95.2	D1MFM1_MALDO
		*Pisum sativum*	94.8	MAPK_PEA
		*Ricinus communis*	94.5	B9SFT4_RICCO
		*Medicago sativa*	94.2	MMK1_MEDSA
		*Glycine max*	94.2	Q5K6N6_SOYBN
		*Nicotiana tabacum*	93.3	NTF4_TOBAC
		*Solanum tuberosum*	93.0	Q8LT17_SOLTU
		*Nicotiana benthamiana*	93.0	B3IWK6_NICBE
		*Solanum lycopersicum*	93.0	Q84MI5_SOLLC
		*Capsicum annuum*	92.7	Q9LKZ1_CAPAN
		*Solanum peruvianum*	92.7	B5B2H6_SOLPE
		*Nicotiana attenuate*	92.4	A5H2L0_NICAT
		*Arabidopsis thaliana*	91.8	MPK6_ARATH
		*Arabidopsis lyrata* subsp*. Lyrata*	91.8	D7LKI6_ARALL
		*Brassica napus*	91.5	E1B2J5_BRANA
		*Sorghum bicolor*	90.9	C5Z4D1_SORBI
		*Oryza sativa* subsp*. Japonica*	90.5	MPK1_ORYSJ
		*Zea mays*	90.5	B8QN51_MAIZE
		*Oryza sativa* subsp*. Indica*	90.5	B3GCK7_ORYSI
		*Triticum aestivum*	89.9	Q84XZ3_WHEAT
		*Pinus tadea*	87.5	C7ENI3_PINTA
*VvMPKK2*	GSVIVT01015155001	*Populus trichocarpa*	81.7	B9IKC3_POPTR
		*Ricinus communis*	81.2	B9RK49_RICCO
		*Petroselinum crispum*	79.3	Q6QMT5_PETCR
		*Malus domestica*	77.6	D1MFM3_MALDO
		*Nicotiana tabacum*	76.8	Q9M6Q9_TOBAC
		*Solanum lycopersicum*	76.3	O48616_SOLLC
		*Arabidopsis thaliana*	75.6	C0Z2L0_ARATH
		*Glycine max*	75.1	Q5JCL0_SOYBN
*VvMKK3*	GSVIVT01015283001	*Ricinus communis*	89.0	B9RKG0_RICCO
		*Solanum lycopersicum*	88.7	Q66MH7_SOLLC
		*Nicotiana tabacum*	88.1	Q9AYN9_TOBAC
		*Nicotiana benthamiana*	87.3	B2NIC2_NICBE
		*Origanum onites*	86.1	A7U0S8_9LAMI
		*Arabidopsis thaliana*	83.3	M2K6_ARATH
		*Arabidopsis lyrata* subsp*. Lyrata*	83.1	D7MLT9_ARALL
		*Oryza sativa* subsp*. Japonica*	77.3	M2K1_ORYSJ
		*Oryza sativa* subsp*. Ýndica*	77.3	Q0Z7Z4_ORYSI
		*Zea mays*	77.1	O49975_MAIZE
		*Sorghum bicolor*	76.8	C5XIE1_SORBI
*VvMKK5*	GSVIVT01032414001	*Ricinus communis*	86.3	B9S641_RICCO
		*Populus trichocarpa*	84.6	B9GI57_POPTR
		*Nicotiana tabacum*	82.8	Q40542_TOBAC
		*Suaeda salsa*	79.0	Q8L8I2_SUASA
		*Arabidopsis thaliana*	78.6	O80396_ARATH
*VviMAPKKK3*	GSVIVT01001193001	*Populus trichocarpa*	85.5	B9GSK4_POPTR
		*Ricinus communis*	77.8	B9SFH0_RICCO
*VviMAPKKK4*	GSVIVT01001690001	*Populus trichocarpa*	82.2	B9GTK7_POPTR
		*Ricinus communis*	80.7	B9T446_RICCO
*VviMAPKKK8*	GSVIVT01007637001	*Populus trichocarpa*	80.5	B9I3F6_POPTR
		*Ricinus communis*	79.3	B9RAT5_RICCO
		*Glycine max*	78.7	C0M0P4_SOYBN
		*Medicago sativa*	78.0	Q84RS1_MEDSA
*VviMAPKKK9*	GSVIVT01007646001	*Ricinus communis*	88.3	B9RAU4_RICCO
		*Glycine max*	84.7	C6T9D3_SOYBN
		*Oryza sativa*	82.1	B8AEQ7_ORYSI
		*Zea mays*	82.1	C0P3M4_MAIZE
		*Oryza sativa* subsp*. Japonica*	82.1	Q6ZH81_ORYSJ
		*Arabidopsis thaliana*	81.8	Q9FGS7_ARATH
*VviMAPKKK10*	GSVIVT01007762001	*Populus trichocarpa*	88.0	B9IEQ9_POPTR
		*Ricinus communis*	84.1	B9RB33_RICCO
*VviMAPKKK12*	GSVIVT01008413001	*Populus trichocarpa*	80.8	B9IEA9_POPTR
*VviMAPKKK17*	GSVIVT01012031001	*Ricinus communis*	79.8	B9S4I8_RICCO
		*Arabidopsis thaliana*	76.9	Q9LJD8_ARATH
*VviMAPKKK25*	GSVIVT01018020001	*Populus trichocarpa*	84.0	B9HUS5_POPTR
		*Ricinus communis*	79.4	B9RTM1_RICCO
		*Arabidopsis thaliana*	75.1	Q9LUI6_ARATH
*VviMAPKKK29*	GSVIVT01019739001	*Ricinus communis*	76.3	B9RCD5_RICCO
		*Populus trichocarpa*	76.0	B9GKG5_POPTR
*VviMAPKKK31*	GSVIVT01020712001	*Ricinus communis*	84.0	B9SRD1_RICCO
		*Populus trichocarpa*	82.4	B9IA51_POPTR
*VviMAPKKK34*	GSVIVT01022098001	*Populus trichocarpa*	82.7	B9HHA4_POPTR
*VviMAPKKK40*	GSVIVT01023958001	*Populus trichocarpa*	82.2	B9H1M1_POPTR
		*Ricinus communis*	79.8	B9S5G6_RICCO
*VviMAPKKK42*	GSVIVT01026487001	*Ricinus communis*	77.0	B9SUR2_RICCO
*VviMAPKKK45*	GSVIVT01028897001	*Ricinus communis*	83.2	B9RIV9_RICCO
		*Populus trichocarpa*	82.9	B9IDA8_POPTR
*VviMAPKKK50*	GSVIVT01030194001	*Ricinus communis*	85.4	B9T3P6_RICCO
		*Populus trichocarpa*	84.3	B9IGR7_POPTR
		*Medicago truncatula*	84.0	B7FKS6_MEDTR
		*Glycine max*	81.6	C6TMB8_SOYBN
		*Arabidopsis thaliana*	80.9	Q8L6Y9_ARATH
*VviMAPKKK54*	GSVIVT01032389001	*Populus trichocarpa*	91.2	B9GI75_POPTR
		*Ricinus communis*	89.5	B9S662_RICCO
		*Cucumis sativus*	82.4	Q7XJ65_CUCSA
		*Arabidopsis thaliana*	81.3	Q9LT56_ARATH
*VviMAPKKK55*	GSVIVT01032487001	*Populus trichocarpa*	89.2	B9IJN5_POPTR
		*Ricinus communis*	88.0	B9RB44_RICCO
		*Arabidopsis thaliana*	86.9	Q9SSA4_ARATH
		*Oryza sativa* subsp*. Japonica*	84.6	Q6L5F3_ORYSJ
		*Oryza sativa* subsp*. Indica*	84.6	A2Y7U2_ORYSI
		*Zea mays*	83.3	B6U656_MAIZE
*VviMAPKKK56*	GSVIVT01033779001	*Populus trichocarpa*	80.9	B9IFS3_POPTR
		*Prunus salinica*	79.8	A9UAN3_9ROSA
		*Ricinus communis*	79.8	B9SRG7_RICCO
		*Prunus persica*	78.9	C4PKQ3_PRUPE
		*Rosa hybrid cultivar*	78.6	Q93XL9_ROSHC
		*Malus domestica*	78.5	A2T3V2_MALDO
		*Solanum lycopersicum*	77.1	Q5YKK5_SOLLC
*VviMAPKKK58*	GSVIVT01034988001	*Ricinus communis*	78.3	B9RZR2_RICCO
		*Populus trichocarpa*	78.0	B9HIN4_POPTR
*VviMAPKKK61*	GSVIVT01037773001	*Ricinus communis*	80.4	B9SSS7_RICCO
		*Gossypium hirsutum*	79.5	Q7Y236_GOSHI
		*Arabidopsis thaliana*	76.8	WNK11_ARATH
*VvMAP4K3*	GSVIVT01014297001	*Ricinus communis*	76.6	B9T3W4_RICCO
		*Populus trichocarpa*	76.1	B9N1E7_POPTR
*VvMAP4K5*	GSVIVT01019643001	*Populus trichocarpa*	79.8	B9GK86_POPTR
		*Ricinus communis*	79.3	B9RYT1_RICCO
*VvMAP4K7*	GSVIVT01032461001	*Populus trichocarpa*	80.0	B9GHQ7_POPTR
		*Carica papaya*	79.0	A7L4B0_CARPA
		*Arabidopsis thaliana*	75.6	Q9LER4_ARATH

*Columns 1–4 contain the protein name, Vitis proteome 12× ID, GenBank ID, species, percentage identity (%ID), UniprotKB ID*.

All of the 14 *Vitis* MAPK proteins are represented in the Vitis ESTs database (Supplementary Table 1) and are expressed in different tissues such as fruits, berries, buds, flowers, leaves, and roots. In addition, 12 *VvMPK* genes were isolated (Wang et al., 2014a). Expression analysis of *VvMPK* genes showed that all *VvMPK* genes are expressed during grapevine growth and development, and in biotic and abiotic stresses (Wang et al., 2014a).

### MAPKKs

**Figure 3:**
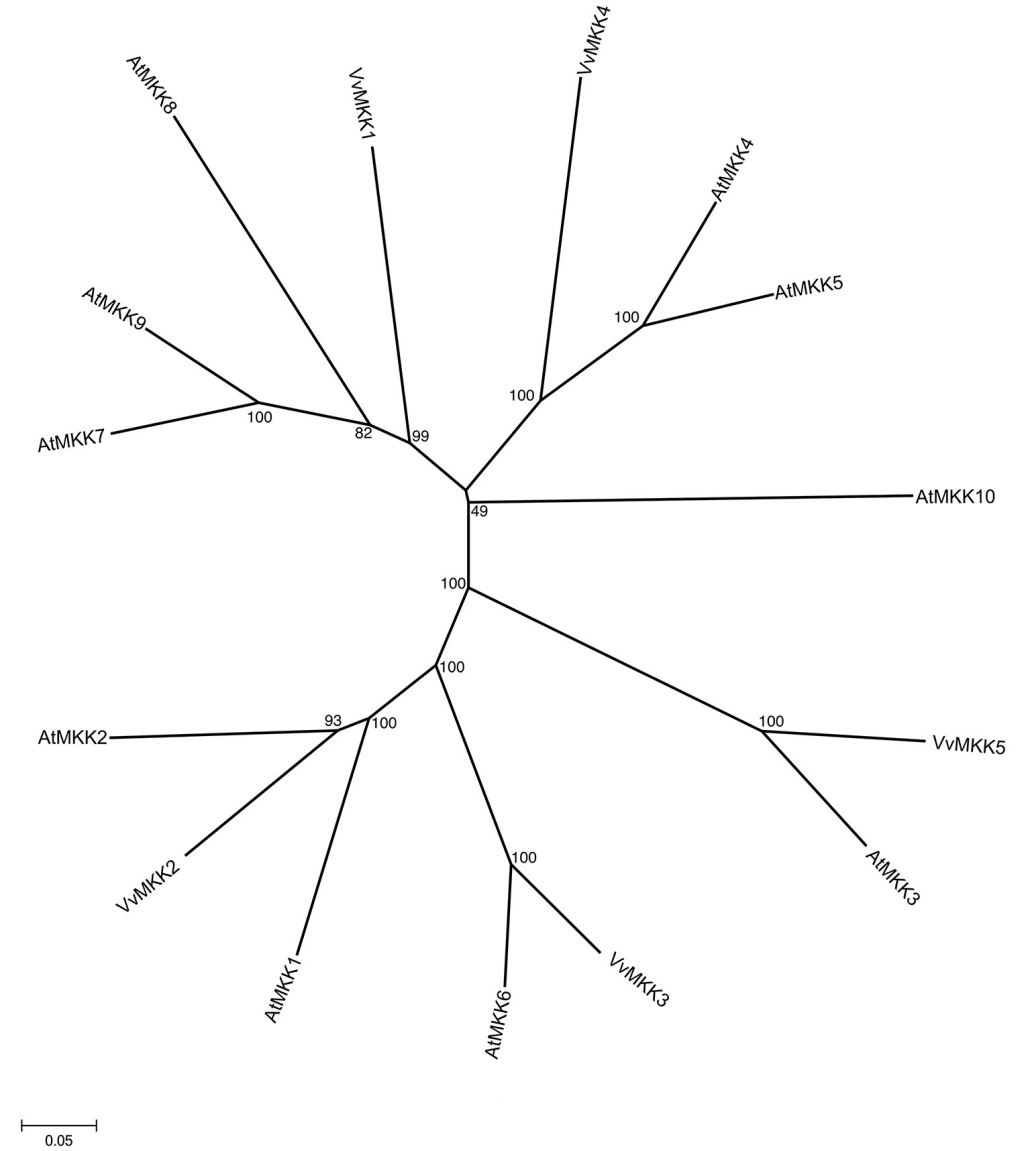
**Phylogenetic tree of MAPKK protein sequences from *Arabidopsis* and *Vitis vinifera***. The amino acid sequences of all *Arabidopsis* MAPKK proteins and those of *Vitis vinifera* were aligned using the MUSCLE program and subjected to phylogenetic analysis by the distance with neighborjoining method using MEGA5 programme. Accession numbers for Arabidopsis sequences are AtMKK1 (At4g26070), AtMKK2 (At4g29810), AtMKK3 (At5g40440), AtMKK4 (At1g51660), AtMKK5 (At3g21220), AtMKK 6 (At5g56580), AtMKK7 (At1g18350), AtMKK8 (At3g06230), AtMKK9 (At1g73500), AtMKK10 (At1g32320).

This subfamily consists of 10 members in *Arabidopsis* genome (Group et al., 2002), whereas *Vitis* genome contains 5 members of MAPKK subfamily. The full length VvMKK sequences range in size from 224 to 519 amino acids (Table 1). The members of the MAPKK subfamily in the *Vitis* genome share 29–40% similarity with each other. By phylogenetic analysis, we also identified orthologs of *Vitis* MAPKKs in *Arabidopsis* such as VvMKK5/AtMKK3 (78.6% similarity), VvMKK3/AtMKK6 (83.1% similarity), and VvMKK2/AtMKK2 (70.4% similarity) supported with significant bootstrap values. The phylogenetic analysis confirmed that VvMKK3 shares 83.3% similarity with its homolog from *Arabidopsis* on the basis of orthology analysis, (Figure 3, Table 2).

To date, none of the *Vitis* MAPKK homologs have been cloned or characterized. However, 98 ESTs were identified for this subfamily in different tissues in response to biotic or abiotic stresses (Supplementary Table 2). A role of MAPK kinase, MKK1 in abiotic stress signaling was previously demonstrated (Matsuoka et al., 2002). Analysis of MKK1 revealed that drought, salt stress, cold, wounding activated MKK1, which in turns activates its downstream target MPK4 (Matsuoka et al., 2002). Tobacco NtMEK2 is functionally interchangeable with two *Arabidopsis* MAPKKs, AtMKK4, and AtMKK5 in activating the downstream MAPKs (Ren et al., 2002). MdMKK1 was reported to be downregulated by ABA (Wang et al., 2010). In *Arabidopsis*, AtMKK3 is upregulated in response to ABA (Hwa and Yang, 2008). Interestingly, AtMKK1/AtMKK2 play an important role in signaling in ROS homeostasis (Liu, 2012).

### MAPKKKs

With 62 members, the MAPKKK subfamily represents the largest subfamily of *V. vinifera* MAPK cascade proteins, which is smaller than those of *Arabidopsis* (80 members) and rice (75 members) (Colcombet and Hirt, 2008; Rao et al., 2010). Recently, Wang et al. (2014b) identified 45 MAPKKK genes in grapevine 12x genome coverage (Wang et al., 2014b). The difference in the number of MAPKKK members in grapevine genome may be related to the “E” value > E-120 used in this report, which is more significant. In addition, domain scan using two different databases (PROSITE and CDD) can identify more sequences in the grapevine genome.

**Figure 4:**
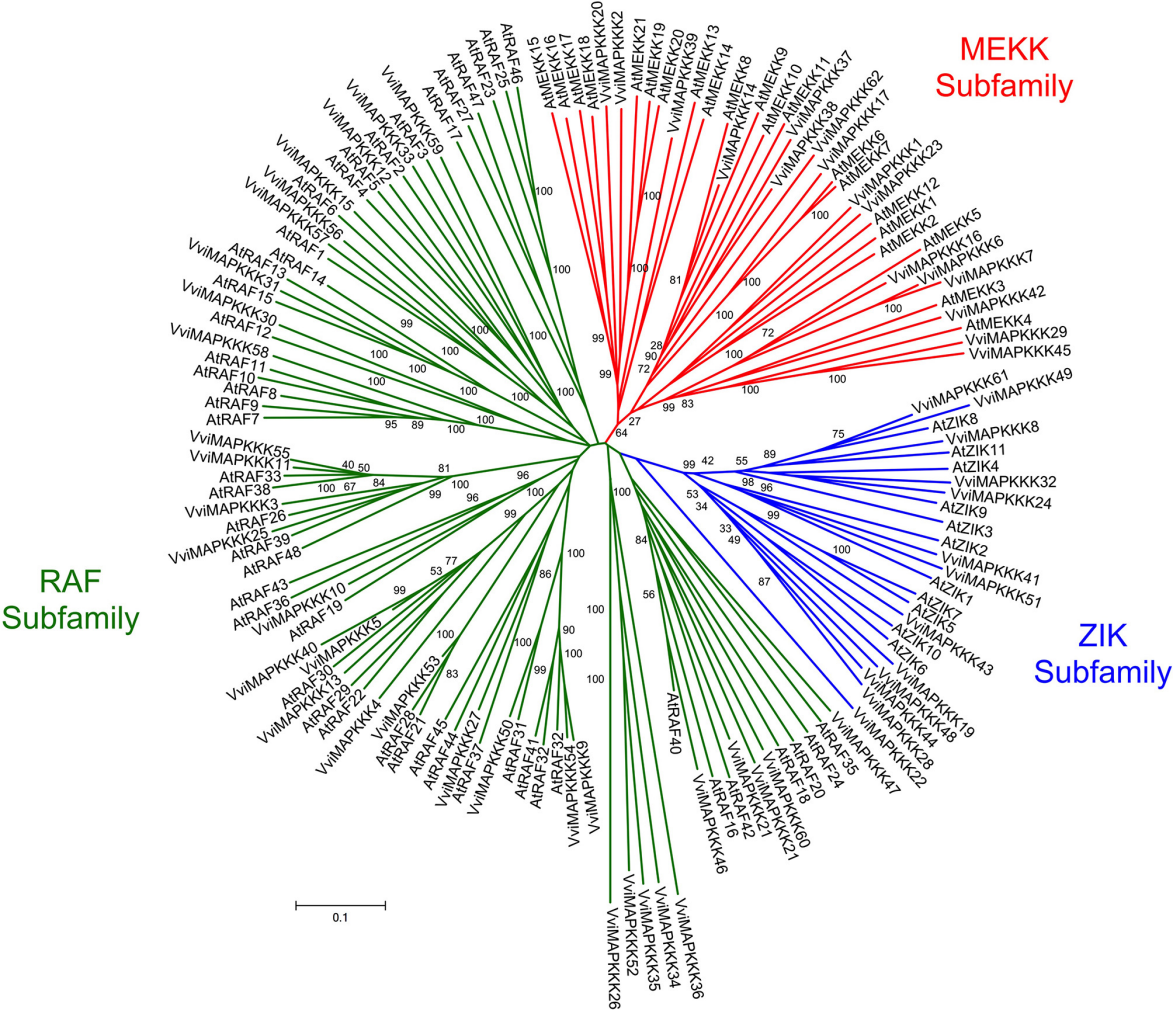
**Phylogenetic tree of MAPKKK protein sequences from *Arabidopsis* and *Vitis vinifera***. The amino acid sequences of all *Arabidopsis* MAPKKK proteins and those of *Vitis vinifera* were aligned using the MUSCLE program and subjected to phylogenetic analysis by the distance with neighborjoining method using MEGA5 programme. MAPKKK forms the largest group of MAPK cascades with 62 members classified into three subfamilies, MEKK, Raf, and ZIK containing 21, 29, and 12 genes, recpectively in *Vitis* genome. Accession numbers for Arabidopsis sequences are AtMEKK1 (At1g09000), AtMEKK2 (At1g54960), AtMEKK3 (At1g53570), AtMEKK4 (At1g63700), AtMEKK5 (At5g66850), AtMEKK6 (At3g07980), AtMEKK7 (At3g13530), AtMEKK8 (At4g08500), AtMEKK9 (At4g08480), AtMEKK10 (At4g08470), AtMEKK11 (At4g12020), AtMEKK12 (At3g06030), AtMEKK13 (At1g07150), AtMEKK14 (At2g30040), AtMEKK15 (At5g55090), AtMEKK16 (At4g26890), AtMEKK17 (At2g32510), AtMEKK18 (At1g05100), AtMEKK19 (At5g67080), AtMEKK20 (At3g50310), AtMEKK21 (At4g36950), AtRAF1 (At5g03730), AtRAF2 (At1g08720), AtRAF3 (At5g11850), AtRAF4 (At1g18160), AtRAF5 (At1g73660), AtRAF6 (At4g24480), AtRAF7 (At3g06620), AtRAF8 (At3g06630), AtRAF9 (At3g06640), AtRAF10 (At5g49470), AtRAF11 (At1g67890), AtRAF12 (At4g23050), AtRAF13 (At2g31010), AtRAF14 (At2g42630), AtRAF15 (At3g58640), AtRAF16 (At1g04700), AtRAF17 (At1g14000), AtRAF18 (At1g16270), AtRAF19 (At1g62400), AtRAF20 (At1g79570), AtRAF21 (At2g17700), AtRAF22 (At2g24360), AtRAF23 (At2g31800), AtRAF24 (At2g35050), AtRAF25 (At2g43850), AtRAF26 (At4g14780), AtRAF27 (At4g18950), AtRAF28 (At4g31170), AtRAF29 (At4g35780), AtRAF30 (At4g38470), AtRAF31 (At5g01850), AtRAF32 (At5g40540), AtRAF33 (At5g50000), AtRAF34 (At5g50180), AtRAF35 (At5g57610), AtRAF36 (At5g58950), AtRAF37 (At5g66710), AtRAF38 (At3g01490), AtRAF39 (At3g22750), AtRAF40 (At3g24720), AtRAF41 (At3g27560), AtRAF42 (At3g46920), AtRAF43 (At3g46930), AtRAF44 (At3g50720), AtRAF45 (At3g50730), AtRAF46 (At3g59830), AtRAF47 (At3g58760), AtRAF48 (At3g63260), AtZIK1 (At3g51630), AtZIK2 (At5g58350), AtZIK3 (At3g22420), AtZIK4 (At3g04910), AtZIK5 (At3g18750), AtZIK6 (At5g41990), AtZIK7 (At1g49160), AtZIK8 (At5g55560), AtZIK9 (At5g28080), AtZIK10 (At1g64630), AtZIK11 (At3g48260).

The members of the *Vitis* MAPKKK subfamily share 11–35% identity with each other and distributed on various chromosomes (from 2 to 18) (Table 1). The full length *Vitis* MAPKKK sequences range from 175 (VviMAPKKK38) to 1397 (VviMAPKKK17) amino acids. The phylogenetic analysis of both *Vitis* and *Arabidopsis* MAPKKK sequences shows that this subfamily is categorized into three main groups with bootstrap values up to 93% (Figure 4).

The first group contains MAPKKKs whose kinase domains have similarity to MEKK subfamily members (Figure 4) (Jonak et al., 2002). A second group includes Raf subfamily members while a third group presents ZIK subfamily members (Figure 4) (Jonak et al., 2002). In total, there are 21 VviMAPKKKs in the MEKK subfamily, while there are 12 in the ZIK subfamily and 29 in the Raf subfamily among the 62 members in the *Vitis* genome.

Analysis of conserved domain of VviMAPKKKs identified a long regulatory domain in the N-terminal region and a kinase domain in the C-terminal region in most of VviMAPKKKs. It is suggested that the long regulatory domain in the N-terminal region of the Raf subfamily may be involved in protein-protein interactions and regulate or specify their kinase activity (Jouannic et al., 1999). Twenty members of the *Vitis* MAPKKK subfamily share 75.1–89.2% similarity with their orthologs from different plant species (Table 2).

We identified at least 640 ESTs for 59 of the *Vitis* MAPKKKs (Supplementary Table 3) indicating that MAPKKK subfamily is transcriptionally active. Expression profile of *VviMAPKKK* genes suggested that some of them are involved in response to biotic and abiotic stresses in different tissues and organs (Wang et al., 2014b). In support of a role for some *Vitis* MAPKKKs, *AtMEKK1* expression is enhanced by drought, salt, stress (Mizoguchi et al., 1996). Recently, it was reported that AtMKK1/MKK2 and AtMEKK1 were able to negatively regulate programmed cell death (PCD) as well as immune responses (Kong et al., 2012). In tobacco, NPK1-MEK1-Ntf6 are also involved in resistance to tobacco mosaic virus (TMV) (Jin et al., 2002; Liu et al., 2004). In addition, AtEDR1, a Raf-like MAPKKK could regulate SA-inducible defense responses negatively (Frye et al., 2001).

### MAPKKKKs

In non-plants, MAPKKKs are activated either through phosphorylation by MAPKKK kinase (MAPKKKK or MAP4K) (Posas and Saito, 1997; Sells et al., 1997) or by G protein and G protein-coupled receptors (Fanger et al., 1997; Sugden and Clerk, 1997).

Several MAP4Ks have been identified in plant genomes based on phylogenetic analyses of their kinase domain. A MAP4K, named MIK, was characterized from the *Zea mays* (Wang et al., 2014d). Recently, a new MAP4K from GCK-II subfamily named ScMAP4K1, which play important roles in ovule, seed, and fruit development was characterized (Major et al., 2009).

**Figure 5:**
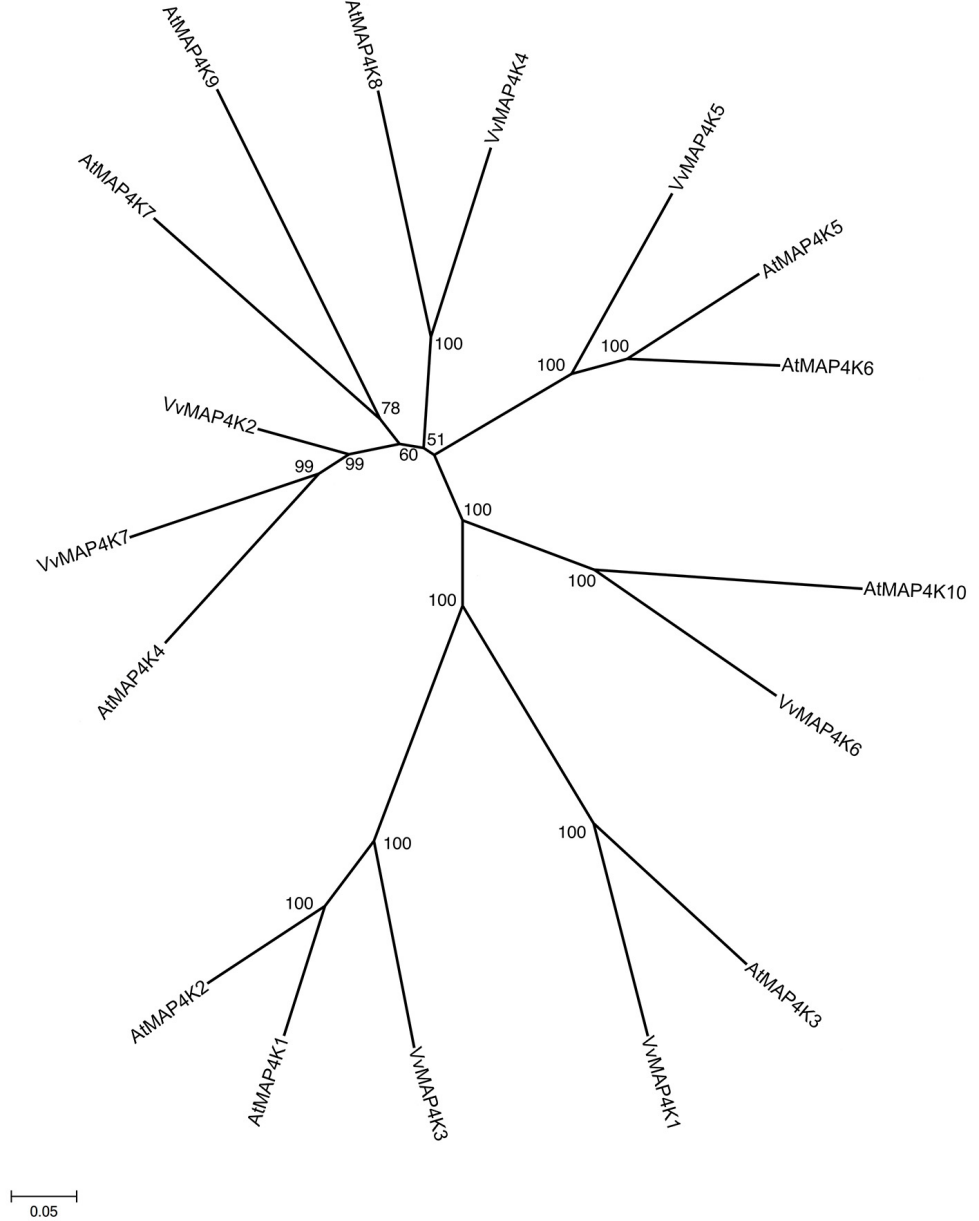
**Phylogenetic tree of MAPKKKK protein sequences from *Arabidopsis* and *Vitis vinifera***. The amino acid sequences of all *Arabidopsis* MAPKKKK proteins and those of *Vitis vinifera* were aligned using the MUSCLE program and subjected to phylogenetic analysis by the distance with neighborjoining method using MEGA5 programme. Accession numbers for Arabidopsis sequences are AtMAP4K1 (At1g53165), AtMAP4K2 (At3g15220), AtMAP4K3 (At1g69220), AtMAP4K4 (At5g14720), AtMAP4K5 (At4g24100), AtMAP4K6 (At4g10730), AtMAP4K7 (At1g70430), AtMAP4K8 (At1g79640), AtMAP4K9 (At1g23700), AtMAP4K10 (At4g14480).

In fully sequenced genomes, like *Arabidopsis* and rice at least 10 protein kinases can be phylogenetically classified as MAP4K (Champion et al., 2004). Little is known about the roles of MAP4Ks in plants. Seven ORFs showing strong similarity with the 10 *Arabidopsis* MAP4Ks were identified in *Vitis* genome (Figure 5) and shared 18–74% similarity with each other. They have been named VvMAP4K1 through 7 (Table 1). The phylogenetic analysis of *V. vinifera* and *A. thaliana* MAP4Ks proteins identified several orthologs in the two species such as VvMAP4K4/AtMAP4K8 (70% similarity), VvMAP4K1/AtMAP4K3 (66% similarity), VvMAP4K7/AtMAP4K4 (68% similarity), and VvMAP4K6/AtMAP4K10 (64% similarity) (Figure 5).

In addition, we identified several orthologs from different species for 3 VvMAP4Ks (Table 2). Among 7 ORFs encoding *Vitis* MAP4Ks, all of them are transcriptionally active (Supplementary Table 4), but none of them has been cloned and characterized.

## Conclusions

This report represents the first complete genome-wide analysis of MAPK cascade proteins in grapevine. The identification of *Vitis* MAPK cascade proteins and their comparative analysis with the *Arabidopsis* MAPK cascade proteins indicates that MAPK cascade genes have been conserved during evolution. In this report, we annotated 90 ORFs encoding MAPK cascade proteins in *V. vinifera* using a bioinformatics approach. Taken as a whole, our data provide significant insights into future biological and physiological analysis of MAPK cascades from *V. vinifera*.

## Author contributions

BÇ conceived and designed all research. OK performed the bioinformatic analyses. BÇ analyzed data and wrote the article.

### Conflict of interest statement

The authors declare that the research was conducted in the absence of any commercial or financial relationships that could be construed as a potential conflict of interest.

## Abbreviations

MAPK: mitogen-activated protein kinase
ORF: open reading frame.
