# Supplementary material for: Mitogen-activated protein kinase cascades in Vitis vinifera
Source: Front Plant Sci. 2015 Jul 22;6:556. doi: 10.3389/fpls.2015.00556 (PMC4511077; doi:10.3389/fpls.2015.00556)
Supplement: Supplementary file 1 [file Table1.DOC]

**Supplementary Table 1.** **Expressed sequence tags (ESTs) identified for the MAPK subfamily in *Vitis vinifera*.** The protein name, *Vitis* proteome 12× ID, GenBank ID, EST name, cultivar/tissue type, and development stage are given for each gene.

| **Name** | ***Vitis* 12X ID** | **EST Name** | **GenBank ID** | **Species/Cultivar** | **Tissue Type** | **Development Stage** |
| --- | --- | --- | --- | --- | --- | --- |
| *VvMPK1* | GSVIVT01000784001 | C2B07288 | 110690752 | Carmenere | Bud - cluster |  |
|  |  | CAP0005_IIR_D05 | 34549100 | Cabernet Sauvignon | Petiole | Onset of Veraison (berry softening) |
|  |  | CAP0005_IIF_D05 | 34549623 | Cabernet Sauvignon | Petiole | Onset of Veraison (berry softening) |
|  |  | VVH006B10_739209 | 71864828 | Cabernet Sauvignon | Nectary of flowers | 25 - modified E-L system |
|  |  | SCB05271 | 110730439 | Thompson-seedless | Inflorescence |  |
|  |  | S2B11969 | 110701446 | Thompson-seedless | Bud |  |
|  |  | S2B12162 | 110701964 | Thompson-seedless | Bud |  |
|  |  | WIN064.C21_F06 | 110392523 | Cabernet Sauvignon | Seed | Fruit set to maturity |
|  |  | CSECS155A09_PREn0028 | 51052283 | Cabernet Sauvignon | Fruit | 28 - modified E-L system |
|  |  | CA12EI203IIR_G04 | 26266316 | Cabernet Sauvignon | Leaf | Mid-season leaf material |
| *VvMPK2* | GSVIVT01005924001 | S9B07491 | 110724486 | Thompson-seedless | Berry |  |
|  |  | sT7aVVM_AER79C10 | 161711995 | Cabernet Sauvignon | Root | 10 cm high plants grown in Magenta boxes |
|  |  | SCB01016 | 110729354 | Thompson-seedless | Inflorescence |  |
|  |  | VVD139E03_374019 | 30126725 | Chardonnay | Berries | Mixed; 8, 9, 11, 13, 15, 16 weeks daf |
|  |  | VV_PEb14a12.g1 | 156730306 | Perlette | Bud | Mature |
|  |  | VV_PEb14a12.b1 | 156730305 | Perlette | Bud | Mature |
|  |  | WIN1016.C21_G24 | 110407797 | Muscat Hamburg | Pericarp | Fruit set to maturity |
|  |  | VVA006F08_52761 | 18458311 | Chardonnay | Leaf | Juvenile and adult |
|  |  | VV_PEd0011g05.g1 | 156736159 | Perlette | Bud | Young |
|  |  | sT7aVVM003K20070 | 161712737 | Cabernet Sauvignon | Roots | 10 cm high plants grown in Magenta boxes |
|  |  | CAB20001_IIa_Fa_A11 | 33401585 | Cabernet Sauvignon | Flower - Bloom | Bloom |
|  |  | VVA006F08_390855 | 30320369 | Chardonnay | Leaf | Juvenile and adult |
|  |  | INFIO01_000460 | 37190302 | Regent | Inflorescence | Young inflorescence before flowering |
|  |  | CAB20001_IIa_Ra_A11 | 33401665 | Cabernet Sauvignon | Flower - Bloom | Bloom |
|  |  | VV_PEd0011g05.b1 | 156736158 | Perlette | Bud | Young |
|  |  | WIN1138.C21_D22 | 110423640 | Muscat Hamburg | Berry | Anthesis flower to prior to veraison |
|  |  | CSECS057A03_VERu0035 | 34364063 | Cabernet Sauvignon | Fruit with seeds removed | 35 - modified E-L system |
|  |  | VVA006F08_392105 | 30320994 | Chardonnay | Leaf | Juvenile and adult |
| *VvMPK3* | GSVIVT01008408001 | GEMMA01_001537 | 37188656 | Pinot Noir | Bud | Bud swelling |
|  |  | sT7aVVM009C17077 | 161714277 | Cabernet Sauvignon | Roots | 10 cm high plants grown in Magenta boxes |
|  |  | VV_PEa19a01.g1 | 156727001 | Perlette | Bud | Mature |
|  |  | VVL090B01_689976 | 71885120 | Cabernet Sauvignon | Fruit with seeds removed | Mixed 36-38 - modified E-L system (Brix > 15) |
|  |  | sT7aVVM019O21081 | 161716597 | Cabernet Sauvignon | Roots | 10 cm high plants grown in Magenta boxes |
|  |  | sT7aVVM_AER60B01 | 161711142 | Cabernet Sauvignon | Roots | 11 cm high plants grown in Magenta boxes |
|  |  | VVH038B12_744959 | 71860512 | Cabernet Sauvignon | Nectary of flowers | 25 - modified E-L system |
|  |  | CAP0005_IIF_D05 | 34549623 | Cabernet Sauvignon | Petiole | Onset of Veraison (berry softening) |
| *VvMPK4* | GSVIVT01009766001 | CAP0004_IVF_E12 | 34549382 | Cabernet Sauvignon | Petiole | Onset of Veraison (berry softening) |
|  |  | CAB20002_Ia_Ra_F02 | 33402439 | Cabernet Sauvignon | Flower - Bloom | Bloom |
|  |  | CAB2SG0006_IIIbF_H09 | 29783201 | Cabernet Sauvignon | Berry | Veraison |
|  |  | WIN0537.C21_P20 | 110380892 | Cabernet Sauvignon | Flower, leaf and root | Flower, pre-anthesis; leaf, fully expanded; root, produced by air-layering |
|  |  | sT7aVVM_AER34G05 | 161707145 | Cabernet Sauvignon | Roots | 10 cm high plants grown in Magenta boxes |
|  |  | CAB20002_Ib_Fb_F02 | 33402523 | Cabernet Sauvignon | Flower - Bloom | Bloom |
|  |  | WIN0828.C21_K14 | 110401795 | Cabernet Sauvignon | Seed | Fruit set to maturity |
|  |  | sT7aVVM024B06031 | 161717333 | Cabernet Sauvignon | Roots | 10 cm high plants grown in Magenta boxes |
|  |  | AMU_USDA_FP_3888 | 51578029 | Vitis shuttleworthii | Entire tendril, leaves, bud, flowers | At blooming |
|  |  | WIN0513.C21_N13 | 110372708 | Cabernet Sauvignon | Flower, leaf and root | Flower, pre-anthesis; leaf, fully expanded; root, produced by air-layering |
|  |  | VVL135G07_697898 | 71889081 | Cabernet Sauvignon | Fruit with seeds removed | Mixed 36-38 - modified E-L system (Brix > 15) |
|  |  | WIN0214.TB24_B23 | 110363382 | Cabernet Sauvignon | Flower, leaf and root | Flower, pre-anthesis; leaf, fully expanded; root, produced by air-layering |
|  |  | VVG018A12_754037 | 71855506 | Cabernet Sauvignon | Cell Suspension Culture |  |
|  |  | CAB20002_Ia_Ra_D02 | 33402416 | Cabernet Sauvignon | Flower - Bloom | Bloom |
|  |  | CAB20002_Ib_Fb_D02 | 33402501 | Cabernet Sauvignon | Flower - Bloom | Bloom |
|  |  | CAP0004_IVR_E12 | 34549468 | Cabernet Sauvignon | Petiole | Onset of Veraison (berry softening) |
|  |  | CAST0003_IR_D12 | 33410434 | Cabernet Sauvignon | Stem | Pre-bloom (10-11 days before bloom) |
|  |  | S2B24451 | 110702545 | Thompson-seedless | Bud |  |
|  |  | WIN0517.C21_I02 | 110374792 | Cabernet Sauvignon | Flower, leaf and root | Flower, pre-anthesis; leaf, fully expanded; root, produced by air-layering |
| *VvMPK5* | GSVIVT01011749001 | EST 14281 | 32456726 | Chardonnay | Fruit pedicle | Ripe stage |
|  |  | C2B07288 | 110690752 | Carmenere | Bud - cluster |  |
|  |  | sT7aVVM019O21081 | 161716597 | Cabernet Sauvignon | Roots | 10 cm high plants grown in Magenta boxes |
|  |  | VVL090B01_689976 | 71885120 | Cabernet Sauvignon | Fruit with seeds removed | Mixed 36-38 - modified E-L system (Brix > 15) |
| *VvMPK6* | GSVIVT01014081001 | VVH013E12_740565 | 71861898 | Cabernet Sauvignon | Nectary of flowers | 25 - modified E-L system |
|  |  | CA12EI203IIR_G04 | 26266316 | Cabernet Sauvignon | Leaf | Mid-season leaf material |
|  |  | VVH038B12_744959 | 71860512 | Cabernet Sauvignon | Nectary of flowers | 25 - modified E-L system |
|  |  | sT7aVVM_AER27E04 | 161711847 | Cabernet Sauvignon | Roots | 10 cm high plants grown in Magenta boxes |
|  |  | CAbud0005_IF_D11 | 34544640 | Cabernet Sauvignon | Bud | Pre-bloom (10-11 days before bloom) |
|  |  | VVG055D02_761715 | 71859345 | Cabernet Sauvignon | Cell Suspension Culture |  |
|  |  | WIN029.TB24_E02 | 110362051 | Cabernet Sauvignon | Flower, leaf and root | Flower, pre-anthesis; leaf, fully expanded; root, produced by air-layering |
|  |  | sT7aVVM_AER11H10 | 161710988 | Cabernet Sauvignon | Roots | 10 cm high plants grown in Magenta boxes |
|  |  | WIN0530.C21_K15 | 110378888 | Cabernet Sauvignon | Flower, leaf and root | Flower, pre-anthesis; leaf, fully expanded; root, produced by air-layering |
|  |  | VVH006B10_739209 | 71864828 | Cabernet Sauvignon | Nectary of flowers | 25 - modified E-L system |
|  |  | AB20007_IIIa_Fa_F09 | 33405289 | Cabernet Sauvignon | Flower - Bloom | Bloom |
|  |  | 9_67_Sh_TC_P5_E03.ab1 1 372 | 134031334 | Vitis arizonica x Vitis rupestris | Shoot | Vegetative stage infected with Xylella fastidiosa |
|  |  | SCB06463 | 110730938 | Thompson-seedless | Inflorescence |  |
|  |  | SECS143G06_NECu0025 | 45771272 | Cabernet Sauvignon | Nectary of flowers | 25 - modified E-L system |
|  |  | CAB20007_IIIa_Ra_F09 | 33405374 | Cabernet Sauvignon | Flower - Bloom | Bloom |
|  |  | V-B-113C09 | 28602876 | Vitis aestivalis/Norton | Leaf | Young leaf |
|  |  | V-B-113B07 | 28602863 | Vitis aestivalis/Norton | Leaf | Young leaf |
|  |  | S5B02559 | 110710600 | Thompson-seedless | Fruit |  |
|  |  | EST 16184 | 46919217 | Chardonnay | Fruit pedicle | Green stage |
|  |  | WIN0522.C21_F20 | 110376276 | Cabernet Sauvignon | Flower, leaf and root | Flower, pre-anthesis; leaf, fully expanded; root, produced by air-layering |
|  |  | VVH037C08_744799 | 71860432 | Cabernet Sauvignon | Nectary of flowers | 25 - modified E-L system |
|  |  | WIN1148.C21_M18 | 110427148 | Muscat Hamburg | Berry | Anthesis flower to prior to veraison |
|  |  | WIN0415.C21_O02 | 110368311 | Cabernet Sauvignon | Pericarp | Fruit set to maturity |
| *VvMPK7* | GSVIVT01017873001 | WIN055.C21_J01 | 110373760 | Cabernet Sauvignon | Flower, leaf and root | Flower, pre-anthesis; leaf, fully expanded; root, produced by air-layering |
|  |  | sT7aVVM018K18069 | 161719505 | Cabernet Sauvignon | Roots | 10 cm high plants grown in Magenta boxes |
|  |  | VVH006B10_739209 | 71864828 | Cabernet Sauvignon | Nectary of flowers | 25 - modified E-L system |
|  |  | WIN058.C21_L18 | 110374411 | Cabernet Sauvignon | Flower, leaf and root | Flower, pre-anthesis; leaf, fully expanded; root, produced by air-layering |
|  |  | WIN0556.C21_E07 | 110387911 | Cabernet Sauvignon | Flower, leaf and root | Flower, pre-anthesis; leaf, fully expanded; root, produced by air-layering |
|  |  | VVH038B12_744959 | 71860512 | Cabernet Sauvignon | Nectary of flowers | 25 - modified E-L system |
|  |  | VVB138C04_406601 | 32268188 | Chardonnay | Leaf | Juvenile and adult |
|  |  | VVG055D02_761715 | 71859345 | Cabernet Sauvignon | Cell Suspension Culture |  |
|  |  | FAMU_USDA_FP_7069 | 51581210 | Vitis shuttleworthii | Mixed population | Entire tendril, leaves, bud, flowers |
|  |  | WIN0553.C21_E19 | 110387017 | Cabernet Sauvignon | Flower, leaf and root | Flower, pre-anthesis; leaf, fully expanded; root, produced by air-layering |
|  |  | CA12EI203IIR_G04 | 26266316 | Cabernet Sauvignon | Leaf | Mid-season leaf material |
|  |  | VVH027D10_743037 | 71863134 | Cabernet Sauvignon | Nectary of flowers | 25 - modified E-L system |
|  |  | WIN0558.C21_F11 | 110388554 | Cabernet Sauvignon | Flower, leaf and root | Flower, pre-anthesis; leaf, fully expanded; root, produced by air-layering |
|  |  | GEMMA01_001423 | 37188545 | Pinot Noir | Bud | Bud swelling |
|  |  | WIN0817.C21_J01 | 110398766 | Cabernet Sauvignon | Seed | Fruit set to maturity |
|  |  | WIN0415.C21_K07 | 110368230 | Cabernet Sauvignon | Pericarp | Fruit set to maturity |
|  |  | VV_PEd03g08.g1 | 156737893 | Perlette | Bud | Young |
|  |  | VV_PEd03g08.b1 | 156737884 | Perlette | Bud | Young |
|  |  | S2B23967 | 110703575 | Thompson-seedless | Bud |  |
|  |  | WIN019.C21_L18 | 110358818 | Cabernet Sauvignon | Pericarp | Fruit set to maturity |
|  |  | WIN0534.C21_A19 | 110379955 | Cabernet Sauvignon | Flower, leaf and root | Flower, pre-anthesis; leaf, fully expanded; root, produced by air-layering |
|  |  | VV_PEd0008a02.b1 | 156735425 | Perlette | Bud | Young |
|  |  | C3B06170 | 110694163 | Carmenere | Clusters | Clusters 4 cm |
|  |  | CAB20006_IIIa_Ra_G01 | 33404702 | Cabernet Sauvignon | Flower - Bloom | Bloom |
|  |  | sT7aVVM_AER66F10 | 161706097 | Cabernet Sauvignon | Roots | 10 cm high plants grown in Magenta boxes |
|  |  | CGF1000794_H03 | 33407978 | Cabernet Sauvignon | Stem | Pre-bloom (10-11 days before bloom) |
|  |  | VVI010G01_584464 | 71868257 | Cabernet Sauvignon | Inflorescence including flowers | 12 - modified E-L system |
|  |  | INFIO01_000603 | 37190437 | Regent | Inflorescence | Young inflorescence before flowering |
|  |  | CAB20006_IIIa_Fa_G01 | 33404616 | Cabernet Sauvignon | Flower - Bloom | Bloom |
|  |  | CSECS013E09_PREu0032 | 34362218 | Cabernet Sauvignon | Fruit with seeds removed | 32 - modified E-L system |
|  |  | VV_PEd0008a02.g1 | 156735426 | Perlette | Bud | Young |
|  |  | WIN035.C21_J21 | 110365503 | Cabernet Sauvignon | Seed | Fruit set to maturity |
|  |  | S8B00839 | 110719455 | Thompson-seedless | Fruit |  |
|  |  | SCB05271 | 110730439 | Thompson-seedless | Inflorescence |  |
|  |  | C2B07288 | 110690752 | Carmenere | Bud - cluster |  |
| *VvMPK8* | GSVIVT01018883001 | VVG018A12_754037 | 71855506 | Cabernet Sauvignon | Cell Suspension Culture |  |
|  |  | WIN0212.TB24_O08 | 110363057 | Cabernet Sauvignon | Flower, leaf and root | Flower, pre-anthesis; leaf, fully expanded; root, produced by air-layering |
|  |  | sT7aVVM027B16064 | 161720611 | Cabernet Sauvignon | Roots | 10 cm high plants grown in Magenta boxes |
|  |  | VVG027C10_756505 | 71856740 | Cabernet Sauvignon | Cell Suspension Culture |  |
|  |  | VVC027E04_138008 | 27583224 | Chardonnay | Berries | Mixed; 8, 9, 11, 13, 15, 16 weeks daf |
|  |  | sT7aVVM024B06031 | 161717333 | Cabernet Sauvignon | Roots | 10 cm high plants grown in Magenta boxes |
|  |  | S4B04459 | 110705998 | Thompson-seedless | Fruit | Fruits 2-3 mm |
|  |  | EST 12465 | 32456087 | Ugni Blanc | Fruit without seeds | Veraison stage |
|  |  | sT7aVVM018F11044 | 161718388 | Cabernet Sauvignon | Roots | 10 cm high plants grown in Magenta boxes |
|  |  | VVG045D04_759891 | 71858433 | Cabernet Sauvignon | Cell Suspension Culture |  |
|  |  | VVC020D04_416705 | 32246000 | Chardonnay | Berries | Mixed; 8, 9, 11, 13, 15, 16 weeks daf |
|  |  | VVC063F04_230552 | 27586380 | Chardonnay | Berries | Mixed; 8, 9, 11, 13, 15, 16 weeks daf |
|  |  | sT7aVVM_AER36H02 | 161707128 | Cabernet Sauvignon | Roots | 10 cm high plants grown in Magenta boxes |
|  |  | CGF1000810_E02 | 33408294 | Cabernet Sauvignon | Stem | Pre-bloom (10-11 days before bloom) |
|  |  | VVG036G08_758277 | 71857626 | Cabernet Sauvignon | Cell Suspension Culture |  |
|  |  | USDA_FP_131085 | 47089729 | Vitis shuttleworthii | Entire tendril, leaves, bud, flowers | At blooming |
|  |  | FAMU_USDA_FP_5458 | 51579599 | Vitis shuttleworthii | Entire tendril, leaves, bud, flowers | At blooming |
|  |  | FAMU_USDA_FP_00151 | 34995012 | Vitis shuttleworthii | Entire tendril, leaves, bud, flowers | At blooming |
|  |  | S5B03993 | 110709562 | Thompson-seedless | Fruit | Fruits 7-9 mm treated with GA3 |
|  |  | CSECS056A12_VERu0035 | 34364006 | Cabernet Sauvignon | Fruit with seeds removed | 35 - modified E-L system |
|  |  | SCB07257 | 110732789 | Thompson-seedless | Inflorescence | Inflorescence with GA3 |
|  |  | C4B03903 | 110695564 | Carmenere | Clusters | Veraison |
|  |  | EST 6795 | 22012823 | Shiraz | Fruit | Ripening stage |
|  |  | WIN086.C21_K18 | 110397909 | Cabernet Sauvignon | Seed | Fruit set to maturity |
|  |  | WIN0536.C21_C01 | 110384176 | Cabernet Sauvignon | Flower, leaf and root | Flower, pre-anthesis; leaf, fully expanded; root, produced by air-layering |
|  |  | WIN0533.C21_M01 | 110379864 | Cabernet Sauvignon | Flower, leaf and root | Flower, pre-anthesis; leaf, fully expanded; root, produced by air-layering |
| *VvMPK9* | GSVIVT01019406001 | EST 1628 | 22014425 | Shiraz | Fruit | Green stage |
|  |  | VVD059E04_350867 | 30134779 | Chardonnay | Berries | Mixed; 8, 9, 11, 13, 15, 16 weeks daf |
|  |  | CA12LIO2IVF_F08 | 26257224 | Cabernet Sauvignon | Leaf | Late season sample |
|  |  | VV_PEb16h01.g1 | 156730754 | Perlette | Bud | Mature |
|  |  | VVL135G07_697898 | 71889081 | Cabernet Sauvignon | Fruit with seeds removed | Mixed 36-38 - modified E-L system (Brix > 15) |
|  |  | VVC005B08_119196 | 27582055 | Chardonnay | Berries | Mixed; 8, 9, 11, 13, 15, 16 weeks daf |
|  |  | VVD114A02_371207 | 30131434 | Chardonnay | Berries | Mixed; 8, 9, 11, 13, 15, 16 weeks daf |
|  |  | VVL087B06_689454 | 71884859 | Cabernet Sauvignon | Fruit with seeds removed | Mixed 36-38 - modified E-L system (Brix > 15) |
|  |  | SBB03081 | 110727011 | Thompson-seedless | Inflorescence |  |
|  |  | VVC044E01_141774 | 27584664 | Chardonnay | Berries | Mixed; 8, 9, 11, 13, 15, 16 weeks daf |
|  |  | S8B04526 | 110719090 | Thompson-seedless | Fruit | Veraison |
|  |  | R890915I0006_IVa_Ra_G04 | 33400743 | Vitis hybrid cultivar | Leaf |  |
|  |  | WIN0417.C21_K16 | 110369082 | Cabernet Sauvignon | Pericarp | Fruit set to maturity |
|  |  | VVC063F04_416207 | 32245751 | Chardonnay | Berries | Mixed; 8, 9, 11, 13, 15, 16 weeks daf |
|  |  | CAB20002_Ib_Fb_D02 | 33402501 | Cabernet Sauvignon | Flower - Bloom | Bloom |
|  |  | FAMU_USDA_FP_5025 | 51579166 | Vitis shuttleworthii | Entire tendril, leaves, bud, flowers | At blooming |
|  |  | VVC063F04_417465 | 32246380 | Chardonnay | Berries | Mixed; 8, 9, 11, 13, 15, 16 weeks daf |
|  |  | VRK77T7 | 47061152 | Vitis riparia | Bud | Paradormant |
|  |  | VVG032B10_757423 | 71857199 | Cabernet Sauvignon | Cell Suspension Culture |  |
|  |  | FAMU_USDA_FP_861 | 51575002 | Vitis shuttleworthii | Entire tendril, leaves, bud, flowers | At blooming |
|  |  | VVC063F04_230552 | 27586380 | Chardonnay | Berries | Mixed; 8, 9, 11, 13, 15, 16 weeks daf |
|  |  | WIN051.C21_H01 | 110381451 | Cabernet Sauvignon | Flower, leaf and root | Flower, pre-anthesis; leaf, fully expanded; root, produced by air-layering |
| *VvMPK10* | GSVIVT01022771001 | S9B01267 | 110721968 | Thompson-seedless | Berries | Ripening Berries |
|  |  | CAB40001_IVa_Fa_G02 | 30299336 | Cabernet Sauvignon | Berry | Berry on stage II, 9 mm |
|  |  | S9B02615 | 110722338 | Thompson-seedless | Berries | Ripening Berries |
|  |  | WIN072.C21_A05 | 110393383 | Cabernet Sauvignon | Pericarp | Fruit set to maturity |
|  |  | sT7aVVM016L06021 | 161720107 | Cabernet Sauvignon | Roots | 10 cm high plants grown in Magenta boxes |
|  |  | CAbud0002_IVR_E12 | 34543467 | Cabernet Sauvignon | Bud | Pre-bloom (10-11 days before bloom) |
|  |  | S6B04142 | 110713325 | Thompson-seedless | Fruit | Fruits 7-9 mm |
|  |  | WIN0516.C21_M21 | 110373610 | Cabernet Sauvignon | Flower, leaf and root | Flower, pre-anthesis; leaf, fully expanded; root, produced by air-layering |
|  |  | CAB40001_IVa_Ra_G02 | 30299414 | Cabernet Sauvignon | Berry | Berry on stage II, 9 mm |
|  |  | FAMU_USDA_FP_223 | 51574364 | Vitis shuttleworthii | Entire tendril, leaves, bud, flowers | At blooming |
|  |  | CSECS061F09_VERu0035 | 34364363 | Cabernet Sauvignon | Fruit with seeds removed | 35 - modified E-L system |
|  |  | WIN0513.C21_C07 | 110372491 | Cabernet Sauvignon | Flower, leaf and root | Flower, pre-anthesis; leaf, fully expanded; root, produced by air-layering |
|  |  | S9B09173 | 110725023 | Thompson-seedless | Berries | Ripening Berries |
|  |  | CSECS014G08_PREu0032 | 34362325 | Cabernet Sauvignon | Fruit with seeds removed | 35 - modified E-L system |
|  |  | FAMU_USDA_FP_3817 | 51577958 | Vitis shuttleworthii | Entire tendril, leaves, bud, flowers | At blooming |
|  |  | GERMO01_000178 | 37189155 | Regent | Shoot tip | Growing shoot tip (ca. 1 cm long) |
|  |  | WIN0813.C21_H10 | 110396398 | Cabernet Sauvignon | Seed | Fruit set to maturity |
|  |  | CAB20006_IIIa_Fa_G05 | 33404620 | Cabernet Sauvignon | Flower - Bloom | Bloom |
|  |  | WIN1030.C21_O21 | 110410602 | Muscat Hamburg | Pericarp | Fruit set to maturity |
|  |  | SCB00759 | 110731364 | Thompson-seedless | Inflorescence | Inflorescence with GA3 |
|  |  | WIN0813.C21_K11 | 110396457 | Cabernet Sauvignon | Seed | Fruit set to maturity |
|  |  | WIN047.C21_M10 | 110366174 | Cabernet Sauvignon | Seed | Fruit set to maturity |
|  |  | WIN089.C21_L19 | 110398533 | Cabernet Sauvignon | Seed | Fruit set to maturity |
|  |  | WIN088.C21_D01 | 110403163 | Cabernet Sauvignon | Seed | Fruit set to maturity |
|  |  | WIN0814.C21_J14 | 110396778 | Cabernet Sauvignon | Seed | Fruit set to maturity |
|  |  | WIN063.C21_G19 | 110392340 | Cabernet Sauvignon | Seed | Fruit set to maturity |
|  |  | WIN0538.C21_N09 | 110381167 | Cabernet Sauvignon | Flower, leaf and root | Flower, pre-anthesis; leaf, fully expanded; root, produced by air-layering |
|  |  | WIN048.C21_O22 | 110366390 | Cabernet Sauvignon | Seed | Fruit set to maturity |
|  |  | WIN107.C21_N13 | 110412692 | Cabernet Sauvignon | Pericarp | Fruit set to maturity |
|  |  | WIN1029.C21_A01 | 110410017 | Muscat Hamburg | Pericarp | Fruit set to maturity |
|  |  | CAbud0002_IIIF_E12 | 34543553 | Cabernet Sauvignon | Bud | Pre-bloom (10-11 days before bloom) |
|  |  | EST 3131 | 22009159 | Shiraz | Fruit | Veraison |
|  |  | EST 5106 | 22011134 | Shiraz | Fruit | Ripening stage |
|  |  | CSECS121F02_PREn0028 | 51051168 | Cabernet Sauvignon | Fruit | 28 - modified E-L system |
|  |  | WIN084.C21_K23 | 110395179 | Cabernet Sauvignon | Seed | Fruit set to maturity |
|  |  | WIN0527.C21_B03 | 110377773 | Cabernet Sauvignon | Flower, leaf and root | Flower, pre-anthesis; leaf, fully expanded; root, produced by air-layering |
|  |  | CAB70005_IIIaF_F09 | 30304869 | Cabernet Sauvignon | Berry | Post-Veraison, 18-19 brix |
|  |  | WIN0529.C21_D23 | 110378432 | Cabernet Sauvignon | Flower, leaf and root | Flower, pre-anthesis; leaf, fully expanded; root, produced by air-layering |
|  |  | WIN1011.C21_L08 | 110406438 | Muscat Hamburg | Pericarp | Fruit set to maturity |
|  |  | VVC048C01_142430 | 27584992 | Chardonnay | Berries | Mixed; 8, 9, 11, 13, 15, 16 weeks daf |
|  |  | WIN0417.C21_D02 | 110368928 | Cabernet Sauvignon | Pericarp | Fruit set to maturity |
|  |  | WIN1142.C21_B06 | 110424903 | Muscat Hamburg | Berry | Anthesis flower to prior to veraison |
|  |  | WIN094.C21_J09 | 110404677 | Cabernet Sauvignon | Pericarp | Fruit set to maturity |
|  |  | S7B04739 | 110717334 | Thompson-seedless | Berry | Berries 14mm with GA3 |
|  |  | VVD010D01_343721 | 30128894 | Chardonnay | Berries | Mixed; 8, 9, 11, 13, 15, 16 weeks daf |
|  |  | WIN1123.C21_N07 | 110418911 | Muscat Hamburg | Berry | Anthesis flower to prior to veraison |
|  |  | WIN1130.C21_D18 | 110421000 | Muscat Hamburg | Berry | Anthesis flower to prior to veraison |
|  |  | WIN115.C21_G09 | 110427663 | Muscat Hamburg | Berry | Anthesis flower to prior to veraison |
|  |  | VVA021G04_402721 | 32245277 | Chardonnay | Leaf | Juvenile and adult |
|  |  | VVA021G04_54659 | 18459304 | Chardonnay | Leaf | Juvenile and adult |
| *VvMPK11* | GSVIVT01025091001 | C3B05231 | 110692105 | Carmenere | Clusters | Clusters 4 cm |
|  |  | CGF1000661_B09 | 33406469 | Cabernet Sauvignon | Stem | Pre-bloom (10-11 days before bloom) |
|  |  | VVB138C04_406601 | 32268188 | Chardonnay | Leaf | Juvenile and adult |
|  |  | EST 18402 | 46911256 | Cabernet Sauvignon | Fruit without seeds | Veraison stage |
|  |  | C1G08048 | 110686022 | Carmenere | Fruit bud cluster |  |
|  |  | VVL066H04_686078 | 71883171 | Cabernet Sauvignon | Fruit with seeds removed | Mixed 36-38 - modified E-L system (Brix > 15) |
|  |  | CA48LN07IVF-B4 | 26261816 | Cabernet Sauvignon | Leaf | Late season sample |
|  |  | V-B-126F03 | 33962082 | Vitis aestivalis/Norton | Leaf | Young leaf |
|  |  | CAB20006_IIIa_Ra_B12 | 33404655 | Cabernet Sauvignon | Flower - Bloom | Bloom |
|  |  | VVL050H07_683296 | 71881780 | Cabernet Sauvignon | Fruit with seeds removed | Mixed 36-38 - modified E-L system (Brix > 15) |
|  |  | FAMU_USDA_FP_5025 | 51579166 | Vitis shuttleworthii | Entire tendril, leaves, bud, flowers | At blooming |
|  |  | CAbud0003_IIIR_D10 | 34544291 | Cabernet Sauvignon | Bud | Pre-bloom (10-11 days before bloom) |
|  |  | VVG032B10_757423 | 71857199 | Cabernet Sauvignon | Cell Suspension Culture |  |
|  |  | VVD059E04_350867 | 30134779 | Chardonnay | Berries | Mixed; 8, 9, 11, 13, 15, 16 weeks daf |
|  |  | WIN1125.C21_O11 | 110419567 | Muscat Hamburg | Berry | Anthesis flower to prior to veraison |
|  |  | EST 18498 | 46911352 | Cabernet Sauvignon | Fruit without seeds | Veraison stage |
| *VvMPK12* | GSVIVT01025105001 | EST 6045 | 22012073 | Shiraz | Fruit | Ripening stage |
|  |  | VVD027H11_346741 | 30132715 | Chardonnay | Berries | Mixed; 8, 9, 11, 13, 15, 16 weeks daf |
|  |  | sT7aVVM_AER20B06 | 161707844 | Cabernet Sauvignon | Roots | 10 cm high plants grown in Magenta boxes |
|  |  | CSECS140G01_CELu0001 | 45771046 | Cabernet Sauvignon | Cell Suspension Culture |  |
|  |  | sT7aVVM009C17077 | 161714277 | Cabernet Sauvignon | Roots | 10 cm high plants grown in Magenta boxes |
|  |  | sT7aVVM017A19080 | 161716251 | Cabernet Sauvignon | Roots | 10 cm high plants grown in Magenta boxes |
|  |  | VVD114A02_371207 | 30131434 | Chardonnay | Berries | Mixed; 8, 9, 11, 13, 15, 16 weeks daf |
|  |  | VVH044B03_746033 | 71863743 | Cabernet Sauvignon | Nectary of flowers | 25 - modified E-L system |
|  |  | S2B21484 | 110701706 | Thompson-seedless | Bud |  |
|  |  | WIN057.C21_E08 | 110374112 | Cabernet Sauvignon | Flower, leaf and root | Flower, pre-anthesis; leaf, fully expanded; root, produced by air-layering |
|  |  | CSECS138A04_CELu0001 | 45770863 | Cabernet Sauvignon | Cell Suspension Culture |  |
|  |  | VVD045G06_352445 | 30135568 | Chardonnay | Berries | Mixed; 8, 9, 11, 13, 15, 16 weeks daf |
|  |  | CAP0005_IIIR_E04 | 34549201 | Cabernet Sauvignon | Petiole | Onset of Veraison (berry softening) |
|  |  | sT7aVVM027C21093 | 161720635 | Cabernet Sauvignon | Roots | 10 cm high plants grown in Magenta boxes |
|  |  | VVH033F05_744161 | 71860113 | Cabernet Sauvignon | Nectary of flowers | 25 - modified E-L system |
|  |  | VVH044D07_746089 | 71863771 | Cabernet Sauvignon | Nectary of flowers | 25 - modified E-L system |
|  |  | VVA024E06_54977 | 18459463 | Chardonnay | Leaf | Juvenile and adult |
|  |  | CSECS143A12_NECu0025 | 45771220 | Cabernet Sauvignon | Nectary of flowers | 25 - modified E-L system |
|  |  | V-B-126F03 | 33962082 | Vitis aestivalis/Norton | Leaf | Young leaf |
|  |  | CSECS158B02_POSu0038 | 45368082 | Cabernet Sauvignon | Fruit with seeds removed | 38 - modified E-L system |
|  |  | S9B04015 | 110722500 | Thompson-seedless | Berries | Ripening Berries |
|  |  | CSECS136A12_CELu0001 | 45770743 | Cabernet Sauvignon | Cell Suspension Culture |  |
|  |  | FAMU_USDA_FP_2795 | 51576936 | Vitis shuttleworthii | Entire tendril, leaves, bud, flowers | At blooming |
|  |  | CSECS140H07_CELu0001 | 45771063 | Cabernet Sauvignon | Cell Suspension Culture |  |
|  |  | VVH027F04_743071 | 71863151 | Cabernet Sauvignon | Nectary of flowers | 25 - modified E-L system |
|  |  | EST 13111 | 32458276 | Chardonnay | Fruit pedicle | Green stage |
|  |  | S8B04635 | 110720671 | Thompson-seedless | Fruit | Veraison |
|  |  | VVH034H07_744379 | 71860222 | Cabernet Sauvignon | Nectary of flowers | 25 - modified E-L system |
|  |  | VVB003D11_403717 | 32246866 | Chardonnay | Leaf | Juvenile and adult |
|  |  | sT7aVVM006O07018 | 161713571 | Cabernet Sauvignon | Roots | 10 cm high plants grown in Magenta boxes |
|  |  | VVL095D11_690894 | 71885579 | Cabernet Sauvignon | Fruit with seeds removed | Mixed 36-38 - modified E-L system (Brix > 15) |
|  |  | VVD129H03_372555 | 30125993 | Chardonnay | Berries | Mixed; 8, 9, 11, 13, 15, 16 weeks daf |
|  |  | GERMO01_000010 | 37189025 | Regent | Shoot tip | Growing shoot tip (ca. 1 cm long) |
|  |  | VVH054E01_747917 | 71861080 | Cabernet Sauvignon | Nectary of flowers | 25 - modified E-L system |
|  |  | VVA024E06_391667 | 30320775 | Chardonnay | Leaf | Juvenile and adult |
|  |  | EST 13192 | 32458357 | Chardonnay | Fruit pedicle | Green stage |
|  |  | sT7aVVM_AER83H08 | 161706248 | Cabernet Sauvignon | Roots | 10 cm high plants grown in Magenta boxes |
|  |  | VVB031C05_134102 | 27580927 | Chardonnay | Leaf | Juvenile and adult |
|  |  | VVB003D11_124224 | 27578686 | Chardonnay | Leaf | Juvenile and adult |
|  |  | VVL111C10_693628 | 71886946 | Cabernet Sauvignon | Fruit with seeds removed | Mixed 36-38 - modified E-L system (Brix > 15) |
|  |  | SCB02262 | 110730672 | Thompson-seedless | Inflorescence | Inflorescence with GA3 |
|  |  | VVL116B08_694502 | 71887383 | Cabernet Sauvignon | Fruit with seeds removed | Mixed 36-38 - modified E-L system (Brix > 15) |
|  |  | SCB02275 | 110732602 | Thompson-seedless | Inflorescence | Inflorescence with GA3 |
|  |  | VVL092A05_690292 | 71885278 | Cabernet Sauvignon | Fruit with seeds removed | Mixed 36-38 - modified E-L system (Brix > 15) |
|  |  | FAMU_USDA_FP_6094 | 51580235 | Vitis shuttleworthii | Entire tendril, leaves, bud, flowers | At blooming |
|  |  | S8B04526 | 110719090 | Thompson-seedless | Fruit | Veraison |
| *VvMPK13* | GSVIVT01026984001 | WIN051.C21_H01 | 110381451 | Cabernet Sauvignon | Flower, leaf and root | Flower, pre-anthesis; leaf, fully expanded; root, produced by air-layering |
|  |  | V-B-126F03 | 33962082 | Vitis aestivalis/Norton | Leaf | Young leaf |
|  |  | sT7aVVM_AER70E01 | 161706043 | Cabernet Sauvignon | Roots | 10 cm high plants grown in Magenta boxes |
|  |  | VV_PEd19f03.b1 | 156739002 | Perlette | Bud | Young |
|  |  | CA48LN07IVF-B4 | 26261816 | Cabernet Sauvignon | Leaf | Late season sample |
|  |  | CSECS063F01_PREu0032 | 34362790 | Cabernet Sauvignon | Fruit with seeds removed | 32 - modified E-L system |
|  |  | VVC064D01_230676 | 27586442 | Chardonnay | Berries | Mixed; 8, 9, 11, 13, 15, 16 weeks daf |
|  |  | CA12LIO2IVR_F08 | 26258173 | Cabernet Sauvignon | Leaf | Late season sample |
|  |  | VV_PEd19f03.g1 | 156739003 | Perlette | Bud | Young |
|  |  | CA48LN07IVR-B3 | 26262854 | Cabernet Sauvignon | Leaf | Late season sample |
|  |  | C1G08048 | 110686022 | Carmenere | Fruit bud cluster |  |
|  |  | CSECS043E03_VERu0035 | 34363711 | Cabernet Sauvignon | Fruit with seeds removed | 35 - modified E-L system |
|  |  | SBB03081 | 110727011 | Thompson-seedless | Inflorescence |  |
|  |  | WIN085.C21_P21 | 110397781 | Cabernet Sauvignon | Seed | Fruit set to maturity |
|  |  | VVD059E04_350867 | 30134779 | Chardonnay | Berries | Mixed; 8, 9, 11, 13, 15, 16 weeks daf |
|  |  | CA12LIO2IVF_F08 | 26257224 | Cabernet Sauvignon | Leaf | Late season sample |
|  |  | VRK77 | 47061151 | Vitis riparia | Bud | Paradormant |
|  |  | SCB03019 | 110731990 | Thompson-seedless | Inflorescence |  |
|  |  | WIN0820.C21_K19 | 110399790 | Cabernet Sauvignon | Seed | Fruit set to maturity |
|  |  | sT7aVVM021N12036 | 161717055 | Cabernet Sauvignon | Roots | 10 cm high plants grown in Magenta boxes |
|  |  | VVC064D01_396059 | 30329963 | Chardonnay | Berries | Mixed; 8, 9, 11, 13, 15, 16 weeks daf |
|  |  | VRK77T7 | 47061152 | Vitis riparia | Bud | Paradormant |
|  |  | VVC063F04_230552 | 27586380 | Chardonnay | Berries | Mixed; 8, 9, 11, 13, 15, 16 weeks daf |
| *VvMPK14* | GSVIVT01038192001 | S2B20192 | 110700707 | Thompson-seedless | Bud |  |
|  |  | CSECS054D07_VERu0035 | 34363900 | Cabernet Sauvignon | Fruit with  seeds removed | 35 - modified E-L system |
|  |  | sT7aVVM017H04010 | 161718819 | Cabernet Sauvignon | Roots | 10 cm high plants grown in Magenta boxes |
|  |  | CAB20006_IIIa_Ra_B12 | 33404655 | Cabernet Sauvignon | Flower - Bloom | Bloom |
|  |  | sT7aVVM010F05027 | 161715173 | Cabernet Sauvignon | Roots | 10 cm high plants grown in Magenta boxes |
|  |  | sT7aVVM007I22087 | 161715065 | Cabernet Sauvignon | Roots | 10 cm high plants grown in Magenta boxes |
|  |  | sT7aVVM_AER4F02 | 161709501 | Cabernet Sauvignon | Roots | 10 cm high plants grown in Magenta boxes |
|  |  | sT7aVVM_AER67F12 | 161707221 | Cabernet Sauvignon | Roots | 10 cm high plants grown in Magenta boxes |
|  |  | sT7aVVM012G08026 | 161717362 | Cabernet Sauvignon | Roots | 10 cm high plants grown in Magenta boxes |
|  |  | sT7aVVM012G09041 | 161717363 | Cabernet Sauvignon | Roots | 10 cm high plants grown in Magenta boxes |
|  |  | sT7aVVM004L06021 | 161714347 | Cabernet Sauvignon | Roots | 10 cm high plants grown in Magenta boxes |
|  |  | sT7aVVM020L04006 | 161716253 | Cabernet Sauvignon | Roots | 10 cm high plants grown in Magenta boxes |
|  |  | WIN102.C21_L03 | 110405255 | Muscat Hamburg | Pericarp | Fruit set to maturity |
|  |  | WIN1018.C21_N08 | 110408740 | Muscat Hamburg | Pericarp | Fruit set to maturity |
|  |  | S7B00921 | 110716506 | Thompson-seedless | Berry | Berries 14mm with GA3 |
|  |  | VVB025A06_133708 | 27580730 | Chardonnay | Leaf | Juvenile and adult |
|  |  | VVD114A02_371207 | 30131434 | Chardonnay | Berries | Mixed; 8, 9, 11, 13, 15, 16 weeks daf |
|  |  | C4B00676 | 110696399 | Carmenere | Clusters | Veraison |
|  |  | CSECS061B12_VERu0035 | 34364337 | Cabernet Sauvignon | Fruit with seeds removed | 35 - modified E-L system |
|  |  | VVI133D09_603902 | 71876332 | Cabernet Sauvignon | Inflorescence including flowers | 12 - modified E-L system |
|  |  | VVB011H09_125480 | 27579314 | Chardonnay | Leaf | Juvenile and adult |
|  |  | S4B04459 | 110705998 | Thompson-seedless | Fruit | Fruits 2-3 mm |
|  |  | RADIC01_001894 | 37186714 | Pinot Noir | Roots | Young roots |
|  |  | CGF1000811_H07 | 33408416 | Cabernet Sauvignon | Stem | Pre-bloom (10-11 days before bloom) |
|  |  | WIN0513.C21_N13 | 110372708 | Cabernet Sauvignon | Flower, leaf and root | Flower, pre-anthesis; leaf, fully expanded; root, produced by air-layering |
|  |  | VVD130B05_372595 | 30126013 | Chardonnay | Berries | Mixed; 8, 9, 11, 13, 15, 16 weeks daf |
|  |  | WIN0417.C21_K16 | 110369082 | Cabernet Sauvignon | Pericarp | Fruit set to maturity |
|  |  | WIN087.C21_D12 | 110403029 | Cabernet Sauvignon | Seed | Fruit set to maturity |
|  |  | VVI123A04_602164 | 71875463 | Cabernet Sauvignon | Inflorescence including flowers | 12 - modified E-L system |
|  |  | WIN0412.C21_F07 | 110367149 | Cabernet Sauvignon | Pericarp | Fruit set to maturity |
|  |  | FAMU_USDA_FP_3661 | 51577802 | Vitis shuttleworthii | Entire tendril, leaves, bud, flowers | At blooming |
|  |  | WIN107.C21_I08 | 110412598 | Muscat Hamburg | Pericarp | Fruit set to maturity |
|  |  | S6B06338 | 110713988 | Thompson-seedless | Fruit | Fruits 7-9 mm |
|  |  | WIN0524.C21_I09 | 110376972 | Cabernet Sauvignon | Flower, leaf and root | Flower, pre-anthesis; leaf, fully expanded; root, produced by air-layering |
|  |  | WIN1034.C21_L02 | 110411727 | Muscat Hamburg | Pericarp | Fruit set to maturity |
|  |  | VV_PEb08g04.b1 | 156729427 | Perlette | Bud | Mature |
|  |  | VV_PEb04f03.b1 | 156732346 | Perlette | Bud | Mature |
|  |  | VV_PEb04f03.g1 | 156728757 | Perlette | Bud | Mature |
|  |  | S7B05109 | 110715356 | Thompson-seedless | Berries | Berries 14mm with GA3 |
|  |  | CGF1000814_H07 | 33408675 | Cabernet Sauvignon | Stem | Pre-bloom (10-11 days before bloom) |
