# Supplementary material for: Mitogen-activated protein kinase cascades in Vitis vinifera
Source: Front Plant Sci. 2015 Jul 22;6:556. doi: 10.3389/fpls.2015.00556 (PMC4511077; doi:10.3389/fpls.2015.00556)
Supplement: Supplementary file 2 [file Table2.DOC]

**Supplementary Table 2.** **Expressed sequence tags (ESTs) identified for the MAPKK subfamily in *Vitis vinifera*.** The protein name, *Vitis* proteome 12× ID, GenBank ID, EST name, cultivar/tissue type, and development stage are given for each gene.

| **Name** | ***Vitis* 12X ID** | **EST Name** | **GenBank ID** | **Species/Cultivar** | **Tissue Type** | **Development Stage** |
| --- | --- | --- | --- | --- | --- | --- |
| *VvMKK1* | GSVIVT01008476001 | WIN1128.C21_K18 | 110420492 | Muscat Hamburg | Berries | Anthesis flower to prior to veraison |
|  |  | sT7aVVM020B17079 | 161717511 | Cabernet Sauvignon | Roots | 10 cm high plants grown in Magenta boxes |
|  |  | sT7aVVM026H07026 | 161721061 | Cabernet Sauvignon | Roots | 10 cm high plants grown in Magenta boxes |
|  |  | sT7aVVM028L23086 | 161721431 | Cabernet Sauvignon | Roots | 10 cm high plants grown in Magenta boxes |
|  |  | sT7aVVM_AER40C03 | 161711412 | Cabernet Sauvignon | Roots | 10 cm high plants grown in Magenta boxes |
|  |  | WIN0524.C21_N13 | 110377078 | Cabernet Sauvignon | Flower, leaf and root | Flower, pre-anthesis; leaf, fully expanded;  root, produced by air-layering |
|  |  | VVD033C08_347515 | 30133102 | Chardonnay | Berries | Mixed; 8, 9, 11, 13, 15, 16 weeks daf |
|  |  | S1G08474 | 110699867 | Thompson-seedless | Fruit and flower | Fruits and flowers treated with GA3 |
|  |  | L11_67_Sh_CT_P1_F08.ab1 1 295 | 134030291 | Vitis arizonica x Vitis rupestris | Shoot | Vegetative stage-control |
|  |  | V-B-129D08 | 33962537 | Vitis aestivalis/Norton | Leaf | Young leaf |
|  |  | V-B-19E02 | 28602563 | Vitis aestivalis/Norton | Leaf | Young leaf |
|  |  | CSECS147A06_NECu0025 | 45771495 | Cabernet Sauvignon | Nectary of flowers | 25 - modified E-L system |
|  |  | VVI019D08_585908 | 71868979 | Cabernet Sauvignon | Inflorescence  including flowers | 12 - modified E-L system |
|  |  | S5B02791 | 110707863 | Thompson-seedless | Fruit | Fruits 7-9 mm |
|  |  | VVI058B08_591504 | 71871777 | Cabernet Sauvignon | Inflorescence including flowers | 12 - modified E-L system |
|  |  | WIN0563.C21_D03 | 110390038 | Cabernet Sauvignon | Flower, leaf and root | Flower, pre-anthesis; leaf, fully expanded; root, produced by air-layering |
|  |  | VVI032C03_587944 | 71869997 | Cabernet Sauvignon | Inflorescence including flowers | 12 - modified E-L system |
|  |  | VVI122F02_602098 | 71875430 | Cabernet Sauvignon | Inflorescence including flowers | 12 - modified E-L system |
|  |  | VVI151E01_606988 | 77577818 | Cabernet Sauvignon | Inflorescence including flowers | 12 - modified E-L system |
|  |  | VVI105A09_599122 | 71866772 | Cabernet Sauvignon | Inflorescence including flowers | 12 - modified E-L system |
|  |  | C3B00752 | 110692785 | Carmenere | Clusters | Clusters 4 cm |
|  |  | VRK325T7 | 48941396 | Vitis riparia | Bud | Paradormant |
|  |  | VVI155C04_607628 | 77578388 | Cabernet Sauvignon | Inflorescence including flowers | 12 - modified E-L system |
|  |  | V-B-128G12 | 33962438 | Vitis aestivalis/Norton | Leaf | Young leaf |
|  |  | VVI086B08_595990 | 71874020 | Cabernet Sauvignon | Inflorescence including flowers | 12 - modified E-L system |
|  |  | VRK325 | 48941395 | Vitis riparia | Bud | Paradormant |
|  |  | VVH040A01_745273 | 71860669 | Cabernet Sauvignon | Nectary of flowers | 25 - modified E-L system |
|  |  | SCB03573 | 110730573 | Thompson-seedless | Inflorescence | Inflorescence with GA3 |
|  |  | WIN0535.C21_G13 | 110380388 | Cabernet Sauvignon | Flower, leaf and root | Flower, pre-anthesis; leaf, fully expanded; root, produced by air-layering |
|  |  | RADIC01_000588 | 37185671 | Pinot Noir | Roots | Young roots |
|  |  | VVI212H08_617316 | 77587367 | Cabernet Sauvignon | Inflorescence including flowers | 12 - modified E-L system |
|  |  | WIN0540.C21_J03 | 110382745 | Cabernet Sauvignon | Flower, leaf and root | Flower, pre-anthesis; leaf, fully expanded; root, produced by air-layering |
|  |  | S2B24680 | 110703526 | Thompson-seedless | Bud |  |
|  |  | VVI088F04_596380 | 71865401 | Cabernet Sauvignon | Inflorescence including flowers | 12 - modified E-L system |
|  |  | VVI079E04_594926 | 71873488 | Cabernet Sauvignon | Inflorescence including flowers | 12 - modified E-L system |
|  |  | WIN059.C21_K14 | 110374314 | Cabernet Sauvignon | Flower, leaf and root | Flower, pre-anthesis; leaf, fully expanded; root, produced by air-layering |
|  |  | S2B21063 | 110700537 | Thompson-seedless | Bud |  |
|  |  | SBB01503 | 110725672 | Thompson-seedless | Inflorescence |  |
|  |  | WIN0544.C21_M06 | 110384056 | Cabernet Sauvignon | Flower, leaf and root | Flower, pre-anthesis; leaf, fully expanded; root, produced by air-layering |
| *VvMKK2* | GSVIVT01015155001 | sT7aVVM024E01011 | 161717958 | Cabernet Sauvignon | Roots | 10 cm high plants grown in Magenta boxes |
|  |  | sT7aVVM027M07020 | 161721441 | Cabernet Sauvignon | Roots | 10 cm high plants grown in Magenta boxes |
|  |  | sT7aVVM013J13055 | 161718247 | Cabernet Sauvignon | Roots | 10 cm high plants grown in Magenta boxes |
|  |  | CAB20001_IVa_Fa_G12 | 33401804 | Cabernet Sauvignon | Flower - Bloom | Bloom |
|  |  | WIN0542.C21_E22 | 110383291 | Cabernet Sauvignon | Flower, leaf and root | Flower, pre-anthesis; leaf, fully expanded; root, produced by air-layering |
|  |  | CSECS124E04_PREn0028 | 51051532 | Cabernet Sauvignon | Fruit | 28 - modified E-L system |
|  |  | CAB10003_Ia_Fa_G06 | 30252938 | Cabernet Sauvignon | Flower - Pre-bloom | Pre-bloom |
|  |  | S1G06205 | 110699026 | Thompson-seedless | Fruit and flower |  |
|  |  | WIN1139.C21_M02 | 110424147 | Muscat Hamburg | Berries | Anthesis flower to prior to veraison |
|  |  | WIN0210.TB24.1_H20 | 110362388 | Cabernet Sauvignon | Flower, leaf and root | Flower, pre-anthesis; leaf, fully expanded; root, produced by air-layering |
|  |  | S6B04474 | 110712132 | Thompson-seedless | Fruit | Fruits 7-9 mm |
|  |  | S6B05469 | 110712219 | Thompson-seedless | Fruit | Fruits 7-9 mm |
|  |  | SBB05627 | 110728640 | Thompson-seedless | Inflorescence |  |
|  |  | S1G07114 | 110699759 | Thompson-seedless | Fruit and flower | Fruits and flowers treated with GA3 |
|  |  | WIN101.C21_D24 | 110404805 | Muscat Hamburg | Pericarp | Fruit set to maturity |
|  |  | INFIO01_000734 | 37190557 | Regent | Inflorescence | Young inflorescence before flowering |
|  |  | WIN1116.C21_E20 | 110416568 | Muscat Hamburg | Berries | Anthesis flower to prior to veraison |
|  |  | VVH017D12_741273 | 71862252 | Cabernet Sauvignon | Nectary of flowers | 25 - modified E-L system |
|  |  | S8B04980 | 110719234 | Thompson-seedless | Fruit | Veraison |
|  |  | VVH013C12_740519 | 71861875 | Cabernet Sauvignon | Nectary of flowers | 25 - modified E-L system |
|  |  | CAB2SG0001_IIaR_B09 | 28967191 | Cabernet Sauvignon | Berries | Veraison |
|  |  | S6B00854 | 110711245 | Thompson-seedless | Fruit | Fruits 7-9 mm |
|  |  | VVH021A09_741921 | 71862576 | Cabernet Sauvignon | Nectary of flowers | 25 - modified E-L system |
|  |  | CAB20001_IVa_Ra_G12 | 33401876 | Cabernet Sauvignon | Flower - Bloom | Bloom |
|  |  | CAB2SG0001IIF_B09 | 28966889 | Cabernet Sauvignon | Berries | Veraison |
|  |  | VVC037F06_395191 | 30329529 | Chardonnay | Berries | Mixed; 8, 9, 11, 13, 15, 16 weeks daf |
|  |  | WIN087.C21_B11 | 110403013 | Cabernet Sauvignon | Seed | Fruit set to maturity |
|  |  | CAB20005_Ia_Ra_A04 | 33404470 | Cabernet Sauvignon | Flower - Bloom | Bloom |
|  |  | FAMU_USDA_FP_3534 | 51577675 | Vitis shuttleworthii | Entire tendril, leaves, bud, flowers | At blooming |
|  |  | VVB135E01_406077 | 32267926 | Chardonnay | Leaf | Juvenile and adult |
|  |  | V-B-13H04 | 28602085 | Vitis aestivalis/Norton | Leaf | Young leaf |
|  |  | VVC037F06_396587 | 30330227 | Chardonnay | Berries | Mixed; 8, 9, 11, 13, 15, 16 weeks daf |
|  |  | WIN012.BR_A24 | 110359352 | Cabernet Sauvignon | Pericarp | Fruit set to maturity |
|  |  | S1G06924 | 110699634 | Thompson-seedless | Fruit and flower | Fruits and flowers treated with GA3 |
|  |  | CAB20005_Ia_Ra_H10 | 33404549 | Cabernet Sauvignon | Flower - Bloom | Bloom |
|  |  | CAB20005_Ia_Fa_A04 | 33404398 | Cabernet Sauvignon | Flower - Bloom | Bloom |
|  |  | EST 18599 | 46911080 | Cabernet Sauvignon | Fruit without seeds | Ripe Stage |
|  |  | S1G06912 | 110699630 | Thompson-seedless | Fruit and flower | Fruits and flowers treated with GA3 |
|  |  | VVC037F06_139702 | 27584071 | Chardonnay | Berries | Mixed; 8, 9, 11, 13, 15, 16 weeks daf |
|  |  | CAB20005_Ia_Fa_H10 | 33404467 | Cabernet Sauvignon | Flower - Bloom | Bloom |
| *VvMKK3* | GSVIVT01015283001 | WIN1147.C21_G01 | 110426678 | Muscat Hamburg | Berries | Anthesis flower to prior to veraison |
|  |  | WIN1141.C21_L12 | 110424787 | Muscat Hamburg | Berries | Anthesis flower to prior to veraison |
|  |  | FAMU_USDA_FP_3534 | 51577675 | Vitis shuttleworthii | Entire tendril, leaves, bud, flowers | At blooming |
| *VvMKK4* | GSVIVT01016115001 | S9B01944 | 110721322 | Thompson-seedless | Berries | Ripening Berries |
|  |  | CAbud0007_IVR_D10 | 34545856 | Cabernet Sauvignon | Bud | Pre-bloom (10-11 days before bloom) |
|  |  | WIN023.C21_H24 | 110360688 | Cabernet Sauvignon | Flower, leaf and root | Flower, pre-anthesis; leaf, fully expanded; root, produced by air-layering |
|  |  | WIN106.C21_B17 | 110412164 | Muscat Hamburg | Pericarp | Fruit set to maturity |
|  |  | CAP0007_IIIR_G09 | 34550512 | Cabernet Sauvignon | Petiole | Onset of Veraison (berry softening) |
|  |  | sT7aVVM001L18069 | 161712340 | Cabernet Sauvignon | Roots | 10 cm high plants grown in Magenta boxes |
|  |  | sT7aVVM011I17071 | 161716950 | Cabernet Sauvignon | Roots | 10 cm high plants grown in Magenta boxes |
|  |  | WIN0550.C21_K07 | 110386192 | Cabernet Sauvignon | Flower, leaf and root | Flower, pre-anthesis; leaf, fully expanded; root, produced by air-layering |
|  |  | GEMMA01_001392 | 37188514 | Pinot Noir | Bud | Bud swelling |
|  |  | C4B05333 | 110697461 | Carmenere | Clusters | Veraison |
|  |  | SBB03525 | 110727997 | Thompson-seedless | Inflorescence |  |
|  |  | CAbud0007_IVF_D10 | 34545778 | Cabernet Sauvignon | Bud | Pre-bloom (10-11 days before bloom) |
|  |  | S5B05611 | 110709958 | Thompson-seedless | Fruit | Fruits 7-9 mm |
|  |  | RADIC01_000010 | 37185188 | Pinot Noir | Roots | Young roots |
|  |  | CAP0007_IIIF_G09 | 34550431 | Cabernet Sauvignon | Petiole | Onset of Veraison (berry softening) |
| *VvMKK5* | GSVIVT01032414001 | WIN1119.C21_J08 | 110417598 | Muscat Hamburg | Berries | Anthesis flower to prior to veraison |
