# Supplementary material for: Mitogen-activated protein kinase cascades in Vitis vinifera
Source: Front Plant Sci. 2015 Jul 22;6:556. doi: 10.3389/fpls.2015.00556 (PMC4511077; doi:10.3389/fpls.2015.00556)
Supplement: Supplementary file 3 [file Table3.DOC]

**Supplementary Table 3.** **Expressed sequence taqs (ESTs) identified for MAPKKK subfamily in *Vitis vinifera*.**The protein name, *Vitis* proteome 12x ID, GenBank ID, EST name, cultivar/tissue type, and development stage are given for each gene.

| **Name** | ***Vitis* 12X ID** | **EST Name** | **GenBank ID** | **Species/Cultivar** | **Tissue Type** | **Development Stage** |
| --- | --- | --- | --- | --- | --- | --- |
| *VviMAPKKK1* | GSVIVT01000047001 | VVI173E06_610770 | 77581253 | Cabernet Sauvignon | Inflorescence including flowers | 12 - modified E-L system |
| *VviMAPKKK2* | GSVIVT01000256001 | - | - | - | - | - |
| *VviMAPKKK3* | GSVIVT01001193001 | WIN011.BR_B21 | 110358885 | Cabernet Sauvignon | Pericarp | Fruit set to maturity |
|  |  | VVB116C04_382547 | 30327575 | Chardonnay | Leaf | Juvenile and adult |
|  |  | CSECS001F08_PREu0032 | 34361579 | Cabernet Sauvignon | Fruit with seeds removed | 32 - modified E-L system |
|  |  | VV_PEb04h10.g1 | 156732355 | Perlette | Bud | Mature |
|  |  | VVB140C08_406975 | 32268375 | Chardonnay | Leaf | Juvenile and adult |
|  |  | WIN1149.C21_K14 | 110427431 | Muscat Hamburg | Berries | Anthesis flower to prior to veraison |
|  |  | VVL139B10_698506 | 71889385 | Cabernet Sauvignon | Fruit with seeds removed | Mixed 36-38 - modified E-L system (Brix > 15) |
|  |  | VVB077A06_333910 | 30324129 | Chardonnay | Leaf | Juvenile and adult |
|  |  | WIN1018.C21_L03 | 110408701 | Muscat Hamburg | Pericarp | Fruit set to maturity |
|  |  | WIN1128.C21_E17 | 110420368 | Muscat Hamburg | Berries | Anthesis flower to prior to veraison |
|  |  | S6B03153 | 110713197 | Thompson-seedless | Fruit | Fruits 7-9 mm |
|  |  | VVH032A09_743867 | 71859966 | Cabernet Sauvignon | Nectary of flowers | 25 - modified E-L system |
|  |  | CAB20002_IIIa_Ra_G08 | 33402132 | Cabernet Sauvignon | Flower - Bloom | Bloom |
|  |  | VVB200A01_431961 | 32248528 | Chardonnay | Leaf | Juvenile and adult |
|  |  | WIN048.C21_B14 | 110370551 | Cabernet Sauvignon | Pericarp | Fruit set to maturity |
|  |  | VVD006C01_344023 | 30129045 | Chardonnay | Berries | Mixed; 8, 9, 11, 13, 15, 16 weeks daf |
|  |  | GEMMA01_001161 | 37188291 | Pinot Noir | Bud | Bud swelling |
|  |  | C4B06976 | 110697646 | Carmenere | Clusters | Veraison |
|  |  | CAB20002_IIIa_Fa_G08 | 33402047 | Cabernet Sauvignon | Flower - Bloom | Bloom |
|  |  | WIN0213.TB24_M06 | 110363284 | Cabernet Sauvignon | Flower, leaf and root | Flower, pre-anthesis; leaf, fully expanded; root, produced by air-layering |
|  |  | WIN017.C21_I14 | 110360075 | Cabernet Sauvignon | Pericarp | Fruit set to maturity |
|  |  | CSECS145H07_NECu0025 | 45771417 | Cabernet Sauvignon | Nectary of flowers | 25 - modified E-L system |
|  |  | WIN0546.C21_B07 | 110384789 | Cabernet Sauvignon | Flower, leaf and root | Flower, pre-anthesis; leaf, fully expanded; root, produced by air-layering |
|  |  | VV_PEd0012h11.g1 | 156736380 | Perlette | Bud | Young |
|  |  | VV_PEd0012a10.b1 | 156736209 | Perlette | Bud | Young |
|  |  | VVB168F01_412121 | 32270948 | Chardonnay | Leaf | Juvenile and adult |
|  |  | VVB102F06_341509 | 30326733 | Chardonnay | Leaf | Juvenile and adult |
|  |  | VVB111H11_342441 | 30327199 | Chardonnay | Leaf | Juvenile and adult |
|  |  | VVB190C01_430185 | 32247640 | Chardonnay | Leaf | Juvenile and adult |
|  |  | VVB082B03_335186 | 30324767 | Chardonnay | Leaf | Juvenile and adult |
|  |  | VVB080C04_334852 | 30324600 | Chardonnay | Leaf | Juvenile and adult |
|  |  | VVB104F10_341873 | 30326915 | Chardonnay | Leaf | Juvenile and adult |
| *VviMAPKKK4* | GSVIVT01001690001 | CGF1000661_H09 | 33406408 | Cabernet Sauvignon | Stem | Pre-bloom (10-11 days before bloom) |
|  |  | VVA003H11_52437 | 18458149 | Chardonnay | Leaf | Juvenile and adult |
|  |  | VVI205D08_616074 | 77586220 | Cabernet Sauvignon | Inflorescence including flowers | 12 - modified E-L system |
| *VviMAPKKK5* | GSVIVT01002332001 | VVL092E05_690380 | 71885322 | Cabernet Sauvignon | Fruit with seeds removed | Mixed 36-38 - modified E-L system (Brix > 15) |
|  |  | CSECS215A09_5_PREn0028 | 87586507 | Cabernet Sauvignon | Fruit | 28 - modified E-L system |
|  |  | VVL148E10_700130 | 71890197 | Cabernet Sauvignon | Fruit with seeds removed | Mixed 36-38 - modified E-L system (Brix > 15) |
|  |  | CGF1000661_H09 | 33406408 | Cabernet Sauvignon | Stem | Pre-bloom (10-11 days before bloom) |
|  |  | CGF1000662_H09 | 33406309 | Cabernet Sauvignon | Stem | Pre-bloom (10-11 days before bloom) |
| *VviMAPKKK6* | GSVIVT01004254001 | C2C05708 | 110689010 | Carmenere | Bud - cluster |  |
|  |  | WIN0211.TB24_G21 | 110362641 | Cabernet Sauvignon | Flower, leaf and root | Flower, pre-anthesis; leaf, fully expanded; root, produced by air-layering |
|  |  | WIN0553.C21_F01 | 110387023 | Cabernet Sauvignon | Flower, leaf and root | Flower, pre-anthesis; leaf, fully expanded; root, produced by air-layering |
|  |  | S1G05421 | 110698751 | Thompson-seedless | Fruit and flower | Fruits and flowers treated with GA3 |
|  |  | WIN0420.C21_M16 | 110370097 | Cabernet Sauvignon | Pericarp | Fruit set to maturity |
|  |  | CAP0003_IR_A10 | 34548201 | Cabernet Sauvignon | Petiole | Onset of Veraison (berry softening) |
|  |  | sT7aVVM001C02013 | 161712085 | Cabernet Sauvignon | Roots | 10 cm high plants grown in Magenta boxes |
|  |  | VV_PEa017c08.b1 | 156724070 | Perlette | Bud | Mature |
|  |  | CAP0002_IIF_H07 | 34547554 | Cabernet Sauvignon | Petiole | Onset of Veraison (berry softening) |
|  |  | VVG055G11_761805 | 71859390 | Cabernet Sauvignon | Cell Suspension Culture |  |
|  |  | WIN0518.C21_C05 | 110374984 | Cabernet Sauvignon | Flower, leaf and root | Flower, pre-anthesis; leaf, fully expanded; root, produced by air-layering |
|  |  | CSECS004E06_PREU0032 | 34361725 | Cabernet Sauvignon | Fruit with seeds removed | 32 - modified E-L system |
|  |  | WIN1116.C21_D18 | 110416543 | Muscat Hamburg | Berries | Anthesis flower to prior to veraison |
|  |  | CAP0002_IIR_H07 | 34547637 | Cabernet Sauvignon | Petiole | Onset of Veraison (berry softening) |
|  |  | WIN0417.C21_D20 | 110368943 | Cabernet Sauvignon | Pericarp | Fruit set to maturity |
| *VviMAPKKK7* | GSVIVT01007446001 | C2C05708 | 110689010 | Carmenere | Bud - cluster |  |
|  |  | WIN0211.TB24_G21 | 110362641 | Cabernet Sauvignon | Flower, leaf and root | Flower, pre-anthesis; leaf, fully expanded; root, produced by air-layering |
|  |  | WIN0553.C21_F01 | 110387023 | Cabernet Sauvignon | Flower, leaf and root | Flower, pre-anthesis; leaf, fully expanded; root, produced by air-layering |
|  |  | VVG055G11_761805 | 71859390 | Cabernet Sauvignon | Cell Suspension Culture |  |
|  |  | CAP0003_IR_A10 | 34548201 | Cabernet Sauvignon | Petiole | Onset of Veraison (berry softening) |
|  |  | sT7aVVM001C02013 | 161712085 | Cabernet Sauvignon | Roots | 10 cm high plants grown in Magenta boxes |
|  |  | WIN0420.C21_M16 | 110370097 | Cabernet Sauvignon | Pericarp | Fruit set to maturity |
|  |  | CAP0002_IIF_H07 | 34547554 | Cabernet Sauvignon | Petiole | Onset of Veraison (berry softening) |
|  |  | VV_PEa017c08.b1 | 156724070 | Perlette | Bud | Mature |
|  |  | WIN0518.C21_C05 | 110374984 | Cabernet Sauvignon | Flower, leaf and root | Flower, pre-anthesis; leaf, fully expanded; root, produced by air-layering |
|  |  | CSECS004E06_PREU0032 | 34361725 | Cabernet Sauvignon | Fruit with seeds removed | 32 - modified E-L system |
|  |  | WIN1116.C21_D18 | 110416543 | Muscat Hamburg | Berries | Anthesis flower to prior to veraison |
|  |  | CAP0002_IIR_H07 | 34547637 | Cabernet Sauvignon | Petiole | Onset of Veraison (berry softening) |
|  |  | WIN0417.C21_D20 | 110368943 | Cabernet Sauvignon | Pericarp | Fruit set to maturity |
| *VviMAPKKK8* | GSVIVT01007637001 | VVA009H06_53145 | 18458503 | Chardonnay | Leaf | Juvenile and adult |
|  |  | VV_PEb09b04.b1 | 156729489 | Perlette | Bud | Mature |
|  |  | VVC038B04_140666 | 27584109 | Chardonnay | Berries | Mixed; 8, 9, 11, 13, 15, 16 weeks daf |
|  |  | VVC058E01_416153 | 32245724 | Chardonnay | Berries | Mixed; 8, 9, 11, 13, 15, 16 weeks daf |
|  |  | CAbud0003_IIF_D12 | 34544125 | Cabernet Sauvignon | Bud | Pre-bloom (10-11 days before bloom) |
|  |  | CAB40005_IVa_Fa_A11 | 30301432 | Cabernet Sauvignon | Berries | Berry on stage II, 9 mm |
|  |  | S6B01936 | 110711559 | Thompson-seedless | Fruit | Fruits 7-9 mm |
|  |  | S4B05240 | 110706755 | Thompson-seedless | Fruit | Fruits 2-3 mm |
|  |  | 3-1P1D7 | 111125144 | Cabernet Sauvignon | Leaf blade | Young leaf |
|  |  | VVC058E01_417381 | 32246338 | Chardonnay | Berries | Mixed; 8, 9, 11, 13, 15, 16 weeks daf |
|  |  | VVD039A04_348435 | 30133562 | Chardonnay | Berries | Mixed; 8, 9, 11, 13, 15, 16 weeks daf |
|  |  | VVB079B01_334656 | 30324502 | Chardonnay | Leaf | Juvenile and adult |
|  |  | VVC058E01_229630 | 27585919 | Chardonnay | Berries | Mixed; 8, 9, 11, 13, 15, 16 weeks daf |
|  |  | CA32EN0004_IIIbR_H04 | 28963932 | Cabernet Sauvignon | Leaf | Mid-season leaf material |
|  |  | VVB043G03_324358 | 30321640 | Chardonnay | Leaf | Juvenile and adult |
|  |  | VVB012H05_125652 | 27579400 | Chardonnay | Leaf | Juvenile and adult |
|  |  | VVB027B04_133184 | 27580468 | Chardonnay | Leaf | Juvenile and adult |
| *VviMAPKKK9* | GSVIVT01007646001 | sT7aVVM021D12046 | 161719246 | Cabernet Sauvignon | Roots | 10 cm high plants grown in Magenta boxes |
|  |  | WIN117.C21_D22 | 110429607 | Muscat Hamburg | Berries | Anthesis flower to prior to veraison |
|  |  | CA12LIO2IIFb_H06 | 26257121 | Cabernet Sauvignon | Leaf | Late season sample |
|  |  | BACCA01_000826 | 37184178 | Pinot Noir | Berries | Veraison |
|  |  | WIN056.C21_D16 | 110382036 | Cabernet Sauvignon | Flower, leaf and root | Flower, pre-anthesis; leaf, fully expanded; root, produced by air-layering |
| *VviMAPKKK10* | GSVIVT01007762001 | WIN014.BR_M10 | 122688557 | Cabernet Sauvignon | Pericarp | Fruit set to maturity |
| *VviMAPKKK11* | GSVIVT01007775001 | WIN058.C21_K19 | 110382308 | Cabernet Sauvignon | Flower, leaf and root | Flower, pre-anthesis; leaf, fully expanded; root, produced by air-layering |
|  |  | S6B03153 | 110713197 | Thompson-seedless | Fruit | Fruits 7-9 mm |
|  |  | VvNCCR1dp87 | 33111250 | Chasselas | Leaf |  |
|  |  | VVB077A06_333910 | 30324129 | Chardonnay | Leaf | Juvenile and adult |
|  |  | VVD132D01_372925 | 30126178 | Chardonnay | Berries | Mixed; 8, 9, 11, 13, 15, 16 weeks daf |
|  |  | VV_PEa03c04.b2 | 156724407 | Perlette | Bud | Mature |
|  |  | VV_PEa03c04.g2 | 156724408 | Perlette | Bud | Mature |
|  |  | CAB20002_IIIa_Ra_G08 | 33402132 | Cabernet Sauvignon | Flower - Bloom | Bloom |
|  |  | C3B08504 | 110694673 | Carmenere | Clusters | Clusters 4 cm |
|  |  | VVC025D06_137654 | 27583047 | Chardonnay | Berries | Mixed; 8, 9, 11, 13, 15, 16 weeks daf |
|  |  | VVC025D06_394817 | 30329342 | Chardonnay | Berries | Mixed; 8, 9, 11, 13, 15, 16 weeks daf |
|  |  | EST 3220 | 22009248 | Shiraz | Fruit | Veraison |
|  |  | WIN0418.C21_H14 | 110369346 | Cabernet Sauvignon | Pericarp | Fruit set to maturity |
| *VviMAPKKK12* | GSVIVT01008413001 | CAB70001_IaF_D11 | 30303225 | Cabernet Sauvignon | Berries | Post-Veraison, 18-19 brix |
|  |  | FAMU_USDA_FP_2392 | 51576533 | Vitis shuttleworthii | Entire tendril, leaves, bud, flowers | At blooming |
|  |  | WIN085.C21_A12 | 110397588 | Cabernet Sauvignon | Seed | Fruit set to maturity |
|  |  | C1G06170 | 110685448 | Carmenere | Fruit - bud - clusters |  |
|  |  | VVB151C03_408987 | 32269381 | Chardonnay | Leaf | Juvenile and adult |
|  |  | VVB016A10_131604 | 27579678 | Chardonnay | Leaf | Juvenile and adult |
|  |  | VVB112G01_342565 | 30327261 | Chardonnay | Leaf | Juvenile and adult |
| *VviMAPKKK13* | GSVIVT01008728001 | WIN1132.C21_L02 | 110421786 | Muscat Hamburg | Berries | Anthesis flower to prior to veraison |
|  |  | VVA003H11_52437 | 18458149 | Chardonnay | Leaf | Juvenile and adult |
|  |  | WIN0558.C21_G03 | 110388567 | Cabernet Sauvignon | Flower, leaf and root | Flower, pre-anthesis; leaf, fully expanded; root, produced by air-layering |
| *VviMAPKKK14* | GSVIVT01008938001 | sT7aVVM010D03014 | 161714901 | Cabernet Sauvignon | Roots | 10 cm high plants grown in Magenta boxes |
|  |  | VVA026B12_402785 | 32245309 | Chardonnay | Leaf | Juvenile and adult |
|  |  | WIN051.C21_J06 | 110370919 | Cabernet Sauvignon | Flower, leaf and root | Flower, pre-anthesis; leaf, fully expanded; root, produced by air-layering |
|  |  | VVB006E08_124596 | 27578872 | Chardonnay | Leaf | Juvenile and adult |
|  |  | EST 2358 | 22008386 | Shiraz | Fruit | Veraison |
|  |  | EST 1688 | 22014485 | Shiraz | Fruit | Green stage |
|  |  | VVI211C07_617042 | 77587119 | Cabernet Sauvignon | Inflorescence including flowers | 12 - modified E-L system |
|  |  | EST 2573 | 22008601 | Shiraz | Fruit | Veraison |
|  |  | VVI033F12_588194 | 71870122 | Cabernet Sauvignon | Inflorescence including flowers | 12 - modified E-L system |
|  |  | EST 2883 | 22008911 | Shiraz | Fruit | Veraison |
|  |  | VVH051H10_747453 | 71860848 | Cabernet Sauvignon | Nectary of flowers | 25 - modified E-L system |
|  |  | EST 2282 | 22008310 | Shiraz | Fruit | Veraison |
|  |  | EST 2889 | 22008917 | Shiraz | Fruit | Veraison |
|  |  | WIN1030.C21_G24 | 110410457 | Muscat Hamburg | Pericarp | Fruit set to maturity |
|  |  | EST 18655 | 46911136 | Cabernet Sauvignon | Fruit without seeds | Ripe Stage |
|  |  | VVA026B12_55155 | 18459552 | Chardonnay | Leaf | Juvenile and adult |
|  |  | WIN029.TB24_H07 | 110362100 | Cabernet Sauvignon | Flower, leaf and root | Flower, pre-anthesis; leaf, fully expanded; root, produced by air-layering |
|  |  | CA12EI202IIbF_A03 | 26263982 | Cabernet Sauvignon | Leaf | Mid-season leaf material |
|  |  | VVC052E04_147420 | 27585285 | Chardonnay | Berries | Mixed; 8, 9, 11, 13, 15, 16 weeks daf |
|  |  | CA12EI202IIbR_A03 | 26265965 | Cabernet Sauvignon | Leaf | Mid-season leaf material |
|  |  | VVC023G11_137388 | 27582914 | Chardonnay | Berries | Mixed; 8, 9, 11, 13, 15, 16 weeks daf |
|  |  | WIN059.C21_F11 | 110374528 | Cabernet Sauvignon | Flower, leaf and root | Flower, pre-anthesis; leaf, fully expanded; root, produced by air-layering |
|  |  | WIN025.TB24.1_L16 | 110361077 | Cabernet Sauvignon | Flower, leaf and root | Flower, pre-anthesis; leaf, fully expanded; root, produced by air-layering |
|  |  | VVB006E08_393565 | 30328717 | Chardonnay | Leaf | Juvenile and adult |
|  |  | VVA019A12_120014 | 27755241 | Chardonnay | Leaf | Juvenile and adult |
|  |  | VVA026B12_402283 | 32245058 | Chardonnay | Leaf | Juvenile and adult |
|  |  | CAP0003_IVF_D08 | 34548151 | Cabernet Sauvignon | Pericarp | Onset of Veraison (berry softening) |
| *VviMAPKKK15* | GSVIVT01009192001 | CAB20002_IVa_Fa_C01 | 33402249 | Cabernet Sauvignon | Flower - Bloom | Bloom |
|  |  | FAMU_USDA_FP_1878 | 51576019 | Vitis shuttleworthii | Entire tendril, leaves, bud, flowers | At blooming |
|  |  | VVB119E12_383151 | 30327877 | Chardonnay | Leaf | Juvenile and adult |
|  |  | USDA_FP_131452 | 47090096 | Vitis shuttleworthii | Entire tendril, leaves, bud, flowers | At blooming |
|  |  | VVB063G09_326884 | 30322903 | Chardonnay | Leaf | Juvenile and adult |
|  |  | VVB112D02_342501 | 30327229 | Chardonnay | Leaf | Juvenile and adult |
|  |  | sT7aVVM_AER12C01 | 161708004 | Cabernet Sauvignon | Roots | 10 cm high plants grown in Magenta boxes |
|  |  | sT7aVVM_AE10F08 | 161705877 | Cabernet Sauvignon | Roots | 10 cm high plants grown in Magenta boxes |
|  |  | VVB105C05_339871 | 30325914 | Chardonnay | Leaf | Juvenile and adult |
|  |  | VVB079H05_334796 | 30324572 | Chardonnay | Leaf | Juvenile and adult |
| *VviMAPKKK16* | GSVIVT01009575001 | C2C05708 | 110689010 | Carmenere | Bud - cluster |  |
|  |  | sT7aVVM001C02013 | 161712085 | Cabernet Sauvignon | Roots | 10 cm high plants grown in Magenta boxes |
|  |  | CAP0002_IIF_H07 | 34547554 | Cabernet Sauvignon | Petiole | Onset of Veraison (berry softening) |
| *VviMAPKKK17* | GSVIVT01012031001 | VVA030C12_391887 | 30320885 | Chardonnay | Leaf | Juvenile and adult |
|  |  | VVA030C12_55675 | 18459812 | Chardonnay | Leaf | Juvenile and adult |
|  |  | WIN0515.C21_M20 | 110373313 | Cabernet Sauvignon | Flower, leaf and root | Flower, pre-anthesis; leaf, fully expanded; root, produced by air-layering |
|  |  | CAP0002_IIF_H02 | 34547549 | Cabernet Sauvignon | Petiole | Onset of Veraison (berry softening) |
|  |  | WIN017.C21_C04 | 110360031 | Cabernet Sauvignon | Pericarp | Fruit set to maturity |
|  |  | CSECS138F09_CELu0001 | 45770915 | Cabernet Sauvignon | Cell Suspension Culture |  |
|  |  | VRJ243T7 | 38654673 | Vitis riparia | Bud | Dormant |
|  |  | WIN0528.C21_L10 | 110378264 | Cabernet Sauvignon | Flower, leaf and root | Flower, pre-anthesis; leaf, fully expanded; root, produced by air-layering |
|  |  | CAP0002_IIR_H02 | 34547632 | Cabernet Sauvignon | Petiole | Onset of Veraison (berry softening) |
|  |  | WIN0547.C21_H01 | 110385202 | Cabernet Sauvignon | Flower, leaf and root | Flower, pre-anthesis; leaf, fully expanded; root, produced by air-layering |
| *VviMAPKKK18* | GSVIVT01012116001 | EST 1321 | 56409360 | Ugni Blanc | Fruit | Green stage |
|  |  | CAbud0005_IIIF_E11 | 34546288 | Cabernet Sauvignon | Bud | Pre-bloom (10-11 days before bloom) |
|  |  | FAMU_USDA_FP_2981 | 51577122 | Vitis shuttleworthii | Entire tendril, leaves, bud, flowers | At blooming |
|  |  | CAbud0006_IF_G10 | 34545975 | Cabernet Sauvignon | Bud | Pre-bloom (10-11 days before bloom) |
|  |  | WIN1128.C21_I04 | 110420439 | Muscat Hamburg | Berries | Anthesis flower to prior to veraison |
|  |  | CAbud0006_IR_G10 | 34544983 | Cabernet Sauvignon | Bud | Pre-bloom (10-11 days before bloom) |
|  |  | WIN058.C21_J17 | 110374386 | Cabernet Sauvignon | Flower, leaf and root | Flower, pre-anthesis; leaf, fully expanded; root, produced by air-layering |
|  |  | RADIC01_000325 | 37185457 | Pinot Noir | Roots | Young roots |
|  |  | WIN106.C21_L18 | 110412351 | Muscat Hamburg | Pericarp | Fruit set to maturity |
|  |  | FAMU_USDA_FP_6549 | 51580690 | Vitis shuttleworthii | Entire tendril, leaves, bud, flowers | At blooming |
|  |  | VV_PEd06c03.b1 | 156738354 | Perlette | Bud | Young |
|  |  | VV_PEb17f06.b1 | 156730893 | Perlette | Bud | Mature |
|  |  | VV_PEb03e12.b1 | 156728587 | Perlette | Bud | Mature |
|  |  | CAP0005_IIF_H02 | 34549662 | Cabernet Sauvignon | Petiole | Onset of Veraison (berry softening) |
|  |  | S1G01458 | 110699254 | Thompson-seedless | Fruit and flower | Fruits and flowers treated with GA3 |
|  |  | VVB026B05_133902 | 27580827 | Chardonnay | Leaf | Juvenile and adult |
|  |  | CAP0005_IIR_H02 | 34549142 | Cabernet Sauvignon | Petiole | Onset of Veraison (berry softening) |
|  |  | CA48EN0001_IIIbF_C08 | 29783880 | Cabernet Sauvignon | Berries | Berry stage I |
|  |  | VVL148G08_700168 | 71890216 | Cabernet Sauvignon | Fruit with seeds removed | Mixed 36-38 - modified E-L system (Brix > 15) |
|  |  | WIN1120.C21_J19 | 110417923 | Muscat Hamburg | Berries | Anthesis flower to prior to veraison |
|  |  | VV_PEb03e12.g1 | 156728588 | Perlette | Bud | Mature |
|  |  | S7B00276 | 110715974 | Thompson-seedless | Berries | Berries 14mm with GA3 |
|  |  | VVL058F12_684654 | 71882459 | Cabernet Sauvignon | Fruit with seeds removed | Mixed 36-38 - modified E-L system (Brix > 15) |
|  |  | VV_PEd0014f05.b1 | 156736703 | Perlette | Bud | Young |
|  |  | CAB20001_IIIa_Fa_A04 | 33401438 | Cabernet Sauvignon | Flower - Bloom | Bloom |
|  |  | S2B12058 | 110703718 | Thompson-seedless | Bud |  |
|  |  | SBB04056 | 110728289 | Thompson-seedless | Inflorescence |  |
|  |  | CAB20001_IIIa_Ra_H05 | 33401569 | Cabernet Sauvignon | Flower - Bloom | Bloom |
|  |  | WIN1126.C21_A19 | 110419612 | Muscat Hamburg | Berries | Anthesis flower to prior to veraison |
|  |  | WIN0522.C21_F14 | 110376270 | Cabernet Sauvignon | Flower, leaf and root | Flower, pre-anthesis; leaf, fully expanded; root, produced by air-layering |
|  |  | EST 11346 | 32456646 | Chardonnay | Fruit without seeds | Green stage |
|  |  | BACCA01_001846 | 37185116 | Pinot Noir | Berries | Veraison |
|  |  | CAbud0006_IR_D12 | 34544955 | Cabernet Sauvignon | Bud | Pre-bloom (10-11 days before bloom) |
|  |  | CAbud0006_IF_D12 | 34545946 | Cabernet Sauvignon | Bud | Pre-bloom (10-11 days before bloom) |
|  |  | WIN1114.C21_L05 | 110416100 | Muscat Hamburg | Berries | Anthesis flower to prior to veraison |
|  |  | CA48EN0001_IIIbR_C08 | 28964887 | Cabernet Sauvignon | Berries | Berry stage I |
| *VviMAPKKK19* | GSVIVT01012632001 | SECS037G04_VERu0035 | 34363373 | Cabernet Sauvignon | Fruit with seeds removed | 35 - modified E-L system |
|  |  | VVC038B04_395207 | 30329537 | Chardonnay | Berries | Mixed; 8, 9, 11, 13, 15, 16 weeks daf |
|  |  | VVTOV95 | 160482047 | Thompson-seedless | Ovule | Mixed stages 27-48d after bloom |
|  |  | VVC038B04_140666 | 27584109 | Chardonnay | Berries | Mixed; 8, 9, 11, 13, 15, 16 weeks daf |
|  |  | VVC038B04_396595 | 30330231 | Chardonnay | Berries | Mixed; 8, 9, 11, 13, 15, 16 weeks daf |
|  |  | VVL052E07_683572 | 71881918 | Cabernet Sauvignon | Fruit with seeds removed | Mixed 36-38 - modified E-L system (Brix > 15) |
|  |  | S4B05240 | 110706755 | Thompson-seedless | Fruit | Fruits 2-3 mm |
|  |  | EST 11376 | 32457256 | Chardonnay | Fruit pedicle | Ripe Stage |
|  |  | EST 8870 | 22007381 | Cabernet Sauvignon | Fruit without seeds | Veraison |
|  |  | C3B08625 | 110692609 | Carmenere | Clusters | Clusters 4 cm |
|  |  | sT7aVVM003D20078 | 161713079 | Cabernet Sauvignon | Roots | 10 cm high plants grown in Magenta boxes |
|  |  | WIN0215.TB24_G24 | 110363703 | Cabernet Sauvignon | Flower, leaf and root | Flower, pre-anthesis; leaf, fully expanded; root, produced by air-layering |
|  |  | VVL132D10_697316 | 71888790 | Cabernet Sauvignon | Fruit with seeds removed | Mixed 36-38 - modified E-L system (Brix > 15) |
|  |  | VVB098C06_339343 | 30325650 | Chardonnay | Leaf | Juvenile and adult |
|  |  | VVB085E06_335742 | 30325045 | Chardonnay | Leaf | Juvenile and adult |
|  |  | VVB079F01_334746 | 30324547 | Chardonnay | Leaf | Juvenile and adult |
|  |  | VVB090B04_338581 | 30325269 | Chardonnay | Leaf | Juvenile and adult |
|  |  | VVB198F05_431713 | 32248404 | Chardonnay | Leaf | Juvenile and adult |
|  |  | VVB199B02_431799 | 32248447 | Chardonnay | Leaf | Juvenile and adult |
|  |  | VVB138F03_406667 | 32268221 | Chardonnay | Leaf | Juvenile and adult |
|  |  | VVB076H05_333884 | 30324116 | Chardonnay | Leaf | Juvenile and adult |
|  |  | VVB127B08_404903 | 32265449 | Chardonnay | Leaf | Juvenile and adult |
|  |  | VVB105A05_339829 | 30325893 | Chardonnay | Leaf | Juvenile and adult |
| *VviMAPKKK20* | GSVIVT01012686001 | FAMU_USDA_FP_418 | 51574559 | Vitis shuttleworthii | Entire tendril, leaves, bud, flowers | At blooming |
|  |  | WIN1125.C21_P15 | 122691960 | Muscat Hamburg | Berries | Anthesis flower to prior to veraison |
| *VviMAPKKK21* | GSVIVT01012895001 | CAB10001_IIa_Fa_E04 | 30251283 | Cabernet Sauvignon | Flower - Pre-bloom | Pre-bloom |
|  |  | CAP0005_IIF_H02 | 34549662 | Cabernet Sauvignon | Petiole | Onset of Veraison (berry softening) |
|  |  | WIN036.C21_O10 | 110365646 | Cabernet Sauvignon | Seed | Fruit set to maturity |
|  |  | CAB10001_IIa_Ra_E04 | 30251367 | Cabernet Sauvignon | Flower - Pre-bloom | Pre-bloom |
|  |  | WIN0522.C21_L16 | 110376383 | Cabernet Sauvignon | Flower, leaf and root | Flower, pre-anthesis; leaf, fully expanded; root, produced by air-layering |
|  |  | CAB30002_IIIa_Fa_C03 | 30296150 | Cabernet Sauvignon | Berries | Berry stage I |
|  |  | EST 5194 | 22011222 | Shiraz | Fruit | Ripening Stage |
|  |  | WIN063.C21_F24 | 110392330 | Cabernet Sauvignon | Seed | Fruit set to maturity |
|  |  | WIN047.C21_F15 | 110370474 | Cabernet Sauvignon | Pericarp | Fruit set to maturity |
| *VviMAPKKK22* | GSVIVT01015494001 | VV_PEb02c05.b1 | 156728369 | Perlette | Bud | Mature |
|  |  | VVB026B05_133902 | 27580827 | Chardonnay | Leaf | Juvenile and adult |
|  |  | VV_PEa016b04.b1 | 156724046 | Perlette | Bud | Mature |
|  |  | VVL058F12_684654 | 71882459 | Cabernet Sauvignon | Fruit with seeds removed | Mixed 36-38 - modified E-L system (Brix > 15) |
|  |  | WIN1127.C21_N07 | 110420214 | Muscat Hamburg | Berries | Anthesis flower to prior to veraison |
| *VviMAPKKK23* | GSVIVT01017915001 | CAB10001_Ia_Fa_D05 | 30251582 | Cabernet Sauvignon | Flower - Pre-bloom | Pre-bloom |
|  |  | CAB10001_Ia_Ra_D05 | 30251663 | Cabernet Sauvignon | Flower - Pre-bloom | Pre-bloom |
|  |  | CAB10001_IIa_Ra_A12 | 30251330 | Cabernet Sauvignon | Flower - Pre-bloom | Pre-bloom |
| *VviMAPKKK24* | GSVIVT01017968001 | VVH004G04_738945 | 71864696 | Cabernet Sauvignon | Nectary of flowers | 25 - modified E-L system |
|  |  | VVB140F12_407053 | 32268414 | Chardonnay | Leaf | Juvenile and adult |
|  |  | VVI120D08_601716 | 71875239 | Cabernet Sauvignon | Inflorescence including flowers | 12 - modified E-L system |
|  |  | SCB07424 | 110730979 | Thompson-seedless | Inflorescence | Inflorescence with GA3 |
|  |  | S1G00433 | 110698165 | Thompson-seedless | Fruit and flower | Fruits and flowers treated with GA3 |
|  |  | FAMU_USDA_FP_1043 | 51575184 | Vitis shuttleworthii | Entire tendril, leaves, bud, flowers | At blooming |
|  |  | WIN0529.C21_K19 | 110378574 | Cabernet Sauvignon | Flower, leaf and root | Flower, pre-anthesis; leaf, fully expanded; root, produced by air-layering |
|  |  | S6B01936 | 110711559 | Thompson-seedless | Fruit | Fruits 7-9 mm |
|  |  | WIN0542.C21_A09 | 110383200 | Cabernet Sauvignon | Flower, leaf and root | Flower, pre-anthesis; leaf, fully expanded; root, produced by air-layering |
|  |  | WIN0414.C21_F15 | 110367801 | Cabernet Sauvignon | Pericarp | Fruit set to maturity |
|  |  | CSECS215G05_5_PREn0028 | 87586588 | Cabernet Sauvignon | Fruit | 28 - modified E-L system |
|  |  | C2B01079 | 110686935 | Carmenere | Bud - cluster |  |
|  |  | WIN1149.C21_K09 | 110427426 | Muscat Hamburg | Berries | Anthesis flower to prior to veraison |
|  |  | WIN0212.TB24_C10 | 110362843 | Cabernet Sauvignon | Flower, leaf and root | Flower, pre-anthesis; leaf, fully expanded; root, produced by air-layering |
|  |  | WIN0214.TB24_P01 | 110363586 | Cabernet Sauvignon | Flower, leaf and root | Flower, pre-anthesis; leaf, fully expanded; root, produced by air-layering |
|  |  | VVI062F04_592206 | 71872128 | Cabernet Sauvignon | Inflorescence including flowers | 12 - modified E-L system |
|  |  | GEMMA01_000225 | 37187475 | Pinot Noir | Bud | Bud swelling |
| *VviMAPKKK25* | GSVIVT01018020001 | WIN0548.C21_K03 | 110385552 | Cabernet Sauvignon | Flower, leaf and root | Flower, pre-anthesis; leaf, fully expanded; root, produced by air-layering |
|  |  | sT7aVVM022D04014 | 161718070 | Cabernet Sauvignon | Roots | 10 cm high plants grown in Magenta boxes |
|  |  | sT7aVVM007G04010 | 161714587 | Cabernet Sauvignon | Roots | 10 cm high plants grown in Magenta boxes |
|  |  | sT7aVVM021H15058 | 161720040 | Cabernet Sauvignon | Roots | 10 cm high plants grown in Magenta boxes |
|  |  | sT7aVVM005E01011 | 161713698 | Cabernet Sauvignon | Roots | 10 cm high plants grown in Magenta boxes |
|  |  | sT7aVVM_AER11E05 | 161705879 | Cabernet Sauvignon | Roots | 10 cm high plants grown in Magenta boxes |
|  |  | sT7aVVM_AER11E05-2 | 161777284 | Cabernet Sauvignon | Roots | 10 cm high plants grown in Magenta boxes |
|  |  | WIN0557.C21_A12 | 110388151 | Cabernet Sauvignon | Flower, leaf and root | Flower, pre-anthesis; leaf, fully expanded; root, produced by air-layering |
|  |  | WIN0546.C21_D04 | 110384825 | Cabernet Sauvignon | Flower, leaf and root | Flower, pre-anthesis; leaf, fully expanded; root, produced by air-layering |
|  |  | WIN1128.C21_E17 | 110420368 | Muscat Hamburg | Berries | Anthesis flower to prior to veraison |
|  |  | VVG049H04_760725 | 71858850 | Cabernet Sauvignon | Cell Suspension Culture |  |
|  |  | CSECS001F08_PREu0032 | 34361579 | Cabernet Sauvignon | Fruit with seeds removed | 32 - modified E-L system |
|  |  | CSECS057D04_VERu0035 | 34364088 | Cabernet Sauvignon | Fruit with seeds removed | 35 - modified E-L system |
|  |  | VVC048G12_417221 | 32246258 | Chardonnay | Berries | Mixed; 8, 9, 11, 13, 15, 16 weeks daf |
|  |  | VVC048G12_416057 | 32245676 | Chardonnay | Berries | Mixed; 8, 9, 11, 13, 15, 16 weeks daf |
|  |  | C4B06976 | 110697646 | Carmenere | Clusters | Veraison |
| *VviMAPKKK26* | GSVIVT01018052001 | VVG004F02_755811 | 71856393 | Cabernet Sauvignon | Cell Suspension Culture |  |
|  |  | VVC035G08_139390 | 27583915 | Chardonnay | Berries | Mixed; 8, 9, 11, 13, 15, 16 weeks daf |
|  |  | VVA004F05_52521 | 18458191 | Chardonnay | Leaf | Juvenile and adult |
|  |  | C2B06519 | 110690328 | Carmenere | Bud - cluster |  |
|  |  | WIN1124.C21_F23 | 110419083 | Muscat Hamburg | Berries | Anthesis flower to prior to veraison |
|  |  | VVL085F03_689188 | 71884726 | Cabernet Sauvignon | Fruit with seeds removed | Mixed 36-38 - modified E-L system (Brix > 15) |
|  |  | S2B11796 | 110701353 | Thompson-seedless | Bud |  |
|  |  | VVL100F02_691760 | 71886012 | Cabernet Sauvignon | Fruit with seeds removed | Mixed 36-38 - modified E-L system (Brix > 15) |
|  |  | VVC017H11_416649 | 32245972 | Chardonnay | Berries | Mixed; 8, 9, 11, 13, 15, 16 weeks daf |
|  |  | VVC017H11_415689 | 32245492 | Chardonnay | Berries | Mixed; 8, 9, 11, 13, 15, 16 weeks daf |
|  |  | sT7aVVM022E13059 | 161718798 | Cabernet Sauvignon | Roots | 10 cm high plants grown in Magenta boxes |
|  |  | VVL079G10_688236 | 71884250 | Cabernet Sauvignon | Fruit with seeds removed | Mixed 36-38 - modified E-L system (Brix > 15) |
|  |  | VVL079C11_688168 | 71884216 | Cabernet Sauvignon | Fruit with seeds removed | Mixed 36-38 - modified E-L system (Brix > 15) |
|  |  | WIN0214.TB24_N04 | 110363558 | Cabernet Sauvignon | Flower, leaf and root | Flower, pre-anthesis; leaf, fully expanded; root, produced by air-layering |
|  |  | VV_PEd0012c09.b1 | 156736255 | Perlette | Bud | Young |
|  |  | VV_PEd0012c10.g1 | 156736258 | Perlette | Bud | Young |
|  |  | VV_PEb05h11.g1 | 156728977 | Perlette | Bud | Mature |
|  |  | VV_PEb05h11.b1 | 156728976 | Perlette | Bud | Mature |
|  |  | WIN1113.C21.1_O06 | 110415853 | Muscat Hamburg | Berries | Anthesis flower to prior to veraison |
|  |  | WIN017.C21_F03 | 110358405 | Cabernet Sauvignon | Pericarp | Fruit set to maturity |
|  |  | VVD069H04_353309 | 30136000 | Chardonnay | Berries | Mixed; 8, 9, 11, 13, 15, 16 weeks daf |
|  |  | C1G07356 | 110685297 | Carmenere | Fruit - bud - clusters |  |
| *VviMAPKKK27* | GSVIVT01019010001 | - | - | - | - | - |
| *VviMAPKKK28* | GSVIVT01019630001 | sT7aVVM_AER52D10 | 161711463 | Cabernet Sauvignon | Roots | 10 cm high plants grown in Magenta boxes |
|  |  | C3B08625 | 110692609 | Carmenere | Clusters | Clusters 4 cm |
|  |  | VVB140F12_407053 | 32268414 | Chardonnay | Leaf | Juvenile and adult |
|  |  | VV_PEb09b04.b1 | 156729489 | Perlette | Bud | Mature |
|  |  | VVL068B09_686294 | 71883279 | Cabernet Sauvignon | Fruit with seeds removed | Mixed 36-38 - modified E-L system (Brix > 15) |
|  |  | WIN1146.C21_N18 | 110426507 | Muscat Hamburg | Berries | Anthesis flower to prior to veraison |
|  |  | WIN0549.C21_L07 | 122690286 | Cabernet Sauvignon | Flower, leaf and root | Flower, pre-anthesis; leaf, fully expanded; root, produced by air-layering |
|  |  | S1G00433 | 110698165 | Thompson-seedless | Fruit and flower | Fruits and flowers treated with GA3 |
|  |  | WIN028.TB24.1_P01 | 110361960 | Cabernet Sauvignon | Flower, leaf and root | Flower, pre-anthesis; leaf, fully expanded; root, produced by air-layering |
|  |  | VVI014H09_585158 | 71868604 | Cabernet Sauvignon | Inflorescence including flowers | 12 - modified E-L system |
| *VviMAPKKK29* | GSVIVT01019739001 | VVC038E01_140724 | 27584138 | Chardonnay | Berries | Mixed; 8, 9, 11, 13, 15, 16 weeks daf |
|  |  | VVC030A08_138404 | 27583422 | Chardonnay | Berries | Mixed; 8, 9, 11, 13, 15, 16 weeks daf |
|  |  | S1G05941 | 110699701 | Thompson-seedless | Fruit and flower | Fruits and flowers treated with GA3 |
|  |  | VVD071B06_353515 | 30136103 | Chardonnay | Berries | Mixed; 8, 9, 11, 13, 15, 16 weeks daf |
|  |  | WIN0549.C21_N15 | 110385948 | Cabernet Sauvignon | Flower, leaf and root | Flower, pre-anthesis; leaf, fully expanded; root, produced by air-layering |
|  |  | VV_PEd0015g07.b1 | 156736923 | Perlette | Bud | Young |
| *VviMAPKKK30* | GSVIVT01019821001 | - | - | - | - | - |
| *VviMAPKKK31* | GSVIVT01020712001 | sT7aVVM_AER70D09 | 161708715 | Cabernet Sauvignon | Roots | 10 cm high plants grown in Magenta boxes |
|  |  | INFIO01_000753 | 37190575 | Regent | Inflorescence | Young inflorescence before flowering |
|  |  | CAB10003_IVa_Fa_A10 | 30252707 | Cabernet Sauvignon | Flower - Pre-bloom | Pre-bloom |
|  |  | RR890915I0005_IIa_Fa_D12 | 33399984 | Vitis hybrid cultivar | Leaf |  |
|  |  | CAB20002_Ib_Fb_H11 | 33402554 | Cabernet Sauvignon | Flower - Bloom | Bloom |
|  |  | FAMU_USDA_FP_5256 | 51579397 | Vitis shuttleworthii | Entire tendril, leaves, bud, flowers | At blooming |
|  |  | WIN0524.C21_E12 | 110376889 | Cabernet Sauvignon | Flower, leaf and root | Flower, pre-anthesis; leaf, fully expanded; root, produced by air-layering |
|  |  | WIN057.C21_P14 | 110382247 | Cabernet Sauvignon | Flower, leaf and root | Flower, pre-anthesis; leaf, fully expanded; root, produced by air-layering |
|  |  | WIN0537.C21_E01 | 110380652 | Cabernet Sauvignon | Flower, leaf and root | Flower, pre-anthesis; leaf, fully expanded; root, produced by air-layering |
|  |  | WIN0820.C21_A06 | 122691011 | Cabernet Sauvignon | Seed | Fruit set to maturity |
|  |  | WIN103.C21_F01 | 110405440 | Muscat Hamburg | Pericarp | Fruit set to maturity |
|  |  | WIN1141.C21_O23 | 110424859 | Muscat Hamburg | Berries | Anthesis flower to prior to veraison |
|  |  | WIN0548.C21_I13 | 110385519 | Cabernet Sauvignon | Flower, leaf and root | Flower, pre-anthesis; leaf, fully expanded; root, produced by air-layering |
|  |  | WIN084.C21_O01 | 110395215 | Cabernet Sauvignon | Seed | Fruit set to maturity |
|  |  | IN094.C21_M07 | 110404701 | Cabernet Sauvignon | Pericarp | Fruit set to maturity |
| *VviMAPKKK32* | GSVIVT01021854001 | WIN0510.C21_C10 | 110371589 | Cabernet Sauvignon | Flower, leaf and root | Flower, pre-anthesis; leaf, fully expanded; root, produced by air-layering |
|  |  | S6B01936 | 110711559 | Thompson-seedless | Fruit | Fruits 7-9 mm |
|  |  | CAB70003_IIaR_B10 | 30304059 | Cabernet Sauvignon | Berries | Post-Veraison, 18-19 brix |
|  |  | EST 17604 | 46910285 | Cabernet Sauvignon | Fruit skin | Ripening Stage |
|  |  | VVB140F12_407053 | 32268414 | Chardonnay | Leaf | Juvenile and adult |
|  |  | S1G00433 | 110698165 | Thompson-seedless | Fruit and flower | Fruits and flowers treated with GA3 |
|  |  | CAB70003_IIaF_B10 | 30303995 | Cabernet Sauvignon | Berries | Post-Veraison, 18-19 brix |
|  |  | WIN1015.C21_I16 | 110407564 | Muscat Hamburg | Pericarp | Fruit set to maturity |
|  |  | VVH004G04_738945 | 71864696 | Cabernet Sauvignon | Nectary of flowers | 25 - modified E-L system |
|  |  | CSECS198F04_5_PREn0028 | 87584363 | Cabernet Sauvignon | Fruit | 28 - modified E-L system |
|  |  | CSECS198F04_PREn0028 | 83276489 | Cabernet Sauvignon | Fruit | 28 - modified E-L system |
|  |  | WIN0547.C21_I11 | 110385228 | Cabernet Sauvignon | Flower, leaf and root | Flower, pre-anthesis; leaf, fully expanded; root, produced by air-layering |
|  |  | GEMMA01_000673 | 37187852 | Pinot Noir | Bud | Bud swelling |
|  |  | CAB10006_IIIa_Fa_F08 | 30255720 | Cabernet Sauvignon | Flower - Pre-bloom | Pre-bloom |
| *VviMAPKKK33* | GSVIVT01021884001 | WIN085.C21_A12 | 110397588 | Cabernet Sauvignon | Seed | Fruit set to maturity |
|  |  | CAB70001_IaF_D11 | 30303225 | Cabernet Sauvignon | Berries | Post-Veraison, 18-19 brix |
|  |  | SCB06047 | 110730765 | Thompson-seedless | Inflorescence | Inflorescence with GA3 |
|  |  | S2B24502 | 110702451 | Thompson-seedless | Bud |  |
|  |  | CAB70001_IaR_D11 | 30303291 | Cabernet Sauvignon | Berries | Post-Veraison, 18-19 brix |
|  |  | CAB70003_IIIaF_C03 | 30303866 | Cabernet Sauvignon | Berries | Post-Veraison, 18-19 brix |
|  |  | CAB70003_IIIaR_C03 | 30303928 | Cabernet Sauvignon | Berries | Post-Veraison, 18-19 brix |
|  |  | FAMU_USDA_FP_2392 | 51576533 | Vitis shuttleworthii | Entire tendril, leaves, bud, flowers | At blooming |
| *VviMAPKKK34* | GSVIVT01022098001 | sT7aVVM_AER9C07 | 161709981 | Cabernet Sauvignon | Roots | 10 cm high plants grown in Magenta boxes |
|  |  | C1G05824 | 110685217 | Carmenere | Fruit - bud - clusters |  |
|  |  | VVC032E03_138834 | 27583637 | Chardonnay | Berries | Mixed; 8, 9, 11, 13, 15, 16 weeks daf |
|  |  | VVC032E03_395017 | 30329442 | Chardonnay | Berries | Mixed; 8, 9, 11, 13, 15, 16 weeks daf |
|  |  | WIN1125.C21_F14 | 110419396 | Muscat Hamburg | Berries | Anthesis flower to prior to veraison |
|  |  | VVD026E12_370693 | 30131177 | Chardonnay | Berries | Mixed; 8, 9, 11, 13, 15, 16 weeks daf |
|  |  | VVI118B05_601336 | 71875049 | Cabernet Sauvignon | Inflorescence including flowers | 12 - modified E-L system |
|  |  | S2B20838 | 110700417 | Thompson-seedless | Bud |  |
|  |  | VVC032E03_396143 | 30330005 | Chardonnay | Berries | Mixed; 8, 9, 11, 13, 15, 16 weeks daf |
|  |  | VVD035A07_347743 | 30133216 | Chardonnay | Berries | Mixed; 8, 9, 11, 13, 15, 16 weeks daf |
|  |  | VVC029D11_394935 | 30329401 | Chardonnay | Berries | Mixed; 8, 9, 11, 13, 15, 16 weeks daf |
| *VviMAPKKK35* | GSVIVT01022115001 | WIN0573.C21_B02 | 122690686 | Cabernet Sauvignon | Flower, leaf and root | Flower, pre-anthesis; leaf, fully expanded; root, produced by air-layering |
|  |  | WIN0538.C21_D24 | 110380973 | Cabernet Sauvignon | Flower, leaf and root | Flower, pre-anthesis; leaf, fully expanded; root, produced by air-layering |
|  |  | WIN0511.C21.1_P22 | 122689266 | Cabernet Sauvignon | Flower, leaf and root | Flower, pre-anthesis; leaf, fully expanded; root, produced by air-layering |
|  |  | VVH036G12_744717 | 71860391 | Cabernet Sauvignon | Nectary of flowers | 25 - modified E-L system |
|  |  | USDA_FP_131387 | 47090031 | Vitis shuttleworthii | Entire tendril, leaves, bud, flowers | At blooming |
|  |  | FAMU_USDA_FP_3073 | 51577214 | Vitis shuttleworthii | Entire tendril, leaves, bud, flowers | At blooming |
| *VviMAPKKK36* | GSVIVT01022116001 | sT7aVVM011G02009 | 161716439 | Cabernet Sauvignon | Roots | 10 cm high plants grown in Magenta boxes |
|  |  | CGF1000634_D09 | 33407474 | Cabernet Sauvignon | Stem | Pre-bloom (10-11 days before bloom) |
|  |  | CGF1000633_D09 | 33407551 | Cabernet Sauvignon | Stem | Pre-bloom (10-11 days before bloom) |
|  |  | USDA_FP_132392 | 47091036 | Vitis shuttleworthii | Entire tendril, leaves, bud, flowers | At blooming |
|  |  | VVL108C07_693100 | 71886682 | Cabernet Sauvignon | Fruit with seeds removed | Mixed 36-38 - modified E-L system (Brix > 15) |
|  |  | VVD061D12_351175 | 30134933 | Chardonnay | Berries | Mixed; 8, 9, 11, 13, 15, 16 weeks daf |
| *VviMAPKKK37* | GSVIVT01023037001 | VV_PEc02B02.f.ab1 | 156733147 | Perlette | Bud | Mature |
|  |  | VV_PEc02B02.r.ab1 | 156733155 | Perlette | Bud | Mature |
|  |  | CAB40006_IVa_Ra_G09 | 30302119 | Cabernet Sauvignon | Berries | Berry on stage II, 9 mm |
|  |  | VVI033F12_588194 | 71870122 | Cabernet Sauvignon | Inflorescence including flowers | 12 - modified E-L system |
|  |  | CAB40006_IVa_Fa_G09 | 30302049 | Cabernet Sauvignon | Berries | Berry on stage II, 9 mm |
| *VviMAPKKK38* | GSVIVT01023048001 | - | - | - | - | - |
| *VviMAPKKK39* | GSVIVT01023216001 | - | - | - | - | - |
| *VviMAPKKK40* | GSVIVT01023958001 | EST 235 | 14580409 | Shiraz | Berries | Veraison |
|  |  | sT7aVVM_AER30C05 | 161708954 | Cabernet Sauvignon | Roots | 10 cm high plants grown in Magenta boxes |
|  |  | WIN0558.C21_G03 | 110388567 | Cabernet Sauvignon | Flower, leaf and root | Flower, pre-anthesis; leaf, fully expanded; root, produced by air-layering |
|  |  | EST 15424 | 46918106 | Shiraz | Fruit without seeds | Veraison |
|  |  | CSECS169E04_POSu0038 | 83275700 | Cabernet Sauvignon | Fruit with seeds removed | 38 - modified E-L system |
|  |  | WIN1131.C21_M09 | 110421497 | Muscat Hamburg | Berries | Anthesis flower to prior to veraison |
|  |  | L12_94_Sh_CT_P2_A05.ab1 1 537 | 134030613 | Vitis arizonica x Vitis rupestris | Shoot | Vegetative stage-control |
|  |  | VVA003H11_392023 | 30320953 | Chardonnay | Leaf | Juvenile and adult |
|  |  | VVA003H11_52437 | 18458149 | Chardonnay | Leaf | Juvenile and adult |
| *VviMAPKKK41* | GSVIVT01024578001 | CAP0007_IIIR_A08 | 34550453 | Cabernet Sauvignon | Petiole | Onset of Veraison (berry softening) |
|  |  | VVB014D12_131324 | 27579538 | Chardonnay | Leaf | Juvenile and adult |
|  |  | RR890915N0001_IIId_Rd_G08 | 33395059 | Vitis hybrid cultivar | Leaf |  |
|  |  | S6B01936 | 110711559 | Thompson-seedless | Fruit | Fruits 7-9 mm |
|  |  | R890915N0001_IId_Rc_G08 | 33395157 | Vitis hybrid cultivar | Leaf |  |
|  |  | RR890915N0001_IIc_Fc_G08 | 33395114 | Vitis hybrid cultivar | Leaf |  |
|  |  | VVD081H03_355207 | 30136949 | Chardonnay | Berries | Mixed; 8, 9, 11, 13, 15, 16 weeks daf |
|  |  | VVB140F12_407053 | 32268414 | Chardonnay | Leaf | Juvenile and adult |
|  |  | S1G00433 | 110698165 | Thompson-seedless | Fruit and flower | Fruits and flowers treated with GA3 |
|  |  | VVD003H08_127428 | 27586936 | Chardonnay | Berries | Mixed; 8, 9, 11, 13, 15, 16 weeks daf |
|  |  | VVD105F12_369701 | 30130681 | Chardonnay | Berries | Mixed; 8, 9, 11, 13, 15, 16 weeks daf |
|  |  | sT7aVVM021C04014 | 161718633 | Cabernet Sauvignon | Roots | 10 cm high plants grown in Magenta boxes |
|  |  | VVD003H08_397291 | 30330580 | Chardonnay | Berries | Mixed; 8, 9, 11, 13, 15, 16 weeks daf |
|  |  | VVB014D12_393055 | 30328462 | Chardonnay | Leaf | Juvenile and adult |
|  |  | CAP0007_IIIF_A08 | 34550373 | Cabernet Sauvignon | Petiole | Onset of Veraison (berry softening) |
|  |  | VVD023A05_345787 | 30132238 | Chardonnay | Berries | Mixed; 8, 9, 11, 13, 15, 16 weeks daf |
|  |  | VVD163B07_376469 | 30127867 | Chardonnay | Berries | Mixed; 8, 9, 11, 13, 15, 16 weeks daf |
|  |  | BACCA01_000868 | 37184219 | Pinot Noir | Berries | Veraison |
|  |  | BACCA01_000883 | 37184233 | Pinot Noir | Berries | Veraison |
| *VviMAPKKK42* | GSVIVT01026487001 | sT7aVVM010O06017 | 161718297 | Cabernet Sauvignon | Roots | 10 cm high plants grown in Magenta boxes |
|  |  | S1G05421 | 110698751 | Thompson-seedless | Fruit and flower | Fruits and flowers treated with GA3 |
|  |  | WIN0819.C21_H04 | 110399396 | Cabernet Sauvignon | Seed | Fruit set to maturity |
|  |  | sT7aVVM025K02005 | 161721327 | Cabernet Sauvignon | Roots | 10 cm high plants grown in Magenta boxes |
|  |  | sT7aVVM022H24090 | 161719911 | Cabernet Sauvignon | Roots | 10 cm high plants grown in Magenta boxes |
|  |  | WIN045.C21_E24 | 110365716 | Cabernet Sauvignon | Petiole | Fruit set to maturity |
|  |  | sT7aVVM007E24092 | 161714297 | Cabernet Sauvignon | Roots | 10 cm high plants grown in Magenta boxes |
|  |  | VVC030A08_138404 | 27583422 | Chardonnay | Berries | Mixed; 8, 9, 11, 13, 15, 16 weeks daf |
|  |  | VVC030A08_396551 | 30330209 | Chardonnay | Berries | Mixed; 8, 9, 11, 13, 15, 16 weeks daf |
|  |  | FAMU_USDA_FP_4327 | 51578468 | Vitis shuttleworthii | Entire tendril, leaves, bud, flowers | At blooming |
|  |  | VVH026B10_742819 | 71863025 | Cabernet Sauvignon | Nectary of flowers | 25 - modified E-L system |
|  |  | CA12LIO3IIFb_B02 | 26257306 | Cabernet Sauvignon | Leaf | Late season sample |
|  |  | WIN025.TB24.1_F04 | 110360965 | Cabernet Sauvignon | Flower, leaf and root | Flower, pre-anthesis; leaf, fully expanded; root, produced by air-layering |
|  |  | CA12LIO3IIbF_B02 | 26257410 | Cabernet Sauvignon | Leaf | Late season sample |
|  |  | VVB135F10_406119 | 32267947 | Chardonnay | Leaf | Juvenile and adult |
|  |  | VV_PEb15a05.b1 | 156730456 | Perlette | Bud | Mature |
|  |  | VV_PEa20d09.b1 | 156727249 | Perlette | Bud | Mature |
|  |  | VV_PEa20d09.g1 | 156727250 | Perlette | Bud | Mature |
|  |  | SCB07505 | 110732912 | Thompson-seedless | Inflorescence | Inflorescence with GA3 |
|  |  | WIN026.TB24.1_N14 | 110361379 | Cabernet Sauvignon | Flower, leaf and root | Flower, pre-anthesis; leaf, fully expanded; root, produced by air-layering |
|  |  | WIN1033.C21_F02 | 110411320 | Muscat Hamburg | Pericarp | Fruit set to maturity |
|  |  | SCB04920 | 110730359 | Thompson-seedless | Inflorescence | Inflorescence with GA3 |
|  |  | VV_PEb11h07.g1 | 156729947 | Perlette | Bud | Mature |
|  |  | CA12LIO3IIRb_B02 | 26258196 | Cabernet Sauvignon | Leaf | Late season sample |
|  |  | VV_PEb10f10.g1 | 156729747 | Perlette | Bud | Mature |
|  |  | CA48LN09IIIR-A12 | 26263206 | Cabernet Sauvignon | Leaf | Late season sample |
|  |  | CA48LN09IIIF-B9 | 26262294 | Cabernet Sauvignon | Leaf | Late season sample |
|  |  | VVC030A08_394945 | 30329406 | Chardonnay | Berries | Mixed; 8, 9, 11, 13, 15, 16 weeks daf |
|  |  | CAP0003_IVF_G03 | 34548171 | Cabernet Sauvignon | Petiole | Onset of Veraison (berry softening) |
| *VviMAPKKK43* | GSVIVT01026546001 | VVH004G04_738945 | 71864696 | Cabernet Sauvignon | Nectary of flowers | 25 - modified E-L system |
|  |  | VV_PEb03c06.b1 | 156728535 | Perlette | Bud | Mature |
|  |  | CAB20001_IVa_Ra_D05 | 33401846 | Cabernet Sauvignon | Flower - Bloom | Bloom |
|  |  | CAB70003_IIaF_E10 | 30304019 | Cabernet Sauvignon | Berries | Post-Veraison, 18-19 brix |
|  |  | S4B05240 | 110706755 | Thompson-seedless | Fruit | Fruits 7-9 mm |
|  |  | sT7aVVM003D20078 | 161713079 | Cabernet Sauvignon | Roots | 10 cm high plants grown in Magenta boxes |
|  |  | CAB20001_IVa_Fa_D05 | 33401767 | Cabernet Sauvignon | Flower - Bloom | Bloom |
| *VviMAPKKK44* | GSVIVT01027189001 | SCB06839 | 110733037 | Thompson-seedless | Inflorescence | Inflorescence with GA3 |
|  |  | CAbud0003_IVF_H02 | 34544416 | Cabernet Sauvignon | Bud | Pre-bloom (10-11 days before bloom) |
|  |  | S4B05240 | 110706755 | Thompson-seedless | Fruit | Fruit 2-3 mm |
|  |  | VV_PEb09b04.b1 | 156729489 | Perlette | Bud | Mature |
|  |  | WIN0549.C21_L07 | 122690286 | Cabernet Sauvignon | Flower, leaf and root | Flower, pre-anthesis; leaf, fully expanded; root, produced by air-layering |
| *VviMAPKKK45* | GSVIVT01028897001 | VVD156E10_379551 | 30125481 | Chardonnay | Berries | Mixed; 8, 9, 11, 13, 15, 16 weeks daf |
|  |  | VV_PEa01a04.b2 | 156724202 | Perlette | Bud | Mature |
|  |  | VVC038E01_140724 | 27584138 | Chardonnay | Berries | Mixed; 8, 9, 11, 13, 15, 16 weeks daf |
|  |  | VVH026B10_742819 | 71863025 | Cabernet Sauvignon | Nectary of flowers | 25 - modified E-L system |
|  |  | WIN0535.C21_F09 | 122689867 | Cabernet Sauvignon | Flower, leaf and root | Flower, pre-anthesis; leaf, fully expanded; root, produced by air-layering |
|  |  | S1G05941 | 110699701 | Thompson-seedless | Fruit and flower | Fruits and flowers treated with GA3 |
|  |  | VVC030A08_138404 | 27583422 | Chardonnay | Berries | Mixed; 8, 9, 11, 13, 15, 16 weeks daf |
| *VviMAPKKK46* | GSVIVT01029055001 | CAbud0006_IF_G10 | 34545975 | Cabernet Sauvignon | Bud | Pre-bloom (10-11 days before bloom) |
|  |  | CAB20001_IIIa_Fa_A04 | 33401438 | Cabernet Sauvignon | Flower - Bloom | Bloom |
|  |  | WIN063.C21_F24 | 110392330 | Cabernet Sauvignon | Seed | Fruit set to maturity |
|  |  | RR890915N0001_IIc_Fc_C04 | 33395085 | Vitis hybrid cultivar | Leaf |  |
|  |  | WIN1126.C21_A19 | 110419612 | Muscat Hamburg | Berries | Anthesis flower to prior to veraison |
| *VviMAPKKK47* | GSVIVT01029147001 | VV_PEa016b04.b1 | 156724046 | Perlette | Bud | Mature |
|  |  | CAB30002_IIIa_Fa_C03 | 30296150 | Cabernet Sauvignon | Berries | Berry stage I |
|  |  | FAMU_USDA_FP_426 | 51574567 | Vitis shuttleworthii | Entire tendril, leaves, bud, flowers | At blooming |
|  |  | VVL058F12_684654 | 71882459 | Cabernet Sauvignon | Fruit with seeds removed | Mixed 36-38 - modified E-L system (Brix > 15) |
|  |  | VVL051F10_683430 | 71881847 | Cabernet Sauvignon | Fruit with seeds removed | Mixed 36-38 - modified E-L system (Brix > 15) |
|  |  | VV_PEb02c05.b1 | 156728369 | Perlette | Bud | Mature |
|  |  | VV_PEb02c05.g1 | 156728370 | Perlette | Bud | Mature |
|  |  | CAB10001_IIa_Ra_E04 | 30251367 | Cabernet Sauvignon | Flower - Pre-bloom | Pre-bloom |
|  |  | VVB048F10_325464 | 30322193 | Chardonnay | Leaf | Juvenile and adult |
|  |  | CAB30002_IIIc_Rc_C03 | 30296210 | Cabernet Sauvignon | Berries | Berry stage I |
| *VviMAPKKK48* | GSVIVT01029426001 | sT7aVVM003D20078 | 161713079 | Cabernet Sauvignon | Roots | 10 cm high plants grown in Magenta boxes |
|  |  | VVB140F12_407053 | 32268414 | Chardonnay | Leaf | Juvenile and adult |
|  |  | VVL068B09_686294 | 71883279 | Cabernet Sauvignon | Fruit with seeds removed | Mixed 36-38 - modified E-L system (Brix > 15) |
|  |  | WIN028.TB24.1_P01 | 110361960 | Cabernet Sauvignon | Flower, leaf and root | Flower, pre-anthesis; leaf, fully expanded; root, produced by air-layering |
|  |  | S1G00433 | 110698165 | Thompson-seedless | Fruit and flower | Fruits and flowers treated with GA3 |
|  |  | WIN0549.C21_L07 | 122690286 | Cabernet Sauvignon | Flower, leaf and root | Flower, pre-anthesis; leaf, fully expanded; root, produced by air-layering |
| *VviMAPKKK49* | GSVIVT01030044001 | sT7aVVM_AER55D05 | 161709407 | Cabernet Sauvignon | Roots | 10 cm high plants grown in Magenta boxes |
|  |  | sT7aVVM018F24092 | 161718416 | Cabernet Sauvignon | Roots | 10 cm high plants grown in Magenta boxes |
|  |  | sT7aVVM_AER92A03 | 161707589 | Cabernet Sauvignon | Roots | 10 cm high plants grown in Magenta boxes |
|  |  | VVB119A04_383043 | 30327823 | Chardonnay | Leaf | Juvenile and adult |
|  |  | VVB177G08_413777 | 32271776 | Chardonnay | Leaf | Juvenile and adult |
|  |  | VVB140F12_407053 | 32268414 | Chardonnay | Leaf | Juvenile and adult |
|  |  | VVB011H04_403937 | 32246976 | Chardonnay | Leaf | Juvenile and adult |
|  |  | VVB180G03_414293 | 32272034 | Chardonnay | Leaf | Juvenile and adult |
|  |  | VVB035B02_134934 | 27581343 | Chardonnay | Leaf | Juvenile and adult |
|  |  | VVB125D04_404573 | 32265284 | Chardonnay | Leaf | Juvenile and adult |
|  |  | VVB011H04_403233 | 32246624 | Chardonnay | Leaf | Juvenile and adult |
|  |  | VVB141H10_407281 | 32268528 | Chardonnay | Leaf | Juvenile and adult |
|  |  | VVD002H09_127256 | 27586850 | Chardonnay | Berries | Mixed; 8, 9, 11, 13, 15, 16 weeks daf |
|  |  | VVD010G10_343801 | 30128934 | Chardonnay | Berries | Mixed; 8, 9, 11, 13, 15, 16 weeks daf |
|  |  | VV_PEb23a09.b1 | 156731780 | Perlette | Bud | Mature |
|  |  | VV_PEb23a09.g1 | 156731781 | Perlette | Bud | Mature |
|  |  | VV_PEb03c06.b1 | 156728535 | Perlette | Bud | Mature |
|  |  | FAMU_USDA_FP_6507 | 51580648 | Vitis shuttleworthii | Entire tendril, leaves, bud, flowers | At blooming |
|  |  | FAMU_USDA_FP_4783 | 51578924 | Vitis shuttleworthii | Entire tendril, leaves, bud, flowers | At blooming |
|  |  | USDA_FP_132471 | 47091115 | Vitis shuttleworthii | Entire tendril, leaves, bud, flowers | At blooming |
|  |  | FAMU_USDA_FP_814 | 51574955 | Vitis shuttleworthii | Entire tendril, leaves, bud, flowers | At blooming |
|  |  | CA32EN0004_IaF_F06 | 29785898 | Cabernet Sauvignon | Leaf | Mid-season leaf material |
|  |  | CA32EN0004_IaR_F06 | 29785937 | Cabernet Sauvignon | Leaf | Mid-season leaf material |
|  |  | WIN031.C21_B06 | 110364507 | Cabernet Sauvignon | Seed | Fruit set to maturity |
|  |  | WIN057.C21_A14 | 110382132 | Cabernet Sauvignon | Flower, leaf and root | Flower, pre-anthesis; leaf, fully expanded; root, produced by air-layering |
|  |  | CSECS004B05_PREU0032 | 34361690 | Cabernet Sauvignon | Fruit with seeds removed | 32 - modified E-L system |
| *VviMAPKKK50* | GSVIVT01030194001 | CAbud0006_IIIF_G03 | 34546134 | Cabernet Sauvignon | Bud | Pre-bloom (10-11 days before bloom) |
|  |  | CAbud0006_IIIR_G03 | 34545140 | Cabernet Sauvignon | Bud | Pre-bloom (10-11 days before bloom) |
|  |  | VV_PEd0017g02.g1 | 156737298 | Perlette | Bud | Young |
|  |  | VV_PEd0017g02.b1 | 156737297 | Perlette | Bud | Young |
|  |  | CA12LIO2IIFb_H06 | 26257121 | Cabernet Sauvignon | Leaf | Late season sample |
|  |  | BACCA01_000826 | 37184178 | Pinot Noir | Berries | Veraison |
|  |  | WIN117.C21_D22 | 110429607 | Muscat Hamburg | Berries | Anthesis flower to prior to veraison |
|  |  | S1G00870 | 110698669 | Thompson-seedless | Fruit and flower | Fruits and flowers treated with GA3 |
| *VviMAPKKK51* | GSVIVT01030202001 | S4B05240 | 110706755 | Thompson-seedless | Fruit | Fruit 2-3 mm |
|  |  | VV_PEb09b04.b1 | 156729489 | Perlette | Bud | Mature |
|  |  | VVB014D12_131324 | 27579538 | Chardonnay | Leaf | Juvenile and adult |
|  |  | VVD105F12_369701 | 30130681 | Chardonnay | Berries | Mixed; 8, 9, 11, 13, 15, 16 weeks daf |
| *VviMAPKKK52* | GSVIVT01031721001 | VV_PEd19g08.b1 | 156739036 | Perlette | Bud | Young |
|  |  | VV_PEd19g08.g1 | 156739045 | Perlette | Bud | Young |
|  |  | WIN1125.C21_F14 | 110419396 | Muscat Hamburg | Berries | Anthesis flower to prior to veraison |
|  |  | VVD142A10_374349 | 30126890 | Chardonnay | Berries | Mixed; 8, 9, 11, 13, 15, 16 weeks daf |
|  |  | WIN0511.C21.1_P22 | 122689266 | Cabernet Sauvignon | Flower, leaf and root | Flower, pre-anthesis; leaf, fully expanded; root, produced by air-layering |
| *VviMAPKKK53* | GSVIVT01032232001 | VV_PEb19c02.g1 | 156732745 | Perlette | Bud | Mature |
|  |  | VV_PEb19c02.b1 | 156731145 | Perlette | Bud | Mature |
|  |  | FAMU_USDA_FP_6765 | 51580906 | Vitis shuttleworthii | Entire tendril, leaves, bud, flowers | At blooming |
|  |  | FAMU_USDA_FP_7575 | 51581716 | Vitis shuttleworthii | Entire tendril, leaves, bud, flowers | At blooming |
|  |  | VVA028D02_392745 | 30321314 | Chardonnay | Leaf | Juvenile and adult |
|  |  | VVA028D02_391807 | 30320845 | Chardonnay | Leaf | Juvenile and adult |
|  |  | VVA028D02_55413 | 18459681 | Chardonnay | Leaf | Juvenile and adult |
|  |  | VVC039A10_140818 | 27584185 | Chardonnay | Berries | Mixed; 8, 9, 11, 13, 15, 16 weeks daf |
|  |  | VVH036H04_744725 | 71860395 | Cabernet Sauvignon | Nectary of flowers | 25 - modified E-L system |
|  |  | CAP0004_IF_D08 | 34548487 | Cabernet Sauvignon | Petiole | Onset of Veraison (berry softening) |
|  |  | CAB40002_IVa_Fa_D10 | 30299828 | Cabernet Sauvignon | Berries | Berry on stage II, 9 mm |
|  |  | WIN0510.C21_B13 | 110371569 | Cabernet Sauvignon | Flower, leaf and root | Flower, pre-anthesis; leaf, fully expanded; root, produced by air-layering |
|  |  | SCB03983 | 110730189 | Thompson-seedless | Inflorescence | Inflorescence with GA3 |
|  |  | S8B00494 | 110718294 | Thompson-seedless | Fruit | Fruits Veraison |
|  |  | CAP0004_IR_D08 | 34548573 | Cabernet Sauvignon | Petiole | Onset of Veraison (berry softening) |
|  |  | WIN1125.C21_H01 | 110419425 | Muscat Hamburg | Berries | Anthesis flower to prior to veraison |
|  |  | WIN1111.C21_L05 | 110415168 | Muscat Hamburg | Berries | Anthesis flower to prior to veraison |
| *VviMAPKKK54* | GSVIVT01032389001 | sT7aVVM021D12046 | 161719246 | Cabernet Sauvignon | Roots | 10 cm high plants grown in Magenta boxes |
|  |  | sT7aVVM_AER18G08 | 161708399 | Cabernet Sauvignon | Roots | 10 cm high plants grown in Magenta boxes |
|  |  | VVL010B02_676282 | 71878273 | Cabernet Sauvignon | Fruit with seeds removed | Mixed 36-38 - modified E-L system (Brix > 15) |
|  |  | VVL026H01_679156 | 71879710 | Cabernet Sauvignon | Fruit with seeds removed | Mixed 36-38 - modified E-L system (Brix > 15) |
|  |  | VVL010E08_676352 | 71878308 | Cabernet Sauvignon | Fruit with seeds removed | Mixed 36-38 - modified E-L system (Brix > 15) |
|  |  | VVL019C06_677836 | 71879050 | Cabernet Sauvignon | Fruit with seeds removed | Mixed 36-38 - modified E-L system (Brix > 15) |
|  |  | VVL036G04_680862 | 71880563 | Cabernet Sauvignon | Fruit with seeds removed | Mixed 36-38 - modified E-L system (Brix > 15) |
|  |  | VVL025E10_678934 | 71879599 | Cabernet Sauvignon | Fruit with seeds removed | Mixed 36-38 - modified E-L system (Brix > 15) |
|  |  | WIN094.C21_E24 | 110404639 | Cabernet Sauvignon | Pericarp | Fruit set to maturity |
|  |  | WIN117.C21_D22 | 110429607 | Muscat Hamburg | Berries | Anthesis flower to prior to veraison |
|  |  | WIN1148.C21_E03 | 110426961 | Muscat Hamburg | Berries | Anthesis flower to prior to veraison |
|  |  | WIN088.C21_F19 | 110403186 | Cabernet Sauvignon | Seed | Fruit set to maturity |
|  |  | VVI065H01_592734 | 71872392 | Cabernet Sauvignon | Inflorescence including flowers | 12 - modified E-L system |
|  |  | WIN056.C21_D16 | 110382036 | Cabernet Sauvignon | Flower, leaf and root | Flower, pre-anthesis; leaf, fully expanded; root, produced by air-layering |
|  |  | CSECS120H11_PREn0028 | 51051085 | Cabernet Sauvignon | Fruit | 28 - modified E-L system |
|  |  | CSECS005G06_PREu0032 | 34361824 | Cabernet Sauvignon | Fruit with seeds removed | 32 - modified E-L system |
|  |  | CA12LIO2IIFb_H06 | 26257121 | Cabernet Sauvignon | Leaf | Late season sample |
| *VviMAPKKK55* | GSVIVT01032487001 | WIN0541.C21_F12 | 122690062 | Cabernet Sauvignon | Flower, leaf and root | Flower, pre-anthesis; leaf, fully expanded; root, produced by air-layering |
|  |  | WIN0418.C21_H14 | 110369346 | Cabernet Sauvignon | Pericarp | Fruit set to maturity |
|  |  | WIN0537.C21_O02 | 110380858 | Cabernet Sauvignon | Flower, leaf and root | Flower, pre-anthesis; leaf, fully expanded; root, produced by air-layering |
|  |  | VVL051A04_683312 | 71881788 | Cabernet Sauvignon | Fruit with seeds removed | Mixed 36-38 - modified E-L system (Brix > 15) |
|  |  | VVL114D10_694194 | 71887229 | Cabernet Sauvignon | Fruit with seeds removed | Mixed 36-38 - modified E-L system (Brix > 15) |
|  |  | VVL114A05_694114 | 71887189 | Cabernet Sauvignon | Fruit with seeds removed | Mixed 36-38 - modified E-L system (Brix > 15) |
|  |  | VVL084B11_688942 | 71884603 | Cabernet Sauvignon | Fruit with seeds removed | Mixed 36-38 - modified E-L system (Brix > 15) |
|  |  | VVL155C07_701296 | 77589412 | Cabernet Sauvignon | Fruit with seeds removed | Mixed 36-38 - modified E-L system (Brix > 15) |
|  |  | EST 3220 | 22009248 | Shiraz | Fruit | Veraison |
|  |  | VVB190C01_430185 | 32247640 | Chardonnay | Leaf | Juvenile and adult |
|  |  | VVC025D06_394817 | 30329342 | Chardonnay | Berries | Mixed; 8, 9, 11, 13, 15, 16 weeks daf |
|  |  | V-B-126A01 | 33961981 | Vitis aestivalis/Norton | Leaf | Young leaf |
|  |  | WIN1121.C21_K15 | 110418221 | Muscat Hamburg | Berries | Anthesis flower to prior to veraison |
| *VviMAPKKK56* | GSVIVT01033779001 | sT7aVVM_AER56G12 | 161707075 | Cabernet Sauvignon | Roots | 10 cm high plants grown in Magenta boxes |
|  |  | VVB016A10_404047 | 32247031 | Chardonnay | Leaf | Juvenile and adult |
|  |  | VVB041C06_324006 | 30321464 | Chardonnay | Leaf | Juvenile and adult |
|  |  | VVB024D09_133062 | 27580407 | Chardonnay | Leaf | Juvenile and adult |
|  |  | VVB016A10_131604 | 27579678 | Chardonnay | Leaf | Juvenile and adult |
|  |  | VVB028C07_133388 | 27580570 | Chardonnay | Leaf | Juvenile and adult |
|  |  | VVB216C09_434739 | 32249917 | Chardonnay | Leaf | Juvenile and adult |
|  |  | VVB016A10_403367 | 32246691 | Chardonnay | Leaf | Juvenile and adult |
|  |  | VV_PEa06a08.b2 | 156725072 | Perlette | Bud | Mature |
|  |  | VVL004F10_675344 | 71877804 | Cabernet Sauvignon | Fruit with seeds removed | Mixed 36-38 - modified E-L system (Brix > 15) |
|  |  | WIN054.C21_H23 | 110381823 | Cabernet Sauvignon | Flower, leaf and root | Flower, pre-anthesis; leaf, fully expanded; root, produced by air-layering |
|  |  | C1G06170 | 110685448 | Carmenere | Fruit - bud - clusters |  |
| *VviMAPKKK57* | GSVIVT01034710001 | sT7aVVM002F06027 | 161712544 | Cabernet Sauvignon | Roots | 10 cm high plants grown in Magenta boxes |
|  |  | VVL064D05_685664 | 71882964 | Cabernet Sauvignon | Fruit with seeds removed | Mixed 36-38 - modified E-L system (Brix > 15) |
|  |  | VVL069C05_686476 | 71883370 | Cabernet Sauvignon | Fruit with seeds removed | Mixed 36-38 - modified E-L system (Brix > 15) |
|  |  | VVL128C09_696606 | 71888435 | Cabernet Sauvignon | Fruit with seeds removed | Mixed 36-38 - modified E-L system (Brix > 15) |
|  |  | VVL064D05_685664 | 71882964 | Cabernet Sauvignon | Fruit with seeds removed | Mixed 36-38 - modified E-L system (Brix > 15) |
|  |  | WIN0532.C21_D18 | 110379384 | Cabernet Sauvignon | Flower, leaf and root | Flower, pre-anthesis; leaf, fully expanded; root, produced by air-layering |
|  |  | VV_PEa06a08.b2 | 156725072 | Perlette | Bud | Mature |
|  |  | CAP0002_IF_D06 | 34547329 | Cabernet Sauvignon | Petiole | Onset of Veraison (berry softening) |
|  |  | CAP0002_IF_D06 | 34547329 | Cabernet Sauvignon | Petiole | Onset of Veraison (berry softening) |
| *VviMAPKKK58* | GSVIVT01034988001 | sT7aVVM028P20066 | 161720212 | Cabernet Sauvignon | Roots | 10 cm high plants grown in Magenta boxes |
|  |  | VV_PEd0016a08.b1 | 156736973 | Perlette | Bud | Young |
|  |  | VV_PEd0016h09.g1 | 156737144 | Perlette | Bud | Young |
|  |  | VVB165E07_411551 | 32270663 | Chardonnay | Leaf | Juvenile and adult |
|  |  | WIN1119.C21_F14 | 110417523 | Muscat Hamburg | Berries | Anthesis flower to prior to veraison |
|  |  | WIN0540.C21_K23 | 110382785 | Cabernet Sauvignon | Flower, leaf and root | Flower, pre-anthesis; leaf, fully expanded; root, produced by air-layering |
| *VviMAPKKK59* | GSVIVT01035409001 | sT7aVVM002F06027 | 161712544 | Cabernet Sauvignon | Roots | 10 cm high plants grown in Magenta boxes |
|  |  | CAB40005_IVa_Ra_H02 | 30301562 | Cabernet Sauvignon | Berries | Berry on stage II, 9 mm |
|  |  | CAB20002_IVa_Fa_C01 | 33402249 | Cabernet Sauvignon | Flower - Bloom | Bloom |
|  |  | CAB40005_IVa_Fa_H02 | 30301495 | Cabernet Sauvignon | Berries | Berry on stage II, 9 mm |
|  |  | CAB70001_IaR_D11 | 30303291 | Cabernet Sauvignon | Berries | Post-Veraison, 18-19 brix |
|  |  | CAB20002_IVa_Ra_C01 | 33402327 | Cabernet Sauvignon | Flower - Bloom | Bloom |
|  |  | CAbud0002_IVR_D04 | 34543449 | Cabernet Sauvignon | Bud | Pre-bloom (10-11 days before bloom) |
|  |  | CAbud0002_IIIF_D04 | 34543534 | Cabernet Sauvignon | Bud | Pre-bloom (10-11 days before bloom) |
|  |  | C1G06170 | 110685448 | Carmenere | Fruit - bud - clusters |  |
|  |  | WIN1145.C21_J03 | 110426077 | Muscat Hamburg | Berries | Anthesis flower to prior to veraison |
|  |  | CSECS057G10_VERu0035 | 34364117 | Cabernet Sauvignon | Fruit with seeds removed | 35 - modified E-L system |
|  |  | VVD104H06_369571 | 30130616 | Chardonnay | Berries | Mixed; 8, 9, 11, 13, 15, 16 weeks daf |
|  |  | VVD166G05_377053 | 30128159 | Chardonnay | Berries | Mixed; 8, 9, 11, 13, 15, 16 weeks daf |
|  |  | VVB016A10_131604 | 27579678 | Chardonnay | Leaf | Juvenile and adult |
|  |  | VVB075H06_333702 | 30324025 | Chardonnay | Leaf | Juvenile and adult |
|  |  | VVC044D07_141766 | 27584660 | Chardonnay | Berries | Mixed; 8, 9, 11, 13, 15, 16 weeks daf |
|  |  | WIN085.C21_A12 | 110397588 | Cabernet Sauvignon | Seed | Fruit set to maturity |
|  |  | WIN051.C21_L16 | 110370942 | Cabernet Sauvignon | Flower, leaf and root | Flower, pre-anthesis; leaf, fully expanded;  root, produced by air-layering |
|  |  | WIN102.C21_N21 | 110405303 | Muscat Hamburg | Pericarp | Fruit set to maturity |
|  |  | WIN0823.C21_J15 | 110400430 | Cabernet Sauvignon | Seed | Fruit set to maturity |
|  |  | WIN1128.C21_G04 | 110420400 | Muscat Hamburg | Berries | Anthesis flower to prior to veraison |
|  |  | WIN1012.C21_N23 | 110406779 | Muscat Hamburg | Pericarp | Fruit set to maturity |
| *VviMAPKKK60* | GSVIVT01036758001 | VVB026B05_404205 | 32247110 | Chardonnay | Leaf | Juvenile and adult |
|  |  | VVB026B05_133902 | 27580827 | Chardonnay | Leaf | Juvenile and adult |
|  |  | VVB026B05_403573 | 32246794 | Chardonnay | Leaf | Juvenile and adult |
|  |  | VVB035A11_134928 | 27581340 | Chardonnay | Leaf | Juvenile and adult |
|  |  | RR890915N0001_IIId_Rd_C04 | 33395024 | Vitis hybrid cultivar | Leaf |  |
|  |  | VV_PEb02c05.b1 | 156728369 | Perlette | Bud | Mature |
|  |  | RADIC01_000325 | 37185457 | Pinot Noir | Roots | Young root |
|  |  | FAMU_USDA_FP_2981 | 51577122 | Vitis shuttleworthii | Entire tendril, leaves,  bud, flowers | At blooming |
|  |  | WIN0515.C21_I23 | 110373239 | Cabernet Sauvignon | Flower, leaf and root | Flower, pre-anthesis; leaf, fully expanded; root, produced by air-layering |
|  |  | WIN0210.TB24.1_M24 | 110362476 | Cabernet Sauvignon | Flower, leaf and root | Flower, pre-anthesis; leaf, fully expanded; root, produced by air-layering |
|  |  | CAB20001_IIIa_Fa_A04 | 33401438 | Cabernet Sauvignon | Flower - Bloom | Bloom |
|  |  | WIN0535.C21_I03 | 110380422 | Cabernet Sauvignon | Flower, leaf and root | Flower, pre-anthesis; leaf, fully expanded; root, produced by air-layering |
| *VviMAPKKK61* | GSVIVT01037773001 | sT7aVVM_AER55D05 | 161709407 | Cabernet Sauvignon | Roots | 10 cm high plants grown in Magenta boxes |
|  |  | VVB014D12_131324 | 27579538 | Chardonnay | Leaf | Juvenile and adult |
|  |  | VVB125D04_404573 | 32265284 | Chardonnay | Leaf | Juvenile and adult |
|  |  | VVD002H09_127256 | 27586850 | Chardonnay | Berries | Mixed; 8, 9, 11, 13, 15, 16 weeks daf |
| *VviMAPKKK62* | GSVIVT01038760001 | VVI214A01_617490 | 77587495 | Cabernet Sauvignon | Inflorescence including  flowers | 12 - modified E-L system |
|  |  | WIN0519.C21_N22 | 110375512 | Cabernet Sauvignon | Flower, leaf and root | Flower, pre-anthesis; leaf, fully expanded; root, produced by air-layering |
|  |  | CAB40006_IVa_Ra_G09 | 30302119 | Cabernet Sauvignon | Berries | Berry on stage II, 9 mm |
|  |  | VVC023G11_137388 | 27582914 | Chardonnay | Berries | Mixed; 8, 9, 11, 13, 15, 16 weeks daf |
|  |  | VV_PEc02B02.r.ab1 | 156733155 | Perlette | Bud | Mature |
|  |  | CAbud0001_IF_F12 | 34542276 | Cabernet Sauvignon | Bud | Pre-bloom (10-11 days before bloom) |
|  |  | WIN0521.C21_K08 | 110376046 | Cabernet Sauvignon | Flower, leaf and root | Flower, pre-anthesis; leaf, fully expanded; root, produced by air-layering |
