# Supplementary material for: Mitogen-activated protein kinase cascades in Vitis vinifera
Source: Front Plant Sci. 2015 Jul 22;6:556. doi: 10.3389/fpls.2015.00556 (PMC4511077; doi:10.3389/fpls.2015.00556)
Supplement: Supplementary file 4 [file Table4.DOC]

**Supplementary Table 4.** **Expressed sequence taqs (ESTs) identified for MAPKKKK subfamily in *Vitis vinifera*.** The protein name, *Vitis* proteome 12x ID, GenBank ID, EST name, cultivar/tissue type, and development stage are given for each gene.

| **Name** | ***Vitis* 12X ID** | **EST Name** | **GenBank ID** | **Species/Cultivar** | **Tissue Type** | **Development Stage** |
| --- | --- | --- | --- | --- | --- | --- |
| *VvMAP4K1* | GSVIVT01012233001 | sT7aVVM009I16056 | 161713131 | Cabernet Sauvignon | Roots | 10 cm high plants grown in Magenta boxes |
|  |  | VVG045D03_759889 | 71858432 | Cabernet Sauvignon | Cell Suspension Culture |  |
|  |  | WIN1120.C21_I23 | 122691819 | Muscat Hamburg | Berries | Anthesis flower to prior to veraison |
|  |  | VVI015F05_585280 | 71868665 | Cabernet Sauvignon | Inflorescence including  flowers | 12 - modified E-L system |
|  |  | WIN024.C21_O16 | 110364485 | Cabernet Sauvignon | Flower, leaf and root | Flower, pre-anthesis; leaf, fully expanded;  root, produced by air-layering |
| *VvMAP4K2* | GSVIVT01013739001 | VVC049C12_395587 | 30329727 | Chardonnay | Berries | Mixed; 8, 9, 11, 13, 15, 16 weeks daf |
|  |  | VVD012A02_344325 | 30129196 | Chardonnay | Berries | Mixed; 8, 9, 11, 13, 15, 16 weeks daf |
|  |  | VVC055F04_147986 | 27585568 | Chardonnay | Berries | Mixed; 8, 9, 11, 13, 15, 16 weeks daf |
|  |  | VVC008G05_416429 | 32245862 | Chardonnay | Berries | Mixed; 8, 9, 11, 13, 15, 16 weeks daf |
|  |  | WIN1014.C21_M12 | 110407331 | Muscat Hamburg | Pericarp | Fruit set to maturity |
|  |  | VVC008G05_415553 | 32245424 | Chardonnay | Berries | Mixed; 8, 9, 11, 13, 15, 16 weeks daf |
|  |  | VVC049C12_396775 | 30330321 | Chardonnay | Berries | Mixed; 8, 9, 11, 13, 15, 16 weeks daf |
|  |  | SCB07102 | 110731131 | Thompson-seedless | Inflorescence | Inflorescence with GA3 |
|  |  | WIN013.BR_K16 | 110357004 | Cabernet Sauvignon | Pericarp | Fruit set to maturity |
|  |  | VRK351T7 | 49143403 | Vitis riparia | Bud | Paradormant |
|  |  | VVG037A10_758327 | 71857651 | Cabernet Sauvignon | Cell Suspension Culture |  |
|  |  | CAbud0003_IR_H06 | 34544077 | Cabernet Sauvignon | Bud | Pre-bloom (10-11 days before bloom) |
|  |  | EST 5245 | 22011273 | Shiraz | Fruit | Ripening Stage |
|  |  | VVD145B11_374811 | 30127121 | Chardonnay | Berries | Mixed; 8, 9, 11, 13, 15, 16 weeks daf |
|  |  | VVD173H10_378093 | 30124752 | Chardonnay | Berries | Mixed; 8, 9, 11, 13, 15, 16 weeks daf |
|  |  | VV_PEa19g09.g1 | 156727149 | Perlette | Bud | Mature |
| *VvMAP4K3* | GSVIVT01014297001 | FAMU_USDA_FP_3144 | 51577285 | Vitis shuttleworthii | Entire tendril, leaves, bud, flowers | At blooming |
|  |  | EST 8953 | 32459870 | Chardonnay | Fruit without seeds | Green stage |
|  |  | EST 9020 | 32459937 | Chardonnay | Fruit without seeds | Green stage |
|  |  | S2B24172 | 110702322 | Thompson-seedless | Bud |  |
|  |  | WIN0572.C21_K17 | 110391131 | Cabernet Sauvignon | Flower, leaf and root | Flower, pre-anthesis; leaf, fully expanded; root, produced by air-layering |
|  |  | VVH059H01_748881 | 71861562 | Cabernet Sauvignon | Nectary of flowers | 25 - modified E-L system |
|  |  | VVB064C11_326962 | 30322942 | Chardonnay | Leaf | Juvenile and adult |
|  |  | SCB01402 | 110729745 | Thompson-seedless | Inflorescence | Inflorescence with GA3 |
|  |  | CSECS210E05_5_PREn0028 | 87585907 | Cabernet Sauvignon | Fruit | 28 - modified E-L system |
|  |  | VVD045C08_352369 | 30135530 | Chardonnay | Berries | Mixed; 8, 9, 11, 13, 15, 16 weeks daf |
|  |  | C3B01081 | 110692917 | Carmenere | Cluster |  |
|  |  | SBB04034 | 110727216 | Thompson-seedless | Inflorescence |  |
|  |  | VVB163G10_411255 | 32270515 | Chardonnay | Leaf | Juvenile and adult |
| *VvMAP4K4* | GSVIVT01016074001 | VVB129A09_405445 | 32267610 | Chardonnay | Leaf | Juvenile and adult |
|  |  | VVG037A10_758327 | 71857651 | Cabernet Sauvignon | Cell Suspension Culture |  |
|  |  | VVL145C05_699562 | 71889913 | Cabernet Sauvignon | Fruit with seeds removed | Mixed 36-38 - modified E-L system (Brix > 15) |
|  |  | WIN0411.C21_M18 | 110366978 | Cabernet Sauvignon | Pericarp | Fruit set to maturity |
|  |  | VVL014E10_677036 | 71878650 | Cabernet Sauvignon | Fruit with seeds removed | Mixed 36-38 - modified E-L system (Brix > 15) |
|  |  | VVL014D05_677008 | 71878636 | Cabernet Sauvignon | Fruit with seeds removed | Mixed 36-38 - modified E-L system (Brix > 15) |
|  |  | VVL008B12_675946 | 71878105 | Cabernet Sauvignon | Fruit with seeds removed | Mixed 36-38 - modified E-L system (Brix > 15) |
|  |  | VVD162E07_376363 | 30127814 | Chardonnay | Berries | Mixed; 8, 9, 11, 13, 15, 16 weeks daf |
|  |  | VVG036E12_758239 | 71857607 | Cabernet Sauvignon | Cell Suspension Culture |  |
| *VvMAP4K5* | GSVIVT01019643001 | VVL036F03_680838 | 71880551 | Cabernet Sauvignon | Fruit with seeds removed | Mixed 36-38 - modified E-L system (Brix > 15) |
|  |  | sT7aVVM_AER50C08 | 161707044 | Cabernet Sauvignon | Roots | 10 cm high plants grown in Magenta boxes |
|  |  | VV_PEb11b11.b1 | 156729830 | Perlette | Bud | Mature |
|  |  | sT7aVVM_AER52F01 | 161706933 | Cabernet Sauvignon | Roots | 10 cm high plants grown in Magenta boxes |
|  |  | VVB125C05_404553 | 32265274 | Chardonnay | Leaf | Juvenile and adult |
|  |  | VVH020G03_741863 | 71862547 | Cabernet Sauvignon | Nectary of flowers | 25 - modified E-L system |
|  |  | sT7aVVM_AER54F01 | 161710935 | Cabernet Sauvignon | Roots | 10 cm high plants grown in Magenta boxes |
|  |  | VVL027E12_679276 | 71879770 | Cabernet Sauvignon | Fruit with seeds removed | Mixed 36-38 - modified E-L system (Brix > 15) |
|  |  | VVB165H01_411609 | 32270692 | Chardonnay | Leaf | Juvenile and adult |
|  |  | VVL031B04_679908 | 71880086 | Cabernet Sauvignon | Fruit with seeds removed | Mixed 36-38 - modified E-L system (Brix > 15) |
|  |  | VVL040F02_681504 | 71880884 | Cabernet Sauvignon | Fruit with seeds removed | Mixed 36-38 - modified E-L system (Brix > 15) |
|  |  | VVL010F03_676364 | 71878314 | Cabernet Sauvignon | Fruit with seeds removed | Mixed 36-38 - modified E-L system (Brix > 15) |
|  |  | VVL005C08_675452 | 71877858 | Cabernet Sauvignon | Fruit with seeds removed | Mixed 36-38 - modified E-L system (Brix > 15) |
|  |  | VVL020H01_678102 | 71879183 | Cabernet Sauvignon | Fruit with seeds removed | Mixed 36-38 - modified E-L system (Brix > 15) |
|  |  | VVB188F12_429909 | 32247502 | Chardonnay | Leaf | Juvenile and adult |
|  |  | VVB201B08_432187 | 32248641 | Chardonnay | Leaf | Juvenile and adult |
|  |  | VVB189A08_429973 | 32247534 | Chardonnay | Leaf | Juvenile and adult |
|  |  | VVB143C10_407535 | 32268655 | Chardonnay | Leaf | Juvenile and adult |
|  |  | VVB123C04_383791 | 30328197 | Chardonnay | Leaf | Juvenile and adult |
|  |  | VB129A09_405445 | 32267610 | Chardonnay | Leaf | Juvenile and adult |
|  |  | VVB166B02_411653 | 32270714 | Chardonnay | Leaf | Juvenile and adult |
|  |  | VVB109H01_342065 | 30327011 | Chardonnay | Leaf | Juvenile and adult |
|  |  | VVD023A12_345797 | 30132243 | Chardonnay | Berries | Mixed; 8, 9, 11, 13, 15, 16 weeks daf |
|  |  | VVB093D06_340611 | 30326284 | Chardonnay | Leaf | Juvenile and adult |
|  |  | VRK351T7 | 49143403 | Vitis riparia | Bud | Paradormant |
|  |  | VRK351 | 49143402 | Vitis riparia | Bud | Paradormant |
|  |  | C2B04929 | 110689637 | Carmenere | Bud - cluster |  |
|  |  | CAP0003_IIIR_G04 | 34548430 | Cabernet Sauvignon | Petiole | Onset of Veraison (berry softening) |
|  |  | VV_PEb11b11.g1 | 156729831 | Perlette | Bud | Mature |
|  |  | CAP0003_IIIF_G04 | 34548092 | Cabernet Sauvignon | Petiole | Onset of Veraison (berry softening) |
|  |  | CAbud0005_IF_D08 | 34544637 | Cabernet Sauvignon | Bud | Pre-bloom (10-11 days before bloom) |
|  |  | GERMO01_000034 | 37189040 | Regent | Shoot tip | Growing shoot tip (ca. 1 cm long) |
|  |  | S5B06527 | 110710295 | Thompson-seedless | Fruit | Fruits 7-9 mm treated with GA3 |
|  |  | SCB07102 | 110731131 | Thompson-seedless | Inflorescence | Inflorescence with GA3 |
|  |  | WIN026.TB24.1_E21 | 110361229 | Cabernet Sauvignon | Flower, leaf and root | Flower, pre-anthesis; leaf, fully expanded; root, produced by air-layering |
|  |  | WIN1122.C21_M08 | 110418566 | Muscat Hamburg | Berries | Anthesis flower to prior to veraison |
| *VvMAP4K7* | GSVIVT01032461001 | CA23EI02IVRb_F07 | 28959739 | Cabernet Sauvignon | Leaf | Mid-season leaf material |
|  |  | VVL036C12_680790 | 71880527 | Cabernet Sauvignon | Fruit with seeds removed | Mixed 36-38 - modified E-L system (Brix > 15) |
|  |  | VVL014D05_677008 | 71878636 | Cabernet Sauvignon | Fruit with seeds removed | Mixed 36-38 - modified E-L system (Brix > 15) |
|  |  | VVL008B12_675946 | 71878105 | Cabernet Sauvignon | Fruit with seeds removed | Mixed 36-38 - modified E-L system (Brix > 15) |
|  |  | VVA027H10_55365 | 18459657 | Chardonnay | Leaf | Juvenile and adult |
|  |  | WIN012.BR_F24 | 110357330 | Cabernet Sauvignon | Pericarp | Fruit set to maturity |
|  |  | VVG037A10_758327 | 71857651 | Cabernet Sauvignon | Cell Suspension Culture |  |
|  |  | VRK351T7 | 49143403 | Vitis riparia | Bud | Paradormant |
|  |  | WIN0414.C21_A10 | 110367697 | Cabernet Sauvignon | Pericarp | Fruit set to maturity |
|  |  | WIN013.BR_K16 | 110357004 | Cabernet Sauvignon | Pericarp | Fruit set to maturity |
